# Cord-Derived Stem Cells for Pediatric Autism Spectrum Disorder: Biological Rationale and Source-Specific Trial Design

**DOI:** 10.3390/ijms27198634

**Published:** 2026-09-26

**Authors:** Chang-Eui Hong, Su-Yun Lyu

**Affiliations:** 1 College of Pharmacy, Sunchon National University, Suncheon 57922, Republic of Korea; gruni80@scnu.ac.kr; 2 Smart Bio Research Institute, Sunchon National University, Suncheon 57922, Republic of Korea

**Keywords:** autism spectrum disorder, umbilical cord-derived mesenchymal stem cells, umbilical cord blood, neuroinflammation, immunomodulation, cell therapy, source-specific clinical trials

## Abstract

Autism spectrum disorder (ASD) affects approximately 1 in 31 children, yet no pharmacological agent targets its core symptoms, leaving a substantial therapeutic gap. Umbilical cord-derived mesenchymal stem/stromal cells (UC-MSCs) have emerged as a biologically rational candidate, because their immunomodulatory, anti-inflammatory, and neurotrophic properties align with the chronic neuroinflammation, peripheral immune dysregulation, and microglial overactivation that characterize ASD pathophysiology. This review synthesizes the mechanistic rationale, tissue-source biology, manufacturing and characterization standards, preclinical evidence, and clinical trial landscape for cord-derived cell therapy in pediatric ASD, and examines administration routes, post-treatment monitoring, and long-term safety. Across five published clinical trials employing cord blood mononuclear cells, autologous and allogeneic umbilical cord blood, UC-MSCs, bone marrow-derived MSCs, and Wharton’s jelly-derived MSCs, short-term safety is the most consistent finding, whereas efficacy remains unresolved owing to small sample sizes, single-arm designs, and heterogeneous outcome measures; a completed randomized Phase II trial of a cord tissue MSC product, reported to date only in the trial registry, did not meet its primary endpoint. The central argument advanced here is that the proposed active component of cord-derived therapy is the purified MSC population rather than the heterogeneous mononuclear cell fraction; this proposition is supported by the CD14^+^ monocyte mechanism and by the one pediatric trial in which purified UC-MSC was added to a mononuclear preparation, but it has not been confirmed in any clinical study and remains a hypothesis. Advancing the field requires dedicated source-specific randomized controlled trials integrating product standardization, patient stratification, and dose–route–timing optimization.

## 1. Introduction

Autism spectrum disorder (ASD) is a neurodevelopmental condition defined by persistent deficits in social communication and social interaction, together with restricted, repetitive patterns of behavior, interests, or activities [1,2]. Although early signs may emerge in the first two years of life, a definitive diagnosis is typically established by age four to five through standardized behavioral assessment [3]. The most recent surveillance data from the United States (US) Centers for Disease Control and Prevention (CDC) Autism and Developmental Disabilities Monitoring (ADDM) Network reported an ASD prevalence of 32.2 per 1000 among 8-year-old children in 2022, equivalent to approximately one in 31, with a male-to-female ratio of 3.4:1 [4]. This figure represents a continued upward trajectory from 27.6 per 1000 reported in the 2020 surveillance year [5]. Globally, a systematic review encompassing data from multiple continents estimated a median prevalence of approximately 1 in 100 children, with considerable regional variability [6,7]. The etiology of ASD is complex and multifactorial, involving the interplay of genetic susceptibility, epigenetic modifications, and prenatal environmental exposure [2].

Despite decades of research, therapeutic options for ASD remain limited. Current management relies primarily on behavioral interventions, including applied behavior analysis (ABA) and early intensive developmental programs such as the Early Start Denver Model, which can improve social communication and adaptive skills but require sustained, resource-intensive implementation [3]. In terms of pharmacotherapy, only two medications-risperidone and aripiprazole-have received US Food and Drug Administration (FDA) approval for use in ASD, and both are indicated exclusively for the management of irritability and aggression rather than core social communication deficits [1,8,9]. The estimated lifetime cost of supporting an individual with ASD ranges from $1.4 million for those without co-occurring intellectual disability (ID) to $2.4 million for those with ID in the US [10]. The absence of disease-modifying therapies that address the core neurodevelopmental deficits of ASD constitutes a critical unmet medical need [1]. Addressing this gap requires a deeper understanding of the pathophysiological mechanisms underlying ASD, which may reveal actionable therapeutic targets.

In this regard, a growing body of evidence implicates neuroinflammation and immune dysregulation as contributing pathophysiological mechanisms in a substantial subset of individuals with ASD. Postmortem analyses of brain tissue from individuals with ASD have revealed marked activation of microglia and astroglia in the cerebral cortex, white matter, and cerebellum, accompanied by a proinflammatory cytokine profile characterized by elevated monocyte chemoattractant protein (MCP)-1 and transforming growth factor-beta 1 (TGF-β1) [11]. Peripheral immune abnormalities, including elevated levels of interleukin (IL)-1 beta (1β), IL-6, and tumor necrosis factor-alpha (TNF-α), altered T-cell subsets, and the presence of autoantibodies, have been consistently documented in children with ASD [12,13]. Prenatal immune insults, particularly maternal immune activation (MIA) during gestation, have also been implicated as environmental risk factors for ASD development [14,15]. These converging lines of evidence—from neuropathology, peripheral biomarkers, and prenatal immune programming—provide a rationale for exploring immunomodulatory cell-based therapies as a potential intervention strategy [16].

Mesenchymal stem/stromal cells (MSCs) have attracted considerable attention in this context owing to their well-characterized immunomodulatory, anti-inflammatory, and neurotrophic properties [17,18]. MSCs can suppress the proliferation of effector T cells, promote the expansion of regulatory T cells (Tregs), shift macrophage polarization toward an anti-inflammatory phenotype, and secrete neuroprotective factors such as brain-derived neurotrophic factor (BDNF) and insulin-like growth factor-1 (IGF-1) [17,19]. These paracrine mechanisms, rather than direct cell replacement, are considered the principal basis for the therapeutic potential of MSCs in neurodevelopmental disorders [18]. Over the past decade, a growing number of preclinical and early-phase clinical studies have examined the effects of MSC-based interventions in ASD, employing diverse cell sources including bone marrow (BM), adipose tissue (AD), umbilical cord blood (UCB), and umbilical cord tissue [20,21,22].

Several recent reviews have summarized the broader landscape of cell therapy for ASD [17,18,23]. However, these reviews addressed MSC therapy in a source-agnostic manner, without systematically evaluating the distinctive biological and practical advantages of any single tissue source. This represents a consequential gap, particularly given that ASD is predominantly a pediatric condition. Unlike bone marrow-derived MSCs (BM-MSCs) or adipose tissue-derived MSCs (AD-MSCs), which require invasive harvesting procedures that are difficult to justify in young children, umbilical cord-derived MSCs (UC-MSCs) are isolated from tissue that would otherwise be discarded at birth, offering a non-invasive, ethically unproblematic, and scalable cell source [24,25]. UC-MSCs proliferate more rapidly than BM-MSCs and do not express MHC class II, and their therapeutic effects are attributed to paracrine rather than replacement mechanisms [24,26]. These source-specific attributes provide a rationale for evaluating UC-MSCs in pediatric ASD, although this rationale rests on manufacturing and mechanistic characteristics rather than on comparative clinical evidence, and no review to date has systematically examined the preclinical and clinical evidence base for UC-MSCs and their derivatives in this indication. The present review aims to fill this gap by providing a source-specific analysis of UC-MSC therapy for pediatric ASD. We first examine the immunopathological rationale linking neuroinflammation to ASD (Section 2) and evaluate the biological properties of UC-MSCs relevant to this indication (Section 3). We then discuss manufacturing considerations for clinical-grade products (Section 4) before synthesizing preclinical (Section 5) and clinical evidence (Section 6), the latter including a dedicated examination of the frequently conflated distinction between UCB and UC-MSC therapies. Two practical dimensions largely underrepresented in prior reviews are then addressed: administration routes (Section 7) and post-treatment monitoring (Section 8). We conclude with current challenges and future directions (Section 9).

Throughout this review, UC-MSC denotes human UC-MSC unless otherwise specified, and where UC-MSC-specific evidence is unavailable we therefore incorporate principles derived from MSC studies in other neurological conditions and from other MSC tissue sources, with explicit acknowledgment of cross-source and cross-indication extrapolation where applied. The argument advanced here is one of priority rather than of demonstrated superiority: the properties that distinguish UC-MSC from other cell sources justify testing it first in a comparative trial, and they do not by themselves establish that it produces better clinical outcomes than umbilical cord blood or than MSCs from other tissues. Statements of source-specific advantage in the sections that follow refer to manufacturing, expansion, and mechanistic properties measured in vitro or in animal models, and no clinical comparison supporting superiority has yet been published.

The evidence base was identified through searches of PubMed/MEDLINE, supplemented by ClinicalTrials.gov for registered interventional studies, by CrossRef for records not indexed in PubMed, and by manual screening of reference lists, from database inception to July 2026. Search terms combined population, intervention, and mechanism concepts: (“autism” OR “autism spectrum disorder” OR “ASD”) AND (“mesenchymal stem cell” OR “mesenchymal stromal cell” OR “MSC” OR “umbilical cord” OR “Wharton’s jelly” OR “cord blood”) AND (“therapy” OR “transplantation” OR “clinical trial” OR “immunomodulation”).

Peer-reviewed articles were included when they reported original preclinical or clinical data on umbilical cord-derived cell therapy, mechanistic evidence on MSC immunomodulatory or neurotrophic action, or manufacturing and regulatory standards for clinical-grade MSC products; non-English records, sources without primary data or mechanistic synthesis, and indications unrelated to neurodevelopmental or neuroinflammatory pathophysiology were excluded. Because the distinction between cord blood-derived and cord tissue-derived products is central to this review, the cell source of every cited interventional study was confirmed against the primary report, with the full-text Methods consulted where the abstract was ambiguous. As a narrative review, no formal risk-of-bias assessment or quantitative synthesis was undertaken. The final citation set comprises 189 references spanning 2002 to 2026, 72% published in 2016 or later. The final citation set comprises 188 references spanning 2002 to 2026, 74% published in 2016 or later.

## 2. Neuroinflammation and Immune Dysregulation in ASD: Rationale for MSC Therapy

The therapeutic rationale for MSCs in ASD is grounded in evidence that neuroinflammation and immune dysregulation are associated with its pathophysiology. The nature of that relationship should be stated at the outset. The findings reviewed below are consistent, replicated, and derived from postmortem tissue, in vivo imaging, peripheral blood, and animal models, but with the exception of the maternal immune activation models they are observational, and none of them establishes that immune activation causes the core features of ASD rather than accompanying them or arising as a consequence of other neurodevelopmental processes. Immune activation may equally represent a downstream response, a marker of a subgroup, or a modifier of severity. This distinction does not weaken the case for immunomodulatory intervention, which requires only that the immune abnormalities be present and modifiable, but it does mean that clinical benefit from such intervention cannot be predicted from pathophysiological reasoning alone. This section synthesizes the evidence across central, peripheral, and prenatal domains before considering how the resulting immune landscape aligns with the immunomodulatory actions of MSCs.

### 2.1. Microglia Activation and Neuroglial Abnormalities

The earliest neuropathological evidence linking neuroinflammation to ASD came from postmortem analyses demonstrating marked activation of microglia and astroglia across multiple brain regions, including the cerebral cortex, white matter, and cerebellum [11]. Cytokine profiling of these tissues revealed elevated levels of monocyte chemoattractant protein-1 (MCP-1) and TGF-β1, with the MCP-1 elevation attributed by the authors to microglial activation and monocyte recruitment, while cerebrospinal fluid (CSF) samples exhibited a distinct proinflammatory profile [11]. Quantitative stereological analysis of the dorsolateral prefrontal cortex in postmortem ASD cases similarly documented microglial activation together with increased microglial density in the gray matter [27]. These findings were subsequently corroborated by in vivo positron emission tomography (PET) imaging, which demonstrated significantly increased binding of the microglial marker ^11^C-PK11195 in the cerebellum, midbrain, fusiform gyri, and anterior cingulate cortex of young adults with ASD compared with age- and Intelligence Quotient (IQ)-matched controls [28]. The pattern of microglial activation was not altered in its regional distribution but was augmented in magnitude, suggesting a state of chronically heightened, rather than fundamentally aberrant, innate immune surveillance in the ASD brain [28]. Whether this sustained microglial activation is a primary driver of synaptic dysfunction or a secondary response to other neurodevelopmental perturbations remains an active area of investigation [13].

### 2.2. Peripheral Immune Dysregulation and Cytokine Profiles

Immune abnormalities in ASD extend beyond the central nervous system (CNS). Peripheral blood analyses of children with ASD have consistently demonstrated a skewed cytokine milieu characterized by elevated proinflammatory mediators, including IL-1β, IL-6, and TNF-α, alongside reduced levels of anti-inflammatory cytokines such as IL-10 and TGF-β1 [12]. At the cellular level, children with ASD exhibit altered T-cell activation profiles, with increased production of granulocyte-macrophage colony-stimulating factor (GM-CSF) and TNF-α following mitogenic stimulation, and a concomitant reduction in T-cell surface activation markers cluster of differentiation (CD) 134 and CD25 [29]. These peripheral immune perturbations track clinical severity: increased proinflammatory cytokine levels have been positively correlated with greater impairments in core ASD features, including social communication deficits and repetitive behaviors, while GM-CSF and T-helper (Th) 2 cytokine production were associated with better cognitive and adaptive function [29]. These are cross-sectional correlations and do not establish the direction of the relationship. The presence of circulating autoantibodies reactive to neuronal and brain-specific antigens in a significant proportion of individuals with ASD further supports the hypothesis of systemic immune dysregulation contributing to neural pathology [12].

### 2.3. MIA, Prenatal Risk, and Blood–Brain Barrier (BBB) Compromise

Prenatal immune insults represent another convergent pathway linking immune dysfunction to ASD. Epidemiological studies have identified maternal infection during pregnancy as a risk factor for ASD in offspring, and animal models of MIA have demonstrated that gestational immune challenge alone is sufficient to produce ASD-like behavioral phenotypes and cortical malformations in progeny [14]. Mechanistically, Choi et al. demonstrated that maternal retinoic acid receptor-related orphan nuclear receptor gamma t (RORγt^+^) Th17 cells are required for MIA-induced cortical abnormalities and behavioral deficits in offspring, and that their effector cytokine IL-17a is both necessary and sufficient—intraventricular delivery of IL-17a into the fetal brain in the absence of maternal immune activation reproduced the cortical and behavioral phenotypes—providing a direct molecular link between maternal inflammation and fetal brain development [15]. The integrity of the BBB itself may also be compromised in ASD; analysis of postmortem brain tissue and duodenal biopsies from individuals with ASD has revealed altered expression of tight junction components, including increased gene expression of claudin-5 and claudin-12, alongside upregulated neuroinflammation markers such as translocator protein (TSPO) and matrix metalloproteinase (MMP)-9, although claudin-12 protein was reduced rather than increased in the same cortical samples, indicating that the tight junction changes are not uniformly in the direction of increased permeability [30]. A permeable BBB could facilitate the translocation of peripheral inflammatory mediators and autoantibodies into the CNS, thereby perpetuating neuroglial activation and synaptic dysfunction [13,30]. This sequence remains a proposed mechanism; the tight junction and neuroinflammation marker changes were measured in postmortem tissue and cannot establish their temporal relationship to the neurodevelopmental process.

### 2.4. Bridging Immune Pathophysiology to MSC-Based Intervention

The converging evidence outlined above, comprising chronic microglial activation, peripheral cytokine dysregulation, maternal immune programming, and BBB compromise, collectively delineates a neuroimmune substrate in ASD that is amenable to immunomodulatory intervention [16]. MSCs are positioned to address several facets of this pathophysiology simultaneously, acting through paracrine mediators that suppress effector T-cell proliferation [31,32], promote regulatory T-cell expansion [19], and shift microglial polarization from the proinflammatory M1 toward the anti-inflammatory M2 phenotype [16,17], while releasing neurotrophic factors that may support neuronal survival, synaptic plasticity, and cerebral angiogenesis [17]. The molecular axes underlying these effects are examined in Section 3.4 and the neurotrophic and angiogenic capacities in Section 3.5. This dual capacity provides the mechanistic rationale for investigating MSC-based therapies in ASD, and as discussed in the following section, the choice of MSC tissue source becomes a critical consideration when the target patient population is predominantly pediatric.

## 3. Source-Specific Considerations for Cell Selection in Pediatric ASD

Having outlined the neuroimmune rationale for MSC therapy, this section examines the properties that make UC-MSCs a candidate source for the pediatric ASD population, none of which has yet been shown to confer superior clinical efficacy. After surveying the major MSC tissue sources, it addresses the practical constraints of BM-MSCs and AD-MSCs in children, the anatomical compartments and the immunomodulatory, neurotrophic, and angiogenic properties that distinguish UC-MSCs, and the heterogeneity and standardization challenges that remain.

### 3.1. Overview of MSC Tissue Sources

MSCs are multipotent progenitor cells originally identified in the BM stroma as fibroblast-like adherent cells capable of supporting hematopoiesis and differentiating into multiple mesodermal lineages. The International Society for Cellular Therapy (ISCT) established minimal criteria for defining human MSCs: plastic adherence under standard culture conditions, expression of CD73, CD90, and CD105 with concomitant absence of the hematopoietic markers CD34, CD45, CD14 or CD11b, CD79α or CD19, and human leukocyte antigen (HLA)-DR isotype (HLA-DR), and the capacity for trilineage differentiation into osteoblasts, adipocytes, and chondroblasts in vitro [33]. Beyond these shared defining features, MSCs can be isolated from a range of adult and perinatal tissues, each yielding cells with overlapping yet source-dependent biological characteristics [34,35].

Three tissue sources have been most extensively investigated for therapeutic application: BM, AD, and the umbilical cord, yielding BM-MSCs, AD-MSCs, and UC-MSCs, respectively. Throughout this review, UC-MSCs denote MSCs isolated from the cord tissue itself—predominantly the Wharton’s jelly (WJ) compartment, with minor contributions from the perivascular (PV) and cord-lining regions-—and are distinct from UCB, a hematopoietic mononuclear product that is considered separately in Section 6. Although MSCs from all three sources satisfy the ISCT surface-marker and differentiation criteria, they differ in biologically and clinically relevant properties, including proliferative capacity, immunophenotype, and paracrine secretome composition, whereas immunosuppressive activity has been reported as comparable in some comparisons and as highest in BM-MSCs in others [35,36].

These source-dependent differences are not trivial. In a head-to-head comparison of BM-, AD-, and WJ-MSCs expanded under clinically relevant conditions, WJ-MSCs displayed the highest proliferative capacity, reaching a cumulative population doubling of 12.3 at passage 3 versus 6 for BM-MSCs, with a population doubling time of approximately 21 h compared with 99 h for BM-MSCs [36]. This comparison also revealed source-dependent secretome profiles of direct relevance to neural applications: BM-MSCs secreted the lowest content of neurotrophic factors, whereas WJ-MSCs produced significantly higher hepatocyte growth factor (HGF), fibroblast growth factor (FGF)-2, nerve growth factor (NGF), and BDNF than the other two sources, with HGF secretion three times that of AD-MSCs and approximately nine times that of BM-MSCs; nonetheless, the secretomes of all three sources stimulated neurite outgrowth of dorsal root ganglion neurons [36]. UC-MSCs further display superior migration toward activated lymphocytes in mixed lymphocyte reaction assays relative to BM- and AD-MSCs, a property relevant to their functional responsiveness under inflammatory conditions [37]. These distinctions underscore the rationale for treating MSC tissue origin as a variable of therapeutic significance rather than regarding MSCs as a monolithic population. Table 1 compares the three MSC sources and UCB across biological properties, procurement, manufacturing parameters, and the clinical evidence available in pediatric ASD.

### 3.2. Practical Constraints of BM-MSC and AD-MSC in Pediatric Settings

While source-specific differences in MSC properties are biologically meaningful, the practical feasibility of obtaining clinical-grade cells from different tissues is equally critical in pediatric applications. The harvesting procedures, age-dependent biological variability, and procedural risks associated with BM-MSCs and AD-MSCs raise considerations that are particularly relevant when the target patient population consists of young children with ASD, in whom invasive procedures must be justified by clear therapeutic benefit [49].

#### 3.2.1. Limitations of BM-MSCs

BM-MSCs require harvest via posterior iliac crest aspiration, a procedure that in young children almost invariably necessitates general anesthesia or deep procedural sedation [39]. A secondary analysis of the Anaesthesia PRactice In Children Observational Trial (APRICOT) dataset encompassed 893 children who underwent 915 BM aspiration or lumbar puncture procedures. It reported a severe adverse event rate of 1.7% and a respiratory complication rate of 1.1%. Prematurity (relative risk [RR] 4.98, 95% confidence interval [CI] 1.10–22.57), endotracheal intubation (RR 6.80, 95% CI 1.66–27.70), and emergency indication (RR 3.99, 95% CI 1.14–13.96) were independent risk factors for respiratory complications [41]. A multi-institutional pediatric oncology survey has similarly documented persistent issues with pain management efficacy and safety during BM aspiration and biopsy in children, with adverse events occurring across institutions and high-risk subgroups disproportionately affected [50]. Rare but serious infectious complications have also been described, including the development of iliopsoas abscess and sacroiliac osteomyelitis following an otherwise uncomplicated aspiration [42]. Beyond procedural risks, subjecting children with ASD—a population frequently characterized by sensory sensitivities to medical procedures—to invasive harvesting raises additional practical and ethical concerns.

Compounding these procedural concerns, BM-MSCs exhibit age-dependent decline in proliferative and differentiation capacity. In comparative studies, BM-MSCs from child donors aged 0–12 years retained fibroblast-like morphology through higher passage numbers and demonstrated greater proliferation than cells from adult (25–50 years) or elderly (over 60 years) donors, and adipogenic, osteogenic, and neurogenic differentiation potential decreased with increasing donor age, although chondrogenic potential was unchanged [43]. Paradoxically, this aging effect would favor pediatric donors as an optimal autologous source, yet the ethical justification for subjecting a young child with ASD to BM aspiration-an invasive procedure with no direct diagnostic benefit and no established therapeutic efficacy in this indication-remains difficult to defend [39]. The absence of transparent, harmonized guidelines for BM aspiration in children outside oncologic indications further complicates routine clinical application [39]. These considerations collectively limit the scalability of BM-MSC-based therapies for pediatric ASD and motivate the exploration of less invasive cell sources.

#### 3.2.2. Limitations of AD-MSCs

AD-MSCs face distinct procedural and biological constraints in pediatric application. Lipoaspiration in young children is generally restricted by regulatory and ethical considerations, and dedicated AD-MSC harvest for therapeutic purposes alone is rarely justified absent a concurrent surgical indication; in a representative pediatric application, Tauer et al. demonstrated that AD-derived stromal vascular fraction could be successfully obtained from pediatric osteogenesis imperfecta patients during surgical correction procedures, illustrating that pediatric AD-MSC procurement is largely feasible only when integrated with an existing surgical procedure [40]. Comparative analyses under clinically relevant culture conditions have further shown that AD-MSCs display lower immunomodulatory activity than BM-MSCs and exhibit distinct neurotrophic factor secretion profiles compared with WJ-MSCs, indicating that source-specific functional differences exist even among the major MSC tissue origins [36].

Beyond procedural and source-related considerations, the use of autologous AD-MSCs in ASD raises a specific concern that does not apply equivalently to allogeneic UC-MSCs. The well-documented systemic immune dysregulation in ASD—encompassing Th1/Th2 imbalance, Treg deficiency, altered cytokine profiles, and circulating autoantibodies [44]—suggests that AD-MSCs derived from affected patients themselves may carry intrinsic immunological perturbations that could attenuate their immunomodulatory potency, although direct comparative evidence of patient-derived versus healthy donor AD-MSC function in ASD remains lacking. Allogeneic MSC therapy, irrespective of tissue source, also carries the risk of anti-HLA antibody formation following infusion; in the only reported trial of allogeneic cord tissue-derived MSCs in children with ASD, five of twelve participants developed new class I anti-HLA antibodies that were clinically silent but were associated with a specific product lot or a partial donor–recipient HLA match, a consideration that could complicate the repeat dosing strategies commonly employed in ASD trials [22]. Clinical evidence for AD-MSC in ASD is currently the most limited among major MSC sources, comprising the only completed pediatric trial of allogeneic AD-MSC (NCT05602116, Cell2Cure^®^ C2C_ASC, Phase I, *n* = 10, ages 6–14 years, Denmark, 2023–2024)—other registered adipose-derived ASD trials having been either withdrawn without enrollment (NCT01502488) or based on autologous, multimodal protocols combining stromal vascular fraction, fecal microbiota transplantation, and dietary intervention, and still enrolling (NCT07546942)—together with a limited preclinical literature that includes amelioration of repetitive behavior, social deficit, and anxiety following intraventricular human AD-MSCs in a valproic acid (VPA)-induced mouse model [45]. The combination of harvesting constraints, source-related functional variability, theoretical autologous limitations, and sparse ASD-specific evidence collectively limits the suitability of AD-MSC as a primary candidate for pediatric ASD therapy.

### 3.3. UC-MSC Origin and Cellular Subpopulations

The umbilical cord is not anatomically homogeneous. It comprises several distinct compartments that yield subpopulations differing in phenotype and function. This section first describes the anatomy of the umbilical cord and the compartments from which MSCs are isolated, then characterizes WJ-MSCs as the predominant and most clinically utilized subtype, before considering the PV and SA populations and the implications of this compartmental heterogeneity for product consistency.

#### 3.3.1. Sourcing Anatomical Compartments of the Umbilical Cord

The human umbilical cord is a tubular structure that connects the developing fetus to the placenta. It begins to form around day 26 of gestation from the yolk sac and allantois and serves as the primary conduit for bidirectional fetal–maternal blood flow throughout pregnancy [26]. At term, the umbilical cord measures a mean length of approximately 55 cm with a mean diameter of 14.42 mm and an approximate weight of 40 g, and contains three umbilical vessels—two arteries carrying deoxygenated blood from the fetus to the placenta and one vein returning oxygenated blood to the fetus—encased in a gelatinous matrix known as WJ and surrounded by a single layer of amnion [51,52]. A distinctive anatomical feature of the umbilical vessels is the absence of a tunica adventitia; this peculiarity is functionally compensated by WJ itself, which provides mechanical support and prevents vessel compression and torsion during fetal movement, thereby preserving the continuous blood flow critical for fetal development [26].Beyond its mechanical role, the umbilical cord is now recognized as a heterogeneous reservoir of MSCs, with distinct subpopulations occupying four anatomically defined compartments: the amniotic membrane (AM), the sub-amnion (SA), the intervascular WJ, and the PV region surrounding the umbilical vessels [51,52] (Figure 1).

Isolation protocols differ across these compartments: WJ-derived cells are obtained by mechanically dissecting the gelatinous matrix after removal of the vessels using knife or forceps; PV cells are separated by looping the collected umbilical vessels and enzymatically digesting them; SA cells are recovered by dissecting the cord lining and cultivating the explants; and AM cells are isolated by enzymatic dissociation of the AM [52].Among these compartments, the WJ occupies the largest volume of the UC and yields the highest number of MSCs without prior culture-approximately 4.61 ± 0.57 × 10^6^ cells per cm of cord length, while the AM, SA, and PV compartments require enzymatic digestion or prolonged explant culture for adequate cell recovery [51]. Comparative immunophenotyping has identified CD24, CD108, and CD40 as discriminative markers distinguishing genuine MSCs from non-stem-cell mesenchymal contaminants [53]; WJ-derived populations show significantly elevated CD24^+^ and CD108^+^ expression, while both WJ- and PV-derived populations exhibit the lowest CD40^+^ contamination rate (approximately 26–27%) compared with SA, AM, or mixed-cord cultures (51–70% CD40^+^) [51]. The four compartments, while anatomically distinguishable, do not show sharp histological boundaries—particularly between SA and AM or between PV and WJ—and the persistent lack of consensus on umbilical cord anatomical nomenclature has resulted in potentially different cell populations being identified as arising from the same nominal source across studies [47].

A clinically critical anatomical distinction—central to the focus of this review—is that the umbilical cord tissue and the cord blood contained within the umbilical vessels represent two biologically distinct cellular sources. UCB is collected from the umbilical vein at delivery, whereas UC-MSCs reside in the surrounding stromal compartments (WJ, PV, SA, AM) and conform to the ISCT minimal criteria for MSCs: plastic adherence in standard culture, expression of CD73, CD90, and CD105, absence of CD45, CD34, CD14 (or CD11b), CD79α (or CD19), and HLA-DR surface markers, together with the capacity for trilineage differentiation into osteoblasts, adipocytes, and chondroblasts [33]. Immuno-phenotyping of UC-MSCs isolated from the various stromal compartments has further confirmed positive expression of CD29, CD44, CD73, CD90, CD105, and HLA-ABC together with absence of CD34, CD14, CD45, CD19, HLA-DR, and CD117 [52]. This anatomical and cellular separation forms the basis for fundamental differences in cellular composition, banking strategy, immunological behavior, and mechanism of therapeutic action between UCB-based and UC-MSC-based therapies in pediatric ASD; the specific cellular composition of UCB—including its dominant CD34^+^ hematopoietic stem cell and CD14^+^ monocyte populations—and the clinical implications of this distinction are detailed in Section 6.2.

#### 3.3.2. WJ-MSCs

Among the umbilical cord stromal compartments described above, WJ is the gelatinous connective tissue that constitutes the largest volume and serves both as the structural matrix supporting the umbilical vessels and as the principal anatomical reservoir of UC-MSCs (Section 3.3.1). Its extracellular matrix is a glycosaminoglycan (GAG)-rich ground substance in which hyaluronic acid predominates, with loosely arranged collagen fibers and a fibrillin-containing microfibrillar network embedded within it, and it is rich in growth factors including FGF-1 and 2, platelet-derived growth factor (PDGF), IGF-1, epidermal growth factor (EGF), and TGF; this microenvironment contributes to the comparatively high colony-forming unit-fibroblast (CFU-F) frequency observed during MSC isolation from the umbilical cord [26,52].

WJ stromal cells were formally characterized as an MSC population by Wang and colleagues, who demonstrated expression of CD44, CD105, CD29, and CD51 with absence of CD34 and CD45 together with adipogenic, osteogenic, and cardiomyogenic differentiation potential [54]; earlier work had shown that explant-derived WJ cells can be propagated for more than 80 population doublings and induced toward both neurons and glia in vitro, providing the experimental foundation for their neural lineage potential [55]. Comparative analyses against adult-tissue MSCs identify WJ cells as a primitive stromal population meeting all ISCT minimal criteria, with greater expansion capability and faster in vitro growth than BM-MSCs, good tolerance in allogeneic settings, and therapeutic effects mediated through trophic rescue and immune modulation rather than cell replacement [56]. WJ-MSCs constitutively express the pluripotency-associated factors octamer-binding transcription factor (OCT)-4, SOX2, and NANOG alongside CD105, CD73, and CD90 [26], have a doubling time of approximately 30–36 h, and although they may accumulate copy-number variations during long-term expansion they do not undergo malignant transformation or form tumors when injected into immunodeficient mice [52,57,58].

#### 3.3.3. PV- and SA-MSCs

The PV compartment of the umbilical cord harbors a second major MSC population, first defined by Sarugaser and colleagues as a nonhematopoietic (CD45^−^, CD34^−^) human umbilical cord perivascular (HUCPV) cell population expressing the canonical MSC markers CD105, SH3, CD90, and CD44 together with the pericyte-specific marker 3G5. HUCPV cells expand rapidly, with a CFU-F frequency of 1:333 and a doubling time falling from 60 h at passage 0 to 20 h at passage 2, and contain a subpopulation lacking both class I and class II major histocompatibility complexes (MHCs) that expands from approximately 20% at passage 0 to 95% after passage and cryopreservation, positioning them as an attractive source for allogeneic cell-based therapies [59].

The pericyte-like identity of PV-MSCs is supported by comparative analyses showing that MSCs and CD146-selected perivascular cells share morphology, immunophenotype, gene-expression profiles, and differentiation potential [60], by the positivity of both PV- and WJ-derived UC-MSCs for the pericyte markers CD146 and CD271 [52], and by in situ characterization confirming high yields of CD146^+^ MSCs with stable phenotype during long-term culture [61]. Histological cross-sections show no obvious demarcation between the PV and WJ regions and stromal cells are most abundant near the umbilical vessels [51], with the perivascular region of the WJ potentially containing up to 45% of all cord stromal cells [26]. Whether PV- and WJ-MSCs represent two distinct populations or a single PV lineage occupying overlapping anatomical zones remains debated [47].

The SA and AM compartments, collectively the cord lining, yield MSC populations that are clinically less favorable than those from the WJ and PV. Stromal cells are least abundant in the SA zone [26], derivation typically requires enzymatic dissociation and 10–14 days of explant culture, and the close proximity of the SA to the adjacent WJ and AM layers carries a risk of cross-contamination [51,52]. The cord lining also harbors a MUCIN1 (CD227)-expressing epithelial population co-expressing OCT-4, NANOG, stage-specific embryonic antigen (SSEA)-4, REX1, SOX2, and CD166, whose regenerative applications lie outside the scope of conventional MSC therapy [62].

Immunophenotypic characterization across compartments is inconsistent between studies: the PV phenotype has been reported as both positive and negative for CD14, CD106, and CD117, cord-lining populations as both positive and negative for CD34, CD45, and SOX2, and AM-, SA-, and mixed-cord populations show higher expression of CD140b and CD49d, indicating fibroblast cross-contamination during isolation [51]. These inconsistencies reflect technical heterogeneity in isolation protocols, donor variation, and the unresolved anatomical nomenclature discussed above (Section 3.3.1) [47]. Heterogeneity also extends below the compartment level, since counterflow centrifugal elutriation identifies small-sized UC-MSC subpopulations with higher proliferative capacity and reduced senescence than the bulk population [63].

Taken together, the comparative analysis of cells from the four UC compartments indicates a hierarchy of manufacturing suitability, no compartment having yet been compared with another in a clinical study. PV-MSCs share many advantageous properties with WJ-MSCs—including a similarly low non-stem-cell contamination rate (approximately 27% CD40^+^) and a pericyte-like immunophenotype [51]—but require enzymatic vessel-loop digestion for isolation, which adds technical complexity compared with the simpler mechanical extraction of WJ-MSCs [52]. SA- and AM-MSCs, in turn, are constrained by relatively low cell yield, time-consuming isolation, and substantial risk of fibroblast contamination, rendering them less suitable for rapid, large-scale, clinical-grade manufacturing. Consequently, WJ-MSCs remain the most practical UC-MSC source for clinical-grade manufacturing on current yield and contamination data, while HUCPV cells offer a viable alternative for indications requiring pericyte-specific properties or high MHC^−/−^ allogeneic populations. Importantly, regardless of compartment of origin, UC-MSCs uniformly satisfy the ISCT minimal criteria and share the core immunomodulatory and trophic properties that underlie their therapeutic potential-the molecular and cellular mechanisms of which are detailed in Section 3.4.

### 3.4. Immunomodulatory Properties of UC-MSC

The therapeutic rationale for UC-MSCs in pediatric ASD rests primarily on a multifaceted immunomodulatory capacity directed at the neuroinflammatory cascade described in Section 2. The principal effectors are not classical cytokines but a coordinated set of mediators-the lipid PGE2, the tryptophan-catabolizing enzyme indoleamine 2,3-dioxygenase (IDO), the growth factors TGF-β1 and HGF, the non-classical class I antigen HLA-G, and the cell-surface negative co-stimulatory regulator B7 homolog 1 (B7-H1)-operating through direct cell-to-cell contact and through soluble release to engage every major arm of the immune system and shift the overall tone from pro-inflammatory toward tolerogenic [19]. These pathways and their downstream amplification by IL-10 from immune-cell partners are treated in turn in the four sub-sections below: adaptive immune suppression mediated by PGE2, IDO, and B7-H1 (Section 3.4.1); Treg induction and HLA-G-mediated tolerogenic signaling (Section 3.4.2); innate immune reprogramming of natural killer (NK) cells and macrophages (Section 3.4.3); and the comparative immunomodulatory efficacy of UC-MSCs versus their bone-marrow counterparts (Section 3.4.4).

#### 3.4.1. Adaptive Immune Suppression: PGE2, IDO, and B7-H1 Axes

PGE2 functions as a central effector of UC-MSC immunomodulation. Co-culture of MSCs with allogeneic immune cells lowers pro-inflammatory cytokine output from dendritic cells, Th 1 cells, and NK cells while expanding Treg populations and raising IL-10 from type-2 dendritic cells; rising PGE2 accompanies these shifts, and cyclooxygenase inhibition abolishes the entire phenotype [19]. The PGE2 axis is preserved across MSC sources of distinct ontology, with BM, AD, and WJ-derived cells all constitutively secreting PGE2 at basal amounts of roughly 1500–2000 pg/mL and further upregulating secretion during T-cell co-culture; cyclooxygenase blockade reduces WJ-MSC suppressive activity by more than two-thirds, establishing PGE2 as a non-redundant effector that UC-MSCs share with their adult counterparts [64].

A complementary pathway operates through IDO-mediated tryptophan catabolism, which is not constitutive but inducible. IDO transcription and enzymatic activity in MSCs are licensed by interferon-gamma (IFN-γ) released from the very lymphocytes targeted for suppression, generating a feedback loop in which T-cell and NK-cell activation initiates the suppressive machinery; tryptophan depletion and kynurenine accumulation then account for the inhibition of CD4^+^ and CD8^+^ T-cell proliferation, since exogenous tryptophan rescues proliferation and IDO blockade restores effector function [31,65]. UC-MSCs recapitulate this pathway with full fidelity: baseline IDO transcripts are absent, IFN-γ priming drives IDO protein expression to >95% positivity within three days, and 1-methyl tryptophan abrogates UC-MSC suppression of CD4^+^ T-cell proliferation. A complementary surface-bound pathway reinforces the same outcome, with the negative co-stimulatory molecule B7-H1 constitutively expressed at moderate levels (~35–42% positivity) and similarly upregulated under IFN-γ; only simultaneous blockade of B7-H1 and IDO restores T-cell proliferation toward control levels, identifying the two as parallel non-redundant pathways rather than functionally redundant ones [66].

#### 3.4.2. Treg Induction and HLA-G Tolerogenic Signaling

Beyond direct cytokine suppression, MSCs drive Treg differentiation and tolerogenic surface signaling through coordinated soluble and membrane-bound pathways; the soluble and Treg-inducing arms of this axis have been defined principally in BM-MSCs, whereas the HLA-G component has been documented directly in UC-derived cells.

TGF-β1 and HGF act through Transwell-permeable diffusion to suppress allogeneic CD4^+^ and CD8^+^ T-cell proliferation, with neutralizing-antibody experiments confirming that direct contact is dispensable for the soluble component [32]. Beyond direct suppression, TGF-β1 cooperates with PGE2 in non-redundant fashion to drive Treg differentiation, with forkhead box P3 (FoxP3)^+^CD25^+^ regulatory phenotypes emerging only when both mediators are intact and the resulting Tregs themselves expressing TGF-β1 to propagate the regulatory signal [67]. A parallel HLA-G axis further consolidates the UC-MSC tolerogenic signature: umbilical cord matrix stromal cells express pan-HLA-G messenger RNA (mRNA) while lacking CD40, CD80, and CD86, a profile that supports both their hypo-immunogenicity to host T cells and their suppression of allogeneic lymphocyte responses in mixed lymphocyte reactions [68]. The soluble HLA-G5 isoform, secreted by MSCs in an IL-10-dependent manner, then closes a positive-feedback loop-suppressing T-cell proliferation and driving subsequent expansion of CD4^+^CD25^high^ FoxP3^+^ Tregs whose IL-10 production in turn supports the original tolerogenic signal [69].

#### 3.4.3. Innate Immune Reprogramming: NK Cells and Macrophages

MSC modulation of innate immune effectors is particularly relevant to the chronic, microglia- and monocyte-driven inflammation that characterizes ASD; the evidence in this subsection derives from BM-MSC and unspecified-source MSC studies and has not yet been reproduced in UC-derived cells. NK cell proliferation, cytotoxicity, and cytokine production are blunted by the combined IDO and PGE2 activity already described, with neither molecule alone sufficient to recapitulate the full effect [70]. Tissue macrophages undergo a more sustained phenotypic conversion, adopting a CD206^high^, IL-10^high^, IL-12^low^, TNF-α^low^ profile with enhanced phagocytic activity that defines a distinct alternatively activated subtype committed to tissue repair and resolution rather than to acute defense [71]. The systemic relevance of this macrophage reprogramming was demonstrated in a polymicrobial sepsis model in which MSC-derived PGE2 engaged PGE2 receptor subtype 2 (EP2) and 4 (EP4) receptors on host macrophages to induce IL-10, and depletion of either macrophages or IL-10 signaling abolished the survival benefit of MSC infusion—placing the PGE2–macrophage–IL-10 axis at the apex of in vivo MSC immunomodulation [72].

#### 3.4.4. UC-MSC vs. BM-MSC: Comparative Immunomodulatory Efficacy

The combined operation of these four axes renders UC-MSC immunomodulatory activity at least comparable to that of BM-derived counterparts. In direct side-by-side comparison, extra-embryonic sources (amnion, placenta, WJ, umbilical cord) suppress mixed lymphocyte reactions to a degree statistically indistinguishable from BM-MSCs, while showing substantially greater proliferative longevity—reaching replicative senescence at passage 18–22 rather than passage 10—a difference of central importance for the manufacturing scale-up addressed in Section 4 [73]. Reported source rankings vary with assay, readout, and culture protocol: whereas some comparisons assign the highest immunomodulatory activity to BM-MSCs (Section 3.2.2), a study comparing all three sources under identical conditions found CD4^+^ and CD8^+^ T-cell subsets to be inhibited comparably by MSCs from BM, WJ, and AD, with WJ- and AD-derived cells somewhat more efficient than BM-MSCs and AD-MSCs showing the largest effect on both proliferation and dose-dependent downregulation of the activation marker CD38 [64]. On balance, UC-MSCs are at least comparable to BM-MSCs in functional immunosuppression in mixed lymphocyte reaction assays, although the reported ranking is assay dependent, and the four axes detailed in Section 3.4.1, Section 3.4.2 and Section 3.4.3 together deliver a coordinated dampening of the inflammatory tone underlying pediatric ASD. The complementary neurotrophic and angiogenic capacities by which UC-MSCs additionally support neurodevelopmental recovery are addressed in Section 3.5.

### 3.5. Neurotrophic and Angiogenic Properties of UC-MSC

Beyond their immunomodulatory capacity, UC-MSCs constitute a second therapeutic axis in pediatric ASD through the constitutive expression and release of neurotrophic and angiogenic factors supporting neuronal survival, synaptic plasticity, and vascular development. This trophic profile is mechanistically distinct from the contact- and mediator-dependent immunomodulation of Section 3.4 and engages a developmental dimension of ASD pathophysiology that immunological intervention alone cannot reach. The principal effectors include BDNF, NGF, glial cell line-derived neurotrophic factor (GDNF), and neurotrophin-3 (NT-3) on the neurotrophic side, and VEGF, EGF, and the VEGF receptor Fms-related tyrosine kinase 1 (FLT1) on the angiogenic side. In vitro and in vivo evidence indicate that this combined trophic profile distinguishes UC-MSCs from BM-derived counterparts in direct laboratory comparisons, as already documented for immune modulation; no clinical comparison of the two sources has been reported.

At the transcriptional level, WJ-derived MSCs constitutively overexpress genes involved in neurotrophic support relative to BM-MSCs and to multipotent adult progenitor cells (MAPC). Gene ontology analysis identifies enrichment of pathways related to cell adhesion, proliferation, and immune-system functioning in WJ-MSC, and functional readouts confirm a parallel biological consequence: conditioned medium harvested from WJ-MSC induces neuronal maturation of SH-SY5Y neuroblastoma cells to a greater extent than conditioned medium from BM-MSC, while in vivo transplantation produces immune modulation on T cells comparable to that of BM-MSC and MAPC-indicating that the WJ source adds neurotrophic capacity without losing immunological function [74].

The angiogenic side of this signature has been mapped at similar transcriptional resolution. Direct gene-expression profiling of human WJ-MSC and BM-MSC reveals that WJ-MSC preferentially express angiogenesis- and growth-related genes including EGF and the VEGF receptor FLT1, with enrichment in canonical pathways centered on VEGF and phosphoinositide 3-kinase (PI3K)-nuclear factor kappa B (NF-κB); BM-MSC instead favor osteogenic and antigen-presentation programs and are correspondingly more efficient in osteogenic and adipogenic differentiation. WJ-MSC proliferate more robustly and share a larger fraction of their molecular signature with embryonic stem cells, placing them closer to a primitive, growth- and angiogenesis-oriented phenotype well suited to neurovascular support rather than to skeletal remodeling [75].

Three in vivo systems demonstrate that these transcriptional differences translate into measurable functional recovery. In a hemiparkinsonian rat model, undifferentiated human UC-MSC transplanted into brain without immune suppression ameliorated apomorphine-induced rotations and were interpreted as therapeutically relevant to neurodegenerative disease through their constitutive expression of growth and angiogenic factors, while parallel transplantation into normal rats produced neither brain tumors nor frank host immune rejection [76]. In a peripheral-nerve transection model in BALB/c mice, topical application of hWJ-MSC onto a sutured sciatic nerve produced dose-dependent functional recovery surpassing that of adipocyte-derived stem cell controls, with histological evidence of improved myelination at one year, and the applied cells expressed higher NT-3, BDNF, and GDNF than the adipocyte-derived comparator-linking the WJ-MSC neurotrophic profile directly to regenerative outcome [77]. A third system integrates both axes within one model: in a diabetic foot ulceration rat, intra-arterial infusion of human cord blood-derived MSCs raised serum NGF, increased capillary density in nerve-innervated gastrocnemius muscle, and restored femoral nerve conduction velocity [78], coupling the angiogenic transcriptional profile to a functional vascular outcome alongside neurotrophic recovery.

The clinical relevance of this trophic profile to pediatric ASD rests on independent evidence that the neurotrophic milieu is itself dysregulated in affected children. Meta-analysis of 19 studies comprising 2896 participants found significantly elevated peripheral BDNF in children with ASD compared with healthy controls (Hedges g, 0.49), with the effect concentrated in the non-neonatal pediatric subgroup rather than in neonates later diagnosed with ASD [79]. An independent meta-analysis of 14 studies comprising 2707 participants reached the same conclusion at a standardized mean difference (SMD) of 0.63 and identified the effect as specific to childhood rather than to adulthood, supporting peripheral BDNF as a candidate biomarker of an abnormal neurotrophic profile in pediatric ASD [80]. The dysregulation is one of elevation rather than deficiency and is documented in peripheral blood rather than the CNS, cautioning against framing UC-MSC neurotrophic secretion as a simple replacement therapy. A more defensible interpretation is that UC-MSC-derived BDNF, NGF, GDNF, and NT-3 act through paracrine modulation at sites of inflammation and neurovascular injury rather than through systemic supplementation, rebalancing rather than replacing the trophic environment. The donor- and process-related heterogeneity that determines whether this potential is consistently realized in clinical-grade UC-MSC products is addressed in Section 3.6.

### 3.6. Heterogeneity and Limitations of UC-MSC Products

The biological properties detailed in Section 3.3, Section 3.4 and Section 3.5 describe an idealized UC-MSC product, but clinical translation has been complicated by extensive heterogeneity in commercially available and academic-grade preparations. The principal sources of inter-batch variability are the donor (maternal age, gestational age, neonatal birth weight, umbilical-cord width), the tissue-isolation method (explant vs. enzymatic digestion), the in vitro expansion conditions (passage number and culture medium), the cryopreservation and post-thaw recovery process, and the absence of universally accepted potency-release assays. These sources operate cumulatively rather than independently, so that the same donor cord harvested by different isolation protocols, expanded under different culture conditions, and cryopreserved by different operators can yield final cell products with measurably distinct immunomodulatory and neurotrophic potency. The four sub-sections below treat these sources in turn: donor and isolation variability (Section 3.6.1); passage and culture conditions during expansion (Section 3.6.2); cryopreservation and post-thaw recovery (Section 3.6.3); and the potency-assay framework proposed by the International Society for Cell and Gene Therapy (Section 3.6.4).

#### 3.6.1. Donor and Isolation Variability

At the donor level, both maternal and neonatal characteristics influence WJ-MSC yield in a reproducible manner. Analysis of 79 umbilical-cord samples identified maternal age as negatively correlated with WJ-MSC yield and birth weight and gestational age as positively correlated, while parity, neonatal sex, fetal presentation, and head circumference showed no significant association; umbilical-cord width itself was inversely correlated with passage-1 doubling time, indicating that even gross cord morphology is informative for predicting downstream proliferative behavior [81]. The tissue-isolation method introduces a second layer of variability that operates independently of donor selection: head-to-head comparison of explant culturing of WJ versus collagenase tissue digestion shows that explant-isolated WJ-MSC display superior recovery of mesenchymal-like cells with increased expansion potential, establishing the isolation method as a determinant of both early cell yield and downstream proliferative reserve [74].

#### 3.6.2. Expansion-Phase Variability: Passage and Culture Conditions

In vitro expansion introduces variability through both passage number and culture-medium composition. BM-MSCs show measurable senescence within typical clinical-expansion windows: population doublings per passage drop from approximately 7.7 at early passage to 1.2 by the tenth passage, with parallel telomere shortening and loss of differentiation potential beyond passage 6 [82]. The proliferative longevity advantage of extra-embryonic sources documented in Section 3.4—WJ- and UC-MSC reaching replicative senescence near passage 18–22 rather than passage 10 [73]—therefore constitutes a meaningful manufacturing margin only when matched with culture conditions that do not themselves accelerate senescence. The choice of serum supplement is the most consequential of these conditions: head-to-head comparison of WJ-MSC expanded in xeno-free, serum-free medium versus standard fetal bovine serum (FBS)-containing medium shows that the xeno-free condition maintains MSC identity and immunosuppressive potential while delivering superior growth kinetics, preserved genomic stability across large-scale expansion, and a secretome with functional angiogenic activity [83]. The shift away from animal serum thus eliminates both a documented source of inter-batch variability and a regulatory hazard related to xenogeneic contamination.

#### 3.6.3. Cryopreservation and Post-Thaw Recovery

Cryopreservation, the final pre-administration step in UC-MSC manufacturing, carries its own functional consequences. Direct comparison of fresh, freshly thawed, and 24-h acclimated BM-MSCs—a cryopreservation effect not specific to cell source—shows that immediate post-thaw cells maintain multipotent differentiation and basic immunomodulatory function but exhibit reduced surface-marker expression (CD44, CD105), elevated apoptosis, decreased proliferation and clonogenic capacity, and a transient downregulation of regenerative and anti-inflammatory gene expression; a 24-h acclimation period after thaw is sufficient to restore most of these parameters, with thawed-and-acclimated cells demonstrating significantly stronger T-cell suppression than freshly thawed cells and IFN-γ secretion recovering during the same window [84]. The practical consequence is that potency measurements made on freshly thawed cells may systematically underestimate the activity of the same product after a brief post-thaw recovery, with implications for both release criteria and the design of patient-administration protocols.

#### 3.6.4. Potency Assays and the ISCT Standardization Framework

The cumulative variability introduced by donor, isolation, expansion, and cryopreservation can only be managed through quantitative release assays that index functional potency. The ISCT minimal criteria for MSC identification—plastic adherence, a defined surface-marker profile, and tri-lineage differentiation to osteoblasts, adipocytes, and chondrocytes [33]—were developed to harmonize reporting across laboratories and remain useful as identity criteria, but they do not assess therapeutic potency and have proven insufficient as release criteria for advanced clinical trials. The ISCT subsequently issued a consensus framework recommending that immune-functional release assays be deployed as a matrix combining three complementary readouts: quantitative RNA analysis of selected gene products, flow-cytometric analysis of functionally relevant surface markers, and protein-based assays of the MSC secretome; the framework further proposes that these assays be made publicly accessible through open publication or database deposition to enable cross-laboratory comparability [85]. UC-MSC products that pass identity criteria can therefore still differ substantially in immunomodulatory function, and a clinically meaningful comparison between donors, batches, or commercial preparations requires the matrix-assay framework rather than the minimal-criteria checklist alone.

The convergence of these five sources of variability—donor characteristics, isolation method, passage and culture conditions, cryopreservation, and the absence of universally adopted potency-release assays—explains why nominally identical UC-MSC products from different manufacturers can produce divergent clinical results in pediatric ASD trials despite shared biological rationale. The manufacturing-oriented response to these challenges—standardized cord-tissue procurement criteria, validated explant-based isolation, xeno-free expansion protocols with defined passage limits, controlled cryopreservation with post-thaw acclimation, and matrix potency assays applied as release criteria—is the subject of Section 4. The preclinical and clinical evidence in Section 5 and Section 6 should be interpreted with these sources of heterogeneity in mind, since differences in trial outcomes may reflect product-level variability as much as biological or clinical heterogeneity in the patient population.

## 4. Manufacturing and Quality Considerations for Clinical-Grade UC-MSC

Translating the biological potential of UC-MSCs into a reproducible clinical product depends on controlling each step of the production chain at which the variability sources of Section 3.6 are introduced. The subsections that follow trace this chain in sequence, treating each stage as a point at which an otherwise unmeasured source of inter-batch variability becomes a defined, documented, and release-tested input.

### 4.1. Procurement and Donor Screening

The procurement of umbilical cord tissue for clinical-grade UC-MSC manufacturing is governed by transnational regulatory frameworks for tissue donation. In the European Union, Directive 2004/23/EC sets the standards for donation, procurement, testing, processing, and storage of human tissues and cells; national agencies such as the French Biomedicine Agency implement this framework through accredited cord blood and tissue banks affiliated with maternities, where fully informed, written maternal donor consent is obtained [86]. Within this regulatory architecture, donor validation is recognized as one of the primary pillars of safety in clinical-grade MSC production, alongside choice of starting material, process design, and in-process controls [87]. Maternal serological screening covers a broad panel of transmissible infections—hepatitis B surface and core antibodies (anti-HBs and anti-HBc), hepatitis C virus (HCV), human immunodeficiency virus (HIV) I/II, syphilis, Chagas disease, human T-cell lymphotropic virus (HTLV) I/II, and nucleic acid testing (NAT) for HCV), HIV, and hepatitis B virus (HBV)—and cord blood is independently screened against the same panel as a second layer of donor-suitability verification, with non-compliant donations rejected before isolation begins [88].

Cord acceptance criteria for the WJ-MSC derivation process were systematically optimized through analysis of factors affecting derivation success, with the finding that efficient UC-MSC derivation is achieved using cord fragments larger than 7 g processed within 80 h from birth [89]. The same systematic analysis demonstrated that even small and relatively aged cord fragments—which would otherwise be discarded from public cord blood banking programs—yield UC-MSCs of consistent identity and expansion potential when the 7 g weight and 80 h time thresholds are met, supporting the recovery of clinical-grade starting material from a wider donor pool [89]. The donor-level variables documented at procurement intersect with the predictive findings discussed in Section 3.6.1—maternal age, gestational age, neonatal birth weight, and umbilical-cord width—so that manufacturing records capture both the regulatory requirements for donor suitability and the biological parameters that influence downstream cell yield. Beyond infectious-disease exclusion, gender, age, disease severity, co-morbidities, and other donor-level factors that influence MSC biological properties are taken into consideration during donor selection and recorded for downstream traceability when batch-level variation is later observed [90].

Quality risk management integrates the procurement step with the manufacturing chain that follows. Clinical-grade WJ-MSC production has implemented procurement controls in accordance with International Council for Harmonisation (ICH) Q9 guidelines on quality risk management and World Health Organization (WHO) Annex 2 guidelines on good manufacturing practice (GMP) for pharmaceutical products, with failure mode and effects analysis (FMEA) applied to identify procurement-related risks that could compromise critical quality attributes of the final cell product [88]. This same control architecture also covers microbiological safety and the in-process testing designed to prevent transformation, senescence, and decrease in immunoregulatory and differentiation function during downstream manufacturing [87]. UC-MSC products derived from such procurement workflows have received clinical-trial authorization as advanced therapy medicinal products (ATMPs) under the European regulatory framework governing somatic-cell medicinal products [86,90]. One such application is NCT04333368, a French UC-MSC ATMP clinical trial in severe acute respiratory syndrome coronavirus 2 (SARS-CoV-2)-induced acute respiratory distress syndrome (ARDS) [86]. This integration converts donor- and cord-level heterogeneity from an unmeasured source of inter-batch variability into a controlled, documented input that supports regulatory acceptance of UC-MSC for clinical use.

### 4.2. Isolation and Initial Characterization

The standard isolation procedure for clinical-grade UC-MSC manufacturing in European UC-MSC ATMPs that have received clinical-trial authorization is the explant method [86,88], a choice supported by head-to-head comparisons demonstrating superior recovery of mesenchymal-like cells with increased expansion potential from WJ explants relative to collagenase digestion [74]. Subsequent work using GMP-compatible serum-free medium has shown that explant-derived and collagenase-derived UC-MSCs display largely equivalent gene-expression profiles and that both isolation methods are compatible with downstream scale-up, with enzyme-based isolation paired with bioreactor expansion proposed as particularly suitable for industrialized mass production [91]. The choice between methods at the clinical-grade manufacturing level therefore reflects both regulatory considerations and operational scale, since enzymatic digestion paired with bioreactors becomes more attractive at industrial scale.

In the explant-based GMP workflow, the umbilical cord is washed and dissected longitudinally at the manufacturing facility; the two umbilical arteries and the single umbilical vein are removed mechanically before the underlying WJ is scraped from the inner surface with a surgical scalpel and seeded into culture vessels containing GMP-compatible derivation medium, where culture-medium exchange every 3–4 days supports the migration of WJ-derived cells to passage 0 at 70–90% confluence within approximately two to three weeks [88,89]. Subsequent controlled expansion at defined seeding densities through passage 1–3 generates the cell numbers required for downstream banking; doubling time and population doublings remain stable through passage 3–4 in validated bioprocesses, with senescent morphology and reduced proliferative capacity emerging at later passages and prompting most clinical-grade protocols to limit expansion to these early passages, at which identity and functional attributes remain stable [86,89].

In-process characterization at this stage applies the ISCT minimal criteria for MSC identity [33,37]: plastic adherence, tri-lineage differentiation potential, and a defined surface-marker profile requiring ≥95% positivity for CD73, CD90, and CD105 and ≤2% expression of CD45, CD34, CD14 or CD11b, CD79α or CD19, and HLA-DR. WJ-MSC produced under GMP-compliant explant protocols routinely satisfy these criteria at the master cell bank (MCB) stage, with reported phenotypic values of ≥95% for the positive markers (CD73, CD90, CD105) and ≤2% for HLA-DR [86,88]. Tri-lineage differentiation into adipogenic, chondrogenic, and osteogenic lineages confirms multi-potentiality at the initial characterization stage in clinical-grade UC-MSC preparations [89].

### 4.3. Large-Scale Expansion in GMP Conditions

Scaled-up production of clinical-grade UC-MSCs builds on three complementary process levers: defined culture supplements that replace animal-derived serum, volumetric bioreactor platforms that decouple cell yield from culture-vessel surface area, and pre-administration priming strategies that enhance functional potency before release. The first two address the upstream supply problem of generating clinically relevant cell numbers under reproducible conditions, while the third addresses the downstream potency question of whether expanded cells exit the bioprocess in their most therapeutically active state. The subsections below treat each lever in turn.

#### 4.3.1. Serum and Medium Alternatives

The historical reliance on FBS as a culture supplement raises distinct safety and regulatory concerns for clinical-grade MSC production-animal-derived components introduce lot-to-lot variability, potential xenogeneic immune responses upon administration, and theoretical risks of zoonotic agent transmission—prompting an extensive search for FBS-free alternatives compliant with GMP guidelines, broadly classified into human-derived blood products such as human platelet lysate (HPL), human serum, or UCB serum, and fully chemically defined xeno- and serum-free media [92].

Among human-derived alternatives, pooled human platelet lysate (pHPL) emerged as a foundational FBS replacement in a landmark study showing that small bone-marrow aspirate volumes could yield clinically relevant MSC numbers within a single primary culture with retention of identity, immunophenotype, differentiation potential, and genomic stability [93], and subsequent work has refined preparation methodology while confirming comparable stem-cell marker maintenance, CFU-F frequency, and population doublings across HPL variants [94]. A state-of-the-art workshop on HPL nonetheless concluded that its manufacturing remains incompletely standardized with respect to platelet source, preparation methodology, and pathogen inactivation, and that consensus minimum release criteria are required to limit performance variability across cell-therapy products [95].

Complete xeno-free, serum-free conditions for WJ-MSC isolation and clinical-scale expansion have been established with commercial GMP-compatible media (Mesencult XF/SF), eliminating animal-derived components from the entire workflow including cryopreservation; WJ-MSCs cultured in these conditions maintained superior growth kinetics relative to FBS controls while preserving functional angiogenic potency in Matrigel-based assays [83]. An in-house GMP-compatible serum-free medium has been independently developed and shown to support both explant- and collagenase-based UC-MSC isolation as well as both static and microcarrier-based bioreactor expansion, with microarray analyses confirming that gene-expression profiles remained comparable regardless of isolation or expansion route taken—a level of flexibility that supports serum-free systems as the chemically defined backbone for downstream scale-up [91]. Although fully chemically defined formulations remain the ultimate standardization goal and their development continues [92], these GMP-compatible platforms—HPL-based or chemically defined—now provide the supplementation backbone on which scaled-up expansion in bioreactor systems can be built.

#### 4.3.2. Bioreactor Scale-Up

Clinical-grade MSC production for ATMP manufacturing typically begins in two-dimensional planar systems such as multi-layer cell factories [86], a format that is well suited to small to moderate batch sizes but rate-limited for large-scale allogeneic banking. As the demand for clinically relevant cell doses has grown, the field has shifted toward three-dimensional (3D) bioreactor systems—stirred-tank bioreactors (STRs) coupled with microcarriers and other volumetric platforms—that decouple cell yield from culture surface and offer process-controlled environments suitable for ATMP manufacturing [96]. A comparability exercise using a 3-L single-use STR with microcarriers demonstrated that WJ-MSCs expanded in 3D met the same critical quality attributes and product specifications as cells from established 2D bioprocesses, with comparable cell yields (3D: 9.48 × 10^8^ ± 1.07 × 10^7^ cells in 6 days vs. 2D: 9.73 × 10^8^ ± 2.36 × 10^8^ cells in 8 days), >90% viability, retention of MSC immunophenotype (CD90^+^/CD73^+^/CD31^−^/CD45^−^/HLA-DR^−^), normal karyotype, and strong in vitro immunomodulatory potential; CD105 detection was lower in 3D harvests than in 2D, which the authors attributed to the high-concentration trypsin used for cell retrieval from microcarriers and reported as not affecting multipotency [96]. Subsequent process-development work in 0.25-L STR systems identified attachment efficiency and microcarrier colonization as critical process parameters governing fold expansion, with up to 1.47 × 10^9^ WJ-MSCs obtained in a real-scale 2.4-L batch while preserving identity, viability, and differentiation potency [97]. Comparable equivalence has been reported for UC-MSCs expanded in static versus microcarrier-based stirred-tank culture in GMP-compatible serum-free medium, although bioreactor adaptation altered the fatty-acid and phospholipid composition of the cell membrane in response to shear stress-an observation highlighting the subtle bioreactor-specific cellular changes that may warrant careful potency monitoring during process transfer [91]. Closed-system automated platforms represent a further step toward GMP standardization by eliminating open manipulations during the expansion phase. In an automated hollow-fiber system, the bioreactor product was phenotypically and functionally similar to manually expanded BM-MSCs while requiring significantly less time, effort and manufacturing cost, although 11 of 42 cytokines analyzed were differentially expressed between the two methods, indicating that performance may differ for some clinical applications [98]. Independent of the specific 3D platform chosen, the cumulative evidence indicates that GMP-compliant scaled-up MSC expansion can reproducibly yield clinically relevant cell numbers without compromising regulatory release criteria, providing the upstream supply on which functional optimization strategies—the focus of the next sub-section—can build.

#### 4.3.3. Pre-Administration Priming

Beyond standardized culture systems, pre-administration priming has emerged as a strategy to actively enhance MSC potency rather than merely preserve baseline function during expansion. Three principal priming strategies have been systematically compared in side-by-side functional assays—pro-inflammatory cytokine licensing, hypoxic preconditioning, and 3D spheroid culture—each producing profound transcriptomic adaptations and engaging distinct immunosuppressive mechanisms, with pro-inflammatory cytokine licensing and continuous 3D culture priming yielding the strongest enhancements in T-cell suppressive capacity; priming effects may rapidly fade once cells return to standard culture conditions, an observation with direct implications for the timing of administration after thawing or product release [99].

Cytokine licensing typically uses IFN-γ, TNF-α, or their combination at concentrations of approximately 10 ng/mL applied for ~24 h [100]. In WJ-MSCs, short-term TNF-α/IFN-γ priming reprogrammed the transcriptome to upregulate pathways linked to immune response and cytokine signaling, with metabolomic analyses revealing enhanced kynurenine pathway activity, a molecular signature of IDO-mediated tryptophan catabolism, although at the expense of reduced osteogenic and adipogenic differentiation potential [31,100]. The functional consequences extend in vivo: WJ-MSCs primed with IFN-γ or the Toll-like receptor (TLR) 3 agonist polyinosinic-polycytidylic acid (poly I:C) showed enhanced therapeutic effects relative to non-primed cells in a murine atopic dermatitis model, with reduced epidermal thickness, lower IL-17 in skin-draining lymph nodes, and pathway-specific transcriptomic enrichment [101]. Blockade of IDO with epacadostat abolishes the ability of IFN-γ-primed AD-MSCs to suppress human T-cell proliferation, identifying the IDO/kynurenine axis as the principal mediator of cytokine-induced immunosuppression [102], and IFN-γ priming induces IDO activity as part of the standard potency assay in clinical-grade ATMP development [86].

Hypoxic preconditioning exploits the physiologic oxygen gradient of the native MSC niche (1–5% O_2_). Short-term exposure of BM-MSCs to 2% O_2_ produced the strongest functional gains, including increased proliferation, upregulated VEGF expression, and elevated IL-1 receptor antagonist (IL-1Ra) secretion, whereas long-term hypoxia impaired surface marker expression and reduced expansion yields [103]; hypoxic priming also enhances paracrine VEGF and HGF secretion, with VEGF knockdown abolishing the anti-fibrotic effect in a renal ischemia–reperfusion model [104]. The immunological consequences of hypoxia priming are distinct from those of cytokine priming: although both strategies upregulate IDO, only hypoxic priming has been shown to expand Tregs in co-culture, indicating non-overlapping immunosuppressive mechanisms across priming modalities [105]. Beyond oxygen-tension manipulation, 3D spheroid priming represents a complementary modality whose immunosuppressive effects, unlike those of cytokine licensing, are maintained continuously during 3D culture rather than fading after return to standard conditions-a property that may be exploited for sustained-release MSC formulations [99].

For pediatric ASD, cytokine priming most directly enhances the IDO-mediated axis of Section 3.4 and hypoxic priming the neurotrophic and angiogenic secretome of Section 3.5. Whichever is selected, priming-induced potency may dissipate during storage and transport, an issue that intersects with the cryopreservation and banking considerations addressed in the following section.

### 4.4. Banking, Quality Control, and Release Criteria

The transition from research-grade MSC cultures to clinically deliverable ATMP require the establishment of a structured banking system together with validated quality-control assays and predefined release criteria. Published GMP-compliant UC-MSC ATMP frameworks use a two-tier banking strategy: a MCB is generated at early passage from each qualified umbilical cord, and working cell banks (WCBs) are subsequently derived before reaching the senescence-onset passage for clinical lot production, with batch-specific quality testing applied at both bank levels and at the final product stage [86]. The number of freezing–thawing cycles imposed by this banking architecture matters: BM-MSC platforms have shown that one to two freezing steps with at least one intervening passage do not substantially alter manufacturing parameters or quality attributes, whereas four or more freezing steps can induce earlier senescence, an outcome that has direct implications for bank design and product lifetime [106]. The current Good Manufacturing Practice (cGMP) framework that governs these decisions—covering facility qualification, traceability, environmental monitoring, and validated documentation—has been comprehensively reviewed in the ATMP context, as a set of cGMP considerations spanning the MSC manufacturing process [90].

Cryopreservation remains the standard method for stabilizing MSC products between manufacture and patient administration, but it carries trade-offs that have shaped current practice. Conventional cryoprotectant solutions rely on 5–10% dimethyl sulfoxide (DMSO), which permeates cells efficiently and protects against ice-crystal damage but is recognized as potentially toxic to both patients and MSCs [107]. An international multicenter Production Assistance for Cellular Therapies (PACT)/Biomedical Excellence for Safer Transfusion (BEST) collaborative study comparing a DMSO-free solution containing sucrose, glycerol, and isoleucine in a Plasmalyte A base against in-house DMSO solutions reported pre-cryopreservation viability of 94.3%, with the DMSO-free solution yielding 92.9% viable cell recovery post-thaw alongside comparable immunophenotype (CD45^−^/CD73^+^/CD90^+^/CD105^+^) and global gene expression profiles—values that remained clinically acceptable above the 80% viability threshold [107]. For UC-MSCs specifically, a DMSO-free protocol using zwitterionic betaine delivered intracellularly by electroporation has been shown to maintain normal viability and function with reduced reactive oxygen species and in vivo distribution comparable to fresh cells, providing a UC-MSC-tailored alternative to the DMSO-based standard [108].

Equally important to the choice of cryoprotectant is what happens to the cell product after thawing. Freshly thawed MSCs delivered immediately to patients exhibit measurable functional impairment relative to fresh-from-culture cells: in BM-MSCs, FT preparations showed reduced CD44 and CD105 surface expression, increased apoptosis, decreased proliferation and clonogenic capacity, and diminished IFN-γ secretion in response to T-cell co-culture [84]. A 24-h acclimation period after thawing reversed several of these deficits-apoptosis was significantly reduced, angiogenic and anti-inflammatory genes were upregulated, and the cells regained T-cell suppressive potency superior to that of immediately thawed cells—providing direct evidence that the timing between thaw and infusion is itself a critical process parameter [84]. Consistent with this, an independent clinical-grade BM-MSC banking study reported that freshly thawed cells exhibited approximately 50% reduction in T-cell immunosuppression assay performance compared with continuously cultured counterparts, while retaining phenotype and tri-lineage differentiation potential-an observation supporting either post-thaw acclimation or potency-corrected release specifications when using cryopreserved products [106].

Release criteria for UC-MSC ATMPs combine identity, purity, viability, microbiological safety, and potency testing. Identity is established by flow cytometric confirmation of the ISCT minimal criteria, requiring ≥95% positivity for CD73, CD90, and CD105 and ≤2% expression of CD45, CD34, CD14 or CD11b, CD79α or CD19, and HLA-DR [33]. In published GMP-compliant WJ-MSC bioprocesses, MCBs and WCBs routinely report ≥95% positivity for the positive markers (CD73, CD90, CD105) and HLA-DR expression below the ISCT threshold, while microbiological release testing covers sterility, mycoplasma, endotoxin (with defined thresholds based on pharmacopeial standards), and adventitious viral contamination, supplemented by in-process testing during expansion [86,88]. Microbiological release testing covers sterility, mycoplasma, endotoxin (with defined thresholds based on pharmacopeial standards), and adventitious viral contamination, supplemented by in-process testing during expansion [87]. Potency assay design remains the most challenging element of release testing because no single in vitro assay fully captures the multimodal mechanisms of MSC action; the field has therefore moved toward function-specific assays calibrated to the intended clinical indication-for immunomodulation-driven applications such as ASD, T-cell suppression assays and IDO induction following IFN-γ priming are commonly used [88,109]. Donor-to-donor and lot-to-lot heterogeneity in immunomodulatory potency is widely acknowledged and remains the principal unresolved challenge in MSC product standardization, motivating ongoing development of multi-parameter potency matrices, clonal selection, and donor pre-qualification strategies [110]. Collectively, the banking, cryopreservation, and release-testing framework outlined here ensures that UC-MSC products entering pediatric ASD trials meet identity, safety, and potency specifications appropriate to a vulnerable patient population, while leaving meaningful room for further harmonization of potency assays across manufacturing centers.

Translating these specifications into pediatric ASD trial design has two direct implications. First, the post-thaw functional impairment data argue for either bed-side acclimation protocols or scheduling of infusion within the validated post-thaw stability window of the chosen product, since pediatric patients with neuroinflammatory pathology represent precisely the population in which suboptimal MSC immunomodulatory potency would translate most directly into reduced clinical benefit [84]. Second, trial designs incorporating multiple infusions or sequential lots-an increasingly common feature of pediatric ASD studies-require demonstration of inter-lot consistency in the immunomodulation-relevant potency assays, since donor- and lot-to-lot variability in MSC immunosuppressive function remains the principal source of unexplained outcome heterogeneity in MSC trials across indications [109,110].

## 5. Preclinical Evidence for UC-MSC in ASD

### 5.1. ASD Animal Models in MSC Research

Translating UC-MSC therapy from bench to ASD-relevant clinical concepts requires preclinical evaluation in animal models that recapitulate the core behavioral and neurobiological features of the human disorder. The limits of that recapitulation should be borne in mind throughout this section. Rodent assays of sociability, vocalization, and repetitive behavior are operational proxies rather than homologues of the human diagnostic domains, and none of the models reproduces the developmental trajectory, genetic heterogeneity, or comorbidity profile of idiopathic ASD. Two features of these studies further limit extrapolation: the interventions are delivered in a short window after a defined insult in inbred animals of uniform age, whereas clinical administration occurs years after an unknown and heterogeneous set of causes; and several are delivered by intracerebroventricular (ICV) injection at doses per unit body weight far above those achievable clinically. Preclinical efficacy in these systems therefore establishes biological plausibility and identifies candidate mechanisms, and it does not predict clinical benefit.

Four model systems have dominated the MSC-in-ASD preclinical literature, each capturing different aspects of ASD pathophysiology: the BTBR T + Itpr3tf/J inbred mouse strain, which spontaneously displays social communication deficits and repetitive behaviors consistent with idiopathic ASD; the VPA-induced model, in which prenatal exposure to the antiepileptic teratogen reproduces social and repetitive deficits along with neuroinflammatory and synaptic changes; the MIA model, which uses prenatal poly I:C or lipopolysaccharide (LPS) challenge to model the epidemiologically established link between maternal infection and ASD risk; and the Shank3B knockout model, which represents the monogenic synaptopathy underlying Phelan–McDermid syndrome, in which approximately 75% of patients meet ASD criteria [111]. The four ASD animal models used in MSC research, their cell sources, and reported effects are summarized in Table 2. UC-MSC-specific preclinical evidence is unevenly distributed across these systems, concentrated most heavily in the VPA paradigm, with BTBR and Shank3B studies predominantly using BM-MSCs or their derivatives.

The VPA model has yielded the most robust direct evidence for UC-MSC therapeutic action in ASD-relevant behavior. Intravenous (IV) infusion of UC-MSCs into VPA-exposed rats produced significant improvement in sociability accompanied by attenuation of microglial activation in the anterior cingulate cortex, and UC-MSCs also promoted growth of primary cortical neurons in vitro, indicating coordinated immunomodulatory and trophic effects [113]. In VPA-exposed Kunming mice, ICV delivery of cord blood-derived MSCs (designated hUC-MSCs by the authors) alleviated social, repetitive, and anxiety-like behavioral abnormalities without organ-level toxicity, with hematoxylin and eosin (H&E) staining of major organs showing no abnormalities, and with mechanistic analyses linking the behavioral rescue to restored Insulin-like growth factor-1 receptor (IGF-1R)/protein kinase B (Akt) phosphorylation (Western blot), upregulated mRNA expression of synaptic plasticity genes (growth-associated protein 43 [GAP-43] and synaptophysin [SYP]) and anti-inflammatory IL-10, and suppression of pro-apoptotic Bcl-2-associated X protein (Bax)/cysteine-aspartic protease (caspase)-3 and pro-inflammatory IL-6/IL-1β mRNA [114]. A dual-target strategy combining systemic UC-MSCs with extracellular vesicles (EVs) in a VPA mouse model produced more comprehensive remediation than either monotherapy, synergistically normalizing neuroinflammatory cytokines and oxidative stress, restoring synaptic and mitochondrial ultrastructure, and shifting gut microbiota composition toward beneficial bacterial taxa—evidence that the UC-MSC effect engages a multi-tissue neuro–immune–microbiota axis rather than a single-target mechanism [115]. It should be noted that the two studies providing the most detailed mechanistic evidence in this model delivered cells intracerebroventricularly, a route confined to preclinical use in ASD (Section 7.4), so that the doses and central exposures achieved are not reproducible in children. Cell-free derivatives of UC-MSCs (exosomes) have been shown to recapitulate key behavioral benefits in the VPA model when delivered via the intranasal route, an observation examined in greater depth in Section 5.4 [116].

For the remaining animal models, UC-MSC-specific primary evidence is sparse, and the available literature relies primarily on MSCs from non-UC-tissue sources or their derivatives. In the BTBR strain, ICV transplantation of BM-MSCs reduced stereotypical behavior, decreased cognitive rigidity, and improved social behavior, with elevated hippocampal BDNF protein and increased hippocampal neurogenesis providing a candidate molecular substrate for the behavioral changes—establishing a foundational precedent that MSC-based intervention can engage the neurotrophic and neurogenic mechanisms relevant to ASD-like behavioral abnormalities [112]. In the Shank3B knockout model, intranasal administration of BM-MSC-derived exosomes improved social behavior deficits, vocalization, and repetitive behaviors in parallel with elevated gamma-aminobutyric acid (GABA) receptor subunit mRNA expression in the prefrontal cortex, indicating that MSC-based therapy can act in a monogenic synaptopathy, although no corresponding clinical observation in Phelan–McDermid syndrome has been reported [111]. Comparable UC-MSC-derived studies in the Shank3B or MIA paradigms are not yet available, representing the most prominent UC-MSC-specific evidence gap in the current preclinical landscape and a clear priority for future studies. The behavioral and neurodevelopmental outcomes documented across these model systems are examined in greater detail in the next sub-section.

### 5.2. Behavioral and Neurodevelopmental Outcomes

The behavioral phenotypes assessed across MSC-in-ASD preclinical studies map onto the diagnostic domains of human ASD: deficits in social interaction and communication, restricted and repetitive behaviors, cognitive inflexibility, and frequently co-occurring anxiety. Standardized rodent assays—including the three-chamber social approach test, dyadic reciprocal interaction, ultrasonic vocalization (USV) recording, self-grooming and marble-burying paradigms for repetitive behavior, T-maze and reversal-learning protocols for cognitive flexibility, and open-field or elevated plus-maze assays for anxiety-like behavior—form the common backbone of the preclinical evidence reviewed in Section 5.1. The therapeutic effects of MSC and MSC-derived products span all four behavioral domains, although the strength and consistency of evidence varies by model system and cell source.

Social behavior improvement is the most reproducibly reported outcome across UC-MSC studies. IV UC-MSCs in VPA-exposed rats significantly increased sociability while normalizing microglial activation patterns in the anterior cingulate cortex [113]. In VPA-exposed mice, ICV cord blood-derived MSCs produced significant gains in social interaction together with concurrent normalization of repetitive and anxiety-like behaviors [114], and a combined UC-MSCs + EV strategy in the same model yielded the most robust improvement in social interaction relative to either monotherapy [115]. Cell-free delivery of UC-MSC exosomes via the intranasal route similarly restored social ability in VPA-treated mice, indicating that the social-behavioral benefit is at least partly mediated by paracrine and exosomal cargo rather than cell engraftment per se [116]. In the BTBR strain, ICV MSC transplantation improved social behavior alongside reductions in cognitive rigidity [112], and intranasal BM-MSC-derived exosomes increased both male-to-male social interaction and male-to-female USVs in this strain, with additional restoration of pup-retrieval maternal behavior representing an unusual ASD-relevant outcome capturing the social-caregiving dimension of the disorder [117]. In the Shank3B knockout model of Phelan–McDermid syndrome, intranasal BM-MSC-derived exosomes improved social behavior deficits in multiple paradigms and increased vocalization frequency [111]. Comparable social-behavioral gains have been documented with AD-MSCs in the VPA mouse model, broadening the demonstration to non-UC MSC sources and reinforcing the conclusion that the behavioral improvement reflects shared MSC mechanisms rather than source-specific properties [45].

Restricted and repetitive behavior, the second core ASD domain, has shown a similarly consistent therapeutic response. The combined UC-MSCs + EV strategy in VPA-exposed mice reduced repetitive behaviors more comprehensively than either monotherapy [115], and cord blood-derived MSCs produced parallel reductions in repetitive and anxiety-like behaviors in the same model [114]. Cell-free UC-MSC exosomes intranasally administered to VPA-exposed mice corrected repetitive stereotyped behaviors alongside the social-behavioral gains [116]. In the BTBR strain, MSC transplantation reduced stereotypical behavior and decreased cognitive rigidity-one of the few direct demonstrations of cognitive flexibility rescue in the MSC-in-ASD literature [112]. Intranasal BM-MSC-derived exosomes reduced repetitive behaviors in both the BTBR strain [117] and the Shank3B knockout model [111]. AD-MSC studies have confirmed the reduction in repetitive behavior and anxiety-like phenotypes in the VPA model, with mechanistic analyses linking the behavioral rescue to restored phosphatase and tensin homolog (PTEN)/p-Akt signaling and elevated VEGF and IL-10 expression in the brain [45].

Neurodevelopmental and molecular outcomes accompany and provide candidate substrates for these behavioral gains. The earliest demonstration in the BTBR strain linked BM-MSC-induced behavioral improvement to elevated hippocampal BDNF protein and increased hippocampal neurogenesis, establishing a neurotrophic–neurogenic axis as one mechanism by which BM-MSC engagement of resident neural progenitor populations might support functional recovery [112]. Subsequent mechanistic work in the VPA paradigm has expanded this picture: cord blood-derived MSCs in VPA-exposed mice promoted cortical neuronal dendritic development and upregulated synaptic plasticity markers GAP-43 and SYP, linking the behavioral gains to a coordinated synaptic-trophic response in the postnatal cortex [114]. The combined UC-MSCs + EV strategy restored synaptic and mitochondrial ultrastructure in key brain regions of VPA-exposed mice as documented by transmission electron microscopy, demonstrating that the molecular response includes restoration of nano-scale subcellular architecture rather than only protein-level expression changes [115]. In the Shank3B knockout model, intranasal BM-MSC-derived exosomes increased GABA receptor subunit mRNA expression in the prefrontal cortex, providing a candidate molecular substrate involving inhibitory neurotransmission rescue [111]. Exosome-mediated neurogenesis enhancement has been mechanistically dissected using BTBR mice receiving human AD-MSC-derived exosomes, where long non-coding RNA interferon gamma antisense RNA 1 (IFNG-AS1) was shown to act as a molecular sponge for microRNA-21a-3p (miR-21a-3p) and promote neurogenesis through the miR-21a-3p/PI3K/Akt signaling axis [118]. The convergent role of the PI3K/Akt pathway across studies—observed with cord blood-derived MSCs (IGF-1R/Akt) [114], AD-MSCs (PTEN/p-Akt) [45], and AD-MSC exosomes (miR-21a-3p/PI3K/Akt) [118]—suggests a shared signaling node for MSC-derived therapeutic intervention in ASD, examined in greater mechanistic depth in the next sub-section.

### 5.3. Mechanistic Insights from UC-MSC Studies

The behavioral and neurodevelopmental rescue documented across UC-MSC preclinical studies (Section 5.1 and Section 5.2) is supported by mechanistic data spanning four interconnected axes: immunomodulation with microglial repolarization, cytokine rebalancing, neurotrophic and neurogenic signaling, and gut–brain axis restoration—a distinctive feature of MSC-based intervention in this context. These axes connect directly to the UC-MSC biological properties detailed in Section 3.4 and Section 3.5, providing in vivo confirmation in ASD models of mechanisms first characterized in vitro and in non-neural disease contexts.

Microglial repolarization from a pro-inflammatory M1 state to an anti-inflammatory M2 state is the most reproducibly documented immunomodulatory effect of UC-MSC in ASD-relevant brain pathology. IV UC-MSCs in VPA-exposed rats significantly attenuated microglial activation specifically in the anterior cingulate cortex [113]. The combined UC-MSCs + EV approach in VPA mice synergistically normalized neuroinflammatory cytokine levels and oxidative stress markers, indicating that the microglia-targeted effect of UC-MSC extends to the broader neuroinflammatory milieu [115]. Mechanistic dissection in non-ASD neuroinflammation contexts has identified molecular pathways relevant to microglial repolarization in the presence of UC-MSCs: in LPS-treated mice, UC-MSCs combined with mitsugumin 53 shifted microglial polarization from M1 (inducible nitric oxide synthase-high) toward M2 (arginase 1-high) with suppressed expression of TNF-α, TLR4, nucleotide oligomerization domain-like receptor protein 3 (NLRP3), cleaved caspase-1, and IL-1β, effects that the authors attributed to mitsugumin 53 acting through the NLRP3/caspase-1/IL-1β inflammasome axis to protect the cells and facilitate their efficacy [119]. In a chronic unpredictable mild stress (CUMS) model of major depression, UC-MSCs reprogrammed microglial polarization by inhibiting complement C3a/C3aR signaling during astrocyte–microglia crosstalk, demonstrating that UC-MSCs engage glial cell–cell communication networks beyond direct cell–cytokine interactions [120]. Although neither of the latter two studies used an ASD model, they identify shared molecular substrates—NLRP3 inflammasome activation and complement-driven microglial polarization—that overlap with the neuroinflammatory features of ASD outlined in Section 2.

Cytokine rebalancing accompanies and partly explains the microglial effects. ICV cord blood-derived MSCs in VPA-exposed mice produced a coordinated cytokine shift: anti-inflammatory IL-10 upregulated and pro-inflammatory IL-6 and IL-1β suppressed, alongside reduction of pro-apoptotic Bax and Caspase-3 [114]. The combined UC-MSCs + EV strategy in the same model normalized neuroinflammatory cytokines while restoring synaptic and mitochondrial ultrastructure, linking the cytokine shift to subcellular architectural recovery [115]. AD-MSC studies in the VPA mouse model produced a comparable cytokine pattern with elevated brain IL-10 and VEGF after human AD-MSC transplantation, reinforcing the conclusion that the cytokine-balancing effect is shared across MSC sources [45].

Neurotrophic signaling and neurogenesis form the second major mechanistic axis. The earliest BTBR demonstration linked MSC-induced behavioral improvement to elevated hippocampal BDNF protein and increased hippocampal neurogenesis, establishing a neurotrophic–neurogenic axis as one mechanism by which MSC engagement supports functional recovery [112]. Subsequent work in the VPA paradigm has elaborated this picture: cord blood-derived MSCs in VPA-exposed mice promoted cortical neuronal dendritic development with restoration of IGF-1R/Akt phosphorylation and upregulation of synaptic plasticity-associated genes (GAP-43 and SYP), tying behavioral rescue to a coordinated synaptic-trophic response in the postnatal cortex [114]. Direct in vitro co-culture has confirmed the trophic capacity, with UC-MSCs promoting growth of primary cortical neurons in a Transwell migration assay design—evidence that the neurotrophic effect operates through soluble paracrine factors rather than requiring direct cell contact [113]. Cell-free derivatives can recapitulate the neurogenic effect: exosomal long non-coding RNA IFNG-AS1 derived from AD-MSCs (containing lncRNAs that overlap with the UC-MSC exosomal cargo) acts as a molecular sponge for miR-21a-3p and facilitates neurogenesis through the miR-21a-3p/PI3K/Akt signaling pathway [118]. Synaptic-level effects have also been documented: in the Shank3B knockout model, intranasal BM-MSC-derived exosomes elevated GABA receptor subunit mRNA expression in the prefrontal cortex, providing a candidate molecular substrate involving inhibitory neurotransmission rescue relevant to the excitation–inhibition imbalance commonly observed in ASD synaptic models [111].

A distinctive feature of UC-MSC mechanistic action in ASD is engagement of the gut–brain axis. The combined UC-MSCs + EV approach in VPA-exposed mice promoted gut microbiota homeostasis by enriching beneficial bacterial taxa such as Lactobacillus and reducing pathogens, with the gut-microbiota shift accompanied by normalized neuroinflammatory and synaptic markers in the brain [115]. This observation aligns with the broader recognition that gut microbiota dysbiosis is implicated in ASD pathophysiology, and identifies UC-MSC as a candidate therapeutic capable of acting along the neuro-immune–microbiota axis rather than only on CNS targets. Across the four mechanistic axes, a recurring convergent signaling node is the PI3K/Akt pathway: cord blood-derived MSCs act via the IGF-1R/Akt branch [114], AD-MSCs via the PTEN/p-Akt branch [45], and AD-MSC exosomes via the miR-21a-3p/PI3K/Akt branch [118]. The convergence of three mechanistically distinct cell preparations on the PI3K/Akt pathway suggests this signaling node as a shared downstream effector of MSC-derived therapeutic intervention in ASD, with potential implications for biomarker development and dose optimization. The cell-free derivatives that recapitulate many of these mechanistic effects—exosomes and EVs—are examined as a distinct therapeutic modality in the next sub-section.

### 5.4. Cell-Free Derivatives: UC-MSC Exosomes and EVs

The therapeutic effects of MSCs have been increasingly attributed to paracrine action mediated by EVs and exosomes—nano-sized membrane-bound particles enriched with bioactive cargo including microRNAs, mRNAs, neurotrophic and immunomodulatory proteins, and lipid mediators—rather than to long-term cell engraftment, motivating the systematic evaluation of cell-free MSC derivatives as therapeutic agents for ASD [23,121,122]. Cell-free formulations offer practical advantages over whole-cell products: a low immune response relative to other biological carriers, storage and shipping requirements less restrictive than those of viral vectors, and—most importantly for pediatric neurological indications—ready compatibility with non-invasive intranasal delivery, after which MSC-derived exosomes enter the brain and accumulate within neural cells [122,123]. Direct preclinical evidence supports the therapeutic potential of MSC-derived exosomes across ASD model systems. In the VPA mouse model, intranasal administration of UC-MSC-derived exosomes restored social ability and corrected repetitive stereotyped behaviors with anti-inflammatory effects in offspring of VPA-treated dams [116], and the combined UC-MSCs plus EV strategy produced the most robust improvements relative to either monotherapy, indicating synergy between cellular and cell-free components [115]. In a VPA rat model, IV MSC-derived exosomes mitigated cerebellar histopathological alterations and behavioral changes through anti-apoptotic and anti-inflammatory effects coupled with modulation of PI3K- mechanistic target of rapamycin (mTOR) signaling [124]. In the BTBR strain, intranasal BM-MSC-derived exosomes increased male-to-male social interaction and male-to-female USV while restoring maternal pup-retrieval behavior [117], and human AD-MSC-derived exosomes carrying lncRNA IFNG-AS1 promoted neurogenesis and improved ASD-like behavior through the miR-21a-3p/PI3K/Akt axis—with bioinformatic analysis demonstrating substantial overlap between the human AD-MSC- and UC-MSC-exosome lncRNA cargoes [118]. In the Shank3B knockout model of Phelan–McDermid syndrome, intranasal BM-MSC-derived exosomes improved social, communicative, and repetitive behaviors with elevated prefrontal GABA receptor subunit mRNA expression [111]. A 2026 study extended this evidence using human induced pluripotent stem cell (iPSC)-derived cortical neurons with hemizygous SHANK3 deletion and Shank3B^−/−^ mice: EVs from MSCs and from healthy iPSCs rescued the hyperexcitability and accelerated maturation phenotypes of SHANK3-deficient neurons through delivery of synaptic modulators (C1R, C1S, MDK, IGFBP3) and homeostatic regulators (FGF2, SFRP1), and intranasal iPSC-derived EVs significantly rescued ASD-like behavioral deficits in Shank3B^−/−^ mice-the most mechanistically detailed demonstration of EV-mediated cellular phenotype rescue in monogenic ASD to date [125]. Biodistribution and brain-targeting properties of MSC-derived exosomes have been characterized using gold nanoparticle labeling and X-ray computed tomography imaging. Intranasally administered BM-MSC-derived exosomes specifically accumulated in pathologically relevant brain regions in murine models of stroke, autism, Parkinson’s disease, and Alzheimer’s disease for up to 96 h, whereas in healthy controls the same exosomes showed diffuse migration with clearance by 24 h; neuroinflammatory signal correlated with MSC-exosome accumulation, suggesting that the homing mechanism is inflammation driven and that exosome uptake is selectively neuronal rather than glial [126]. The non-invasive intranasal delivery route avoids surgical intervention while overcoming the BBB-imposed limitations of systemic delivery—a critical advantage relative to alternative gene-therapy delivery platforms such as adeno-associated viruses (AAVs), which face additional immunogenicity and packaging-capacity constraints in neurodevelopmental indications [122]. Taken together, the cell-free derivative literature establishes MSC-exosome and MSC-EVs as a distinct therapeutic modality that recapitulates the behavioral, neurodevelopmental, and mechanistic effects of whole-cell MSC therapy in ASD models, with practical advantages well suited to pediatric clinical translation [23]. The convergent evidence across BTBR, VPA, and Shank3B models—and across MSC sources including UC, BM, AD, and iPSC derivatives—supports a shared therapeutic axis operating through immunomodulatory, neurotrophic, and synaptic-regulator cargo. This convergence is a statement about mechanism in animals and not about clinical effect: the behavioral gains reported here are large and consistent, whereas the only adequately powered placebo-controlled trial in pediatric ASD did not meet its primary endpoint (Section 6.4), and the discrepancy is itself the reason preclinical consistency cannot be read as evidence of clinical efficacy. Translating this preclinical foundation into clinical application requires the safety and efficacy validation provided by human trials, examined in Section 6.

## 6. Clinical Evidence for UC-MSC in Pediatric ASD

### 6.1. Allogeneic UC-MSC Clinical Trials

A first-in-children clinical evaluation of umbilical cord-derived stem cells in ASD was undertaken in 2013 as a non-randomized, open-label, single-center Phase I/II trial in China. Thirty-seven children with ASD were assigned to one of three arms: cord blood mononuclear cell (CBMNC) infusion plus rehabilitation, combined CBMNC plus UC-MSC infusion plus rehabilitation, or rehabilitation alone, with four stem cell infusions delivered intravenously and intrathecally at weekly intervals. Both treatment arms—CBMNC alone and combined CBMNC + UC-MSC—produced significantly greater improvements on the Childhood Autism Rating Scale (CARS), Aberrant Behavior Checklist (ABC), and Clinical Global Impression (CGI) at 24 weeks compared with the rehabilitation-only control, with the combination arm showing larger therapeutic effects than CBMNC alone, and no severe adverse events occurred across all 37 participants during the monitoring period—establishing both the feasibility and short-term safety of UC-MSC-containing therapy in this population [20]. This trial (NCT01343511) provided the foundational human evidence supporting subsequent allogeneic UC-MSC investigations in pediatric ASD.

The Duke University group conducted a Phase I open-label single-arm study of allogeneic third-party-donor UC-MSCs in 12 children with ASD aged 4 to 9 years to assess safety and feasibility in this population. Participants received one, two, or three IV infusions of 2 × 10^6^ cells per kilogram at two-month intervals, with follow-up at 6 months in person and 12 months remotely after the final dose. UC-MSCs were well tolerated overall, with agitation during IV placement in some participants. Two participants experienced product-related events: one developed a hypersensitivity reaction with mild hypotension during a second infusion given after a requested omission of diphenhydramine premedication, which resolved completely and after which the infusion was completed following diphenhydramine administration, and one developed moderate hypotension after a third infusion, which resolved with intravenous fluids. A further 66 non-serious adverse events not specifically attributed to the study product were reported in eleven of the twelve participants. Five of twelve participants developed new class I anti-HLA antibodies that were associated with a specific cell lot or with a partial donor–recipient HLA match, but these antibodies were clinically silent and not associated with any adverse clinical manifestations. Six of twelve participants demonstrated improvement in at least two ASD-specific measures, and the authors concluded that UC-MSC manufacturing and administration appeared safe and feasible in young children with ASD—paving the way for a subsequent Phase II randomized placebo-controlled trial [22]. That Phase II trial has since been completed (NCT04089579): 137 children aged 4 to 11 years were randomized to receive a single intravenous dose of 6 × 10^6^ hCT-MSC per kilogram either at baseline or, after placebo, at six months, under quadruple masking. Results posted to the trial registry show that the primary endpoint, the mean change in the VABS-3 Socialization and Communication subscale standard scores from baseline to six months, was not met (3.35 versus 2.03 points; net mean difference 1.32; *p* = 0.35), nor were the Socialization (*p* = 0.12) or Communication (*p* = 0.83) subscales individually, the Clinical Global Impression scales, or quality of life. A post hoc responder analysis found a higher proportion of children achieving a Socialization subscale gain of at least three points in the treated arm (55% versus 35%; relative risk 1.57; *p* = 0.02), while the corresponding Communication responder analysis did not differ (44% versus 40%; *p* = 0.64). A peer-reviewed report of this trial had not appeared at the time of writing, and the findings are therefore cited from the registry record.

The anti-HLA antibody finding documents donor–recipient HLA mismatch immunogenicity in pediatric UC-MSC therapy without associated clinical sequelae in that trial, and whether donor–recipient HLA matching or lot consistency affects outcomes under repeat dosing has not been examined (see also Section 4.4 on banking and lot consistency).

Subsequent case-level evidence has expanded the geographic and product diversity of UC-MSC therapeutic experience in pediatric ASD. A Turkish case report described a child who underwent intrathecal (IT) and IV WJ-MSC transplantation combined with neurorehabilitation, showing significant improvement on the CARS and the Denver II Developmental Screening Test—particularly in language and gross motor skills—with no serious adverse events during a 2-year follow-up [127]. An Israeli–Serbian collaboration reported a boy with severe ASD, epilepsy, and a pathological electroencephalography (EEG) who received five courses of allogeneic MSCs from unrelated-donor placenta and umbilical cord tissue between ages 5.75 and 9 years, with each course producing a significant clinical response including disappearance of seizures, normalization of the EEG after the first course, and continuously improved ASD symptoms, social skills, and emotional expression [128]. Both reports are uncontrolled single-patient observations in which the reported changes cannot be separated from concurrent neurorehabilitation, concomitant medication, or spontaneous developmental and clinical change, and neither supports an inference of efficacy. For methodological comparison, a randomized controlled trial (RCT) in Iran used autologous BM-MSCs delivered intrathecally in 32 children with ASD, finding no significant between-group differences in CARS, the Gilliam Autism Rating Scale, Second Edition (GARS-II), or CGI improvement over 12 months but a significant time-by-group interaction favoring the intervention group on the CGI severity dimension—demonstrating that an IT autologous MSC approach using a non-UC source achieved safety but limited efficacy in this population [46].

The accumulated UC-MSC and broader MSC clinical evidence has been synthesized in meta-analysis. Pooling five eligible studies (including BM, UCB, and umbilical cord-derived products) against rehabilitation-therapy reference standards, the meta-analysis reported no significant difference in adverse-event rates between intervention and control arms (RR 1.55; 95% CI 0.60–3.98)—concluding that stem cell therapy for children with ASD may be safe and effective but that the evidence is compromised by small study sizes, heterogeneous injection routes and doses, and short follow-up windows [129]. These limitations are amplified by the cautionary precedent set by a 2019 single-arm Phase I/II study of repeated allogeneic UC-MSC infusions in pediatric ASD that was subsequently retracted from the literature-a development that reinforces the need for independent replication, rigorous reporting standards, and conservative interpretation of UC-MSC efficacy signals in pediatric ASD. A separate but important distinction concerns whether the source material is UCB or UC-MSCs, a clinically meaningful question with different cellular compositions and likely different mechanisms of action that is examined in detail in the next sub-section. The clinical studies of umbilical cord-derived and comparator MSC therapies in pediatric ASD are summarized in Table 3.

### 6.2. The UCB vs. UC-MSC Distinction: A Critical Clarification

#### 6.2.1. Biological and Cellular Differences

The umbilical cord-derived therapeutic landscape encompasses two product classes that share only the anatomical source: UCB and UC-MSCs. UCB is a heterogeneous mononuclear cell suspension containing CD34^+^ hematopoietic stem cells, CD14^+^ monocytes, lymphocytes, granulocyte precursors, and a very small proportion of MSCs, with total cellular content conventionally enumerated as total nucleated cell count (TNCC). Cord blood banks routinely quantify TNCC and CD34^+^ cell content for clinical product release but do not measure CD14^+^ monocyte content as standard practice [38]. The CD34^+^ dose has historically served as the principal selection parameter for UCB transplant indications, with the original transplant standard established at a minimum of 1.7 × 10^5^ CD34^+^ cells per kilogram of recipient body weight in unrelated-donor UCB grafts disparate in no more than 2 HLAs-a threshold derived from outcome analyses on engraftment, treatment-related mortality (TRM), and survival in 102 pediatric and adult recipients with both malignant and nonmalignant indications, with UCB units below this threshold considered inadequate for routine use [132]. UCB is therefore biologically defined by its composite cellular content rather than by a single homogeneous cell type. The biological and cellular distinctions between UCB and UC-MSCs are summarized in Table 4.

UC-MSCs by contrast are a purified, characterized MSC population isolated from the connective tissue of the umbilical cord, predominantly WJ (cf. Section 3.1 on UC-MSC source biology). The ISCT position statement defines minimal criteria for human MSCs that have been adopted as the regulatory and academic standard worldwide: MSCs must be plastic-adherent under standard culture conditions, must express CD105, CD73, and CD90, must lack expression of the hematopoietic and monocytic markers CD45, CD34, CD14 (or CD11b), CD79α (or CD19), and HLA-DR, and must differentiate into osteoblasts, adipocytes, and chondroblasts in vitro [33]. The explicit exclusion of CD14^+^ cells from any product meeting the ISCT MSC definition has direct implications for the UCB vs. UC-MSC comparison: by criterion, an ISCT-compliant UC-MSC product cannot contain the CD14^+^ monocyte subset that mediates UCB therapeutic effects in neuroinflammatory indications (cf. Section 6.2.3). The release specifications and manufacturing controls established for UC-MSC products (cf. Section 4 on manufacturing and quality) operate at the level of the entire characterized cell population, in contrast to the variable CD14^+^ content of UCB units that is neither measured nor controlled.

These compositional differences carry biological consequences. Within UCB, the CD14^+^ monocyte subset has been identified as the cell type responsible for the documented therapeutic effects in CNS injury models, while CD14^+^ monocytes from adult peripheral blood do not share this activity—indicating that the therapeutic benefit of UCB depends on a minor and unmeasured subpopulation of developmentally distinct fetal-origin cells [133]. UC-MSC products, by virtue of ISCT-mandated exclusion of CD14^+^ cells, cannot act through the same cellular substrate, and are expected instead to operate through the immunomodulatory and neurotrophic mechanisms of a purified mesenchymal population detailed in Section 3.4 and Section 3.5. UCB and UC-MSC are therefore not interchangeable products with shared mechanisms but distinct cellular therapies with different candidate active components, different dose-defining parameters, and different manufacturing standards. It should be noted that the inference runs in one direction only: the identification of the CD14^+^ monocyte as the active fraction of UCB establishes that UCB activity depends on a minor and unmeasured subpopulation, which makes UCB difficult to standardise, but it is not itself evidence that the purified MSC population is the therapeutic agent in UC-MSC products. The comparative clinical evidence reviewed in the next sub-section is examined with that limitation in view.

#### 6.2.2. Clinical Evidence Comparison

The UCB and UC-MSC clinical trial programs have evolved largely in parallel, with distinct sponsors, dose paradigms, and outcome measures. UCB evidence is dominated by trials from the Duke University group, which conducted the most systematic clinical evaluation. A Phase I open-label trial in 25 children aged 2 to 6 years with a confirmed ASD diagnosis and a qualified banked autologous UCB unit demonstrated that single IV UCB infusion was safe and well tolerated, with significant improvements at 6 months on parent-reported social communication, clinician overall severity ratings, expressive vocabulary, and objective eye-tracking measures of social attention, with higher baseline nonverbal IQ predicting greater response [130]. Subsequent biomarker analyses on the same cohort identified that EEG spectral changes—increased alpha and beta power and decreased theta power—emerged by 12 months, with higher baseline posterior beta power predicting social communication improvement [134]. Diffusion tensor imaging in 19 of these participants further demonstrated that behavioral improvements were associated with increased white matter connectivity in frontal, temporal, and subcortical regions (hippocampus and basal ganglia) implicated in ASD [135].

These open-label findings motivated a definitive Phase II evaluation. A randomized, placebo-controlled, double-blind trial in 180 children aged 2 to 7 years compared autologous UCB (*n* = 56), allogeneic UCB (*n* = 63), and placebo (*n* = 61) with evaluation at 6 months postinfusion. UCB infusion was safe and well tolerated, but no improvement was demonstrated on the primary outcome (the Vineland Adaptive Behavior Scales, Third Edition [VABS-3] Socialization Domain) or on secondary outcomes (Pervasive Developmental Disorder [PDD] Behavior Inventory and expressive vocabulary) in the entire sample, and the differential efficacy by UCB type was not significant overall. A post hoc subanalysis in children without ID identified that allogeneic-but not autologous-UCB was associated with improvement in a larger percentage of children on the clinician-rated CGI-Improvement scale, and with significant gains on communication, attention to toys, sustained attention by eye-tracking, and EEG alpha and beta power, although the odds ratio for clinician-rated improvement was not statistically significant-a finding the authors interpreted as warranting further investigation rather than supporting general efficacy [21]. Convergent evidence from an independent placebo-controlled crossover trial in 29 children aged 2 to 6 years receiving autologous UCB versus placebo showed trends toward socialization improvement but no statistically significant differences for any endpoint, with consistent safety across infusions [136].

A separate clinical investigation has yielded contrasting positive signals in specific age and weight subgroups. The CORDUS study in Romania administered autologous cord blood (ACB) and individualized supplements sequentially in a crossover design to 56 children with ASD. ACB improved evaluation scores in 78% of children aged 3 to 7 years (*n* = 28) but in only 11% of those older than 8 years or weighing more than 35 kg (*n* = 28), and high baseline inflammatory markers and ferritin were associated with reduced response. Improvements reached statistical significance on verbalization and social interaction but not on irritability or aggressive behavior. The study thus identifies age, body weight and inflammatory phenotype as candidate effect modifiers worthy of further mechanistic study [131]. Associated biomarker analysis of the CORDUS cohort found neuron-specific enolase (NSE) elevated above the clinical reference range of 16.3 ng/mL in 40 of 41 ASD children (97.5%), positioning NSE as a candidate diagnostic and dynamic-evaluation biomarker for ASD with potential utility in cell therapy response prediction [137].

Comparing this UCB evidence base with the UC-MSC clinical evidence reviewed in Section 6.1 reveals a divergent pattern. The UC-MSC program is smaller in sample size and dominated by Phase I and case-level data-the Lv et al. 2013 [20] Phase I/II trial in 37 children showed significant improvements in both CBMNC and CBMNC + UC-MSC combination arms versus rehabilitation-only control at 24 weeks, and the Duke Phase I trial of 12 children receiving UC-MSC infusions reported improvement in six of twelve participants on at least two ASD-specific measures (cf. Section 6.1)—whereas the UCB program is dominated by larger Phase I and Phase II trials with mixed and partly negative primary outcomes. Direct comparison is limited by heterogeneous outcome measures (CARS/ABC/CGI in UC-MSC trials; VABS/PDD/Expressive One Word Picture Vocabulary Test, Fourth Edition [EOWPVT] in UCB trials), differing follow-up windows (6 months in UCB Phase II vs. 24 weeks–2 years in UC-MSC trials), and the absence of any head-to-head randomized comparison. The two product classes have not been evaluated against each other in any clinical setting, and the available evidence base permits only qualitative rather than quantitative comparison of efficacy signals. The mechanistic basis for the divergent clinical performance—the identity of the active cell type within each product class—is examined in the next sub-section.

#### 6.2.3. The Duke Letter and CD14^+^ vs. CD34^+^ Debate

A pivotal correspondence in The Journal of Pediatrics in 2021 shifted the mechanistic understanding of UCB efficacy in pediatric ASD and clarified the implications of the negative Phase II findings reviewed in Section 6.2.2. A letter to the editor by the Vinmec group (Vietnam) raised three methodological concerns regarding the Dawson et al. Phase II trial: first, that the administered CD34^+^ cell doses (0.3 × 10^5^ cells/kg for autologous UCB and 0.7 × 10^5^ cells/kg for allogeneic UCB) were substantially below the minimum effective threshold of 1.7 × 10^5^ CD34^+^ cells/kg suggested by prior investigators in nonmalignant disease indications, citing the Wagner et al. [132] cohort (cf. Section 6.2.1); second, that IV systemic delivery may have limited brain delivery through cell trapping in lung, heart, liver, and kidney; and third, that the 6-month follow-up was too short relative to the 12–18 month windows in which prior UCB ASD studies had documented progressive improvement on CARS and CGI [138]. The letter recommended future research using UCB with higher CD34^+^ cell doses, multiple infusions, and alternative administration routes.

The Duke group reply was mechanistically decisive and recast the entire CD34^+^ framing as a measurement artifact rather than the active dose parameter. The authors stated that their preclinical studies and early-phase clinical trial data in children with ASD and cerebral palsy (CP) had shown no association between improvement and CD34^+^ dose, and instead identified the CD14^+^ monocyte in cord blood as the cell type responsible for modulation of neuroinflammation, stimulation of oligodendrocyte proliferation, remyelination, and increased whole brain connectivity—a position grounded in the operational observation that cord blood banks measure TNCC and CD34 but not CD14, leading the Duke group to base UCB unit selection in ASD and CP trials on TNCC dose rather than on CD34^+^ dose [38].

The reply further reported that the Duke Phase II randomized trial of autologous UCB in young children with CP had identified an effective dose threshold at 25 million TNCC/kg, with a dose–response trend extending to 100 million cells/kg in subsequent unpublished work, and that ASD trials targeted a minimum of 25 million TNCC/kg-a dose paradigm fundamentally different from the CD34^+^-based selection criticized in the Vinmec letter. The Sun et al. 2017 CP trial, a double-blind, placebo-controlled crossover study of single IV ACB in 63 children with CP, provided the supporting evidence: although the primary endpoint of mean Gross Motor Function Measure-66 (GMFM-66) change at 1 year was not significant overall, participants who received doses of at least 2 × 10^7^ TNCC/kg demonstrated significant gains over predicted values on GMFM-66, Peabody Developmental Motor Scales-2 Gross Motor Quotient (PDMS-2), and normalized brain connectivity—establishing the TNCC dose threshold paradigm that Duke subsequently extended to ASD trials [139].

Primary preclinical research from the Duke laboratory provides the mechanistic foundation for the CD14^+^ monocyte hypothesis. A cord blood monocyte-derived cell therapy product (DUOC-01), developed from banked human UCB CD14^+^ monocytes via 2–3 weeks of in vitro culture, accelerated remyelination in non-obese diabetic (NOD)/severe combined immunodeficiency (SCID)/IL-2Rγ mice following cuprizone feeding-mediated demyelination. Compared with excipient controls, treated animals showed enhanced myelination in the corpus callosum, a higher proportion of fully myelinated axons, decreased gliosis and cellular infiltration, and more proliferating oligodendrocyte lineage cells. Uncultured UCB CD14^+^ monocytes also accelerated remyelination but to a significantly lesser extent, indicating that culture-driven upregulation of pro-remyelination factors (PDGF-AA, stem cell factor, IGF-1, MMP-9, MMP-12, and triggering receptor expressed on myeloid cells-2 [TREM-2]) underlies the enhanced activity of DUOC-01 [140]. Subsequent manufacturing characterization confirmed that the macrophages comprising DUOC-01 arise specifically from CD14^+^ monocytes within the CBMNC fraction, with whole transcriptome microarray analysis, quantitative PCR, and Bioplex protein quantification all demonstrating rapid upregulation of multiple gene products promoting remyelination within 14 days of culture from either MNC or immunoselected CD14^+^ monocyte starting populations [141]. A complementary mechanistic study using mouse brain organotypic slice cultures subjected to oxygen-glucose deprivation demonstrated that CBMNCs—but not adult peripheral blood MNCs—protected neurons from injury-induced death and reduced microglia and astrocyte activation, with subpopulation depletion identifying UCB CD14^+^ monocytes as the cell type whose removal abolished neuroprotection; purified UCB CD14^+^ monocytes (but not purified adult PB CD14^+^ monocytes) recapitulated the protective activity, with differential expression of chitinase 3-like protein-1, inhibin-A, IL-10, MMP-9, and thrombospondin-1 identifying candidate effector molecules and establishing UCB CD14^+^ monocytes as the critical cell type for UCB therapeutic action in CNS injury [133].

These findings carry direct implications for the UCB vs. UC-MSC distinction central to this review. UCB therapeutic activity in pediatric neurological indications is mediated by a developmentally distinct fetal-origin CD14^+^ monocyte subpopulation that constitutes a minor and unmeasured fraction of every UCB unit, with the dominant CD34^+^ HSC component-the parameter conventionally quantified by cord blood banks-showing no relationship to response in ASD or CP. UC-MSC products, by virtue of ISCT minimal criteria requiring exclusion of CD14^+^ cells (cf. Section 6.2.1), cannot deliver therapeutic activity through the same mechanism and operate instead through the immunomodulatory and neurotrophic actions of a homogeneous mesenchymal cell population (cf. Section 3.4 and Section 3.5). The choice between UCB and UC-MSC is therefore not a choice between products with shared mechanisms differing only in convenience or cost, but a choice between two distinct cellular therapies with non-overlapping candidate active components, dose-defining parameters, and manufacturing standards (Figure 2). It should be emphasized that this account is mechanistic rather than clinical. No trial has yet been designed to test whether the purified MSC population, rather than another fraction of the cord-derived product, is responsible for the clinical changes observed in pediatric ASD, and the proposition therefore remains a hypothesis that the available evidence renders plausible but does not confirm. Safety and adverse-event considerations across both product classes are examined in the next sub-section.

### 6.3. Safety Profile Across Clinical Trials

The aggregate clinical experience with UC-MSC and UCB cell therapies in pediatric ASD now encompasses several hundred dosed participants across Phase I, Phase II, and case-level studies, establishing a consistent acceptable safety profile across heterogeneous protocols, doses, sources, and administration routes. Direct UC-MSC exposure data come from the Lv et al. Phase I/II trial in 37 children with ASD (combined CBMNC and UC-MSC infusions delivered intravenously and intrathecally over four weekly sessions, with no significant safety issues and no severe adverse effects across the monitoring period [20]) and from the Duke Phase I trial of UC-MSC infusions in 12 children with ASD (one, two, or three IV infusions of 2 × 10^6^ cells/kg at 2-month intervals, with the infusion-related events and the clinically silent anti-HLA antibody responses detailed in Section 6.1 [22]). Case-level evidence reinforces this profile: a Turkish case report described 2-year safety follow-up after IT and IV WJ-MSC transplantation with no serious adverse events [127], and an Israeli–Serbian case report documented five sequential courses of allogeneic placenta and UC-MSC administered to a child with severe ASD plus epilepsy, with significant clinical response and no safety concerns reported across treatment courses spanning ages 5.75 to 9 years [128].

The UCB clinical experience in pediatric ASD covers a substantially larger participant population. The Duke open-label Phase I trial in 25 children aged 2–6 years demonstrated that single IV autologous UCB infusion was safe and well tolerated across 12 months of follow-up [130], with subsequent biomarker analyses on the same cohort [134,135]. The Phase II randomized, placebo-controlled, double-blind trial in 180 children (56 autologous UCB, 63 allogeneic UCB, 61 placebo) reported that UCB infusion was safe and well tolerated at the 6-month evaluation point, with no excess of serious adverse events relative to placebo [21]. The independent placebo-controlled crossover trial in 29 children aged 2–6 years similarly reported no serious adverse events across infusions [136], and the CORDUS study in 56 children reported no serious adverse reactions during or after ACB administration [131]. Pediatric UCB experience also extends to CP, where the Duke Phase II crossover trial of single IV autologous UCB in 63 children with CP provided additional pediatric experience [139]. The cumulative pediatric UCB exposure across these trials exceeds 350 dosed participants without demonstration of dose-limiting or treatment-related serious adverse events attributable to UCB infusion itself.

Adverse events when observed have been predominantly mild and self-limiting. Acute infusion-related reactions documented in MSC trials more broadly include transient fever, headache, and fatigue—the SafeCell systematic review of 36 clinical trials encompassing 1012 participants across multiple indications, with meta-analysis of the 8 RCTs (321 participants), identified no association between intravascular MSC administration and acute infusional toxicity, organ system complications, infection, death, or malignancy, with the only significant signal being a non-serious association with transient fever [142]. Comparator data from non-UC stem cell sources contextualize the favorable UC-MSC and UCB profiles: a RCT of IT autologous BM-MSCs in 32 children with ASD documented safety and feasibility with limited efficacy [46], while an Indian open-label trial of IT autologous bone marrow mononuclear cells (BMMNCs) in 32 children with ASD reported few adverse events including controlled seizures in three patients managed with medications [48]—a finding that contrasts with the absence of seizure signals in UC-MSC and UCB trials reviewed above and highlights the relevance of administration route and cell preparation type to the safety profile. The favorable IT MSC route safety profile is further supported by a dedicated systematic review of 39 studies of spinal IT MSC injection across neurological conditions—including autism, multiple sclerosis, and traumatic brain injury—which identified no serious adverse events such as infections, clinical rejection, or tumors, with the authors concluding that properly performed spinal IT injection of MSCs is exceedingly safe [143].

The meta-analytic evidence supports the descriptive trial-level data. The first dedicated meta-analysis of stem cell therapy for childhood ASD pooled five eligible studies and found no significant difference in the incidence of adverse reactions between intervention and rehabilitation-control arms (RR 1.55, 95% CI 0.60–3.98, *p* = 0.36) [129]. A more recent March 2026 synthesis comparing safety profiles and preliminary efficacy outcomes across nine registered early-phase trials (Phase I and II) of MSC-based therapies for ASD likewise emphasized the principal remaining challenges concern trial design heterogeneity, small sample sizes, the predominance of open-label studies, and the gap between preclinical and clinical translation, with long-term efficacy and safety demonstration remaining among the recognized hurdles [17]. Taken together, the trial-level safety evidence spanning the UC-MSC and UCB programs—encompassing more than 350 dosed pediatric participants when the cerebral palsy cohort is included-supports an acceptable short- to medium-term safety profile across diverse cell sources, doses, and administration routes, with the most consistent observation being the absence of treatment-related serious adverse events. The principal remaining safety gap concerns long-term follow-up beyond 2 years, which has been reported in only a minority of trials and remains a recognized methodological limitation examined in the next sub-section together with efficacy signals.

### 6.4. Efficacy Signals and Methodological Limitations

The efficacy signals across pediatric ASD cell therapy trials are mixed and source-specific. UC-MSC trials have produced consistent positive signals in open-label and Phase I designs, although the one randomized, placebo-controlled trial of a UC-MSC product did not meet its primary endpoint (Section 6.1): the Lv et al. Phase I/II trial in 37 children with ASD showed statistically significant improvements on CARS, ABC, and CGI at 24 weeks in both treatment arms (CBMNC alone and combined CBMNC + UC-MSC) compared with rehabilitation-only control, with the combination arm producing larger therapeutic effects than CBMNC alone [20]. The Duke Phase I trial of UC-MSC in 12 children with ASD reported clinical improvement in six of twelve participants on at least two ASD-specific outcome measures [22]. Case-level evidence reinforces the open-label signal: a Turkish child receiving IT and IV WJ-MSC showed significant CARS and Denver Developmental Screening Test, Second edition (Denver II) improvement particularly in language and gross motor skills over 2-year follow-up [127], and an Israeli–Serbian child with severe ASD plus epilepsy receiving five sequential courses of allogeneic placenta and UC-MSC showed disappearance of seizures, normalization of EEG after the first course, and continuous improvement in ASD symptoms, social skills, and emotional expression [128]. The biological rationale for these signals-MSC immunomodulation, inflammation modulation, paracrine neurotrophic support-has been comprehensively reviewed in the context of immune dysregulation as a tractable therapeutic target in ASD [144], and a 2026 comprehensive synthesis elaborated the multi-faceted mechanisms underlying MSC efficacy in ASD: neuroinflammation modulation via anti-inflammatory cytokine and adenosine signaling pathways; neurogenesis and synaptic plasticity enhancement mediated by BDNF, GDNF, and other neurotrophic factors; gut–brain axis restoration; and cerebral angiogenesis promotion through VEGF [17].

The UCB trial efficacy landscape is more heterogeneous and ultimately mixed. The Duke Phase I open-label trial in 25 children reported significant 6-month improvements on parent-reported social communication, clinician overall severity, expressive vocabulary, and objective eye-tracking measures of social attention, with higher baseline nonverbal IQ predicting greater response [130]; subsequent same-cohort biomarker analyses identified EEG spectral shifts (increased alpha and beta, decreased theta) by 12 months [134] and DTI-detected increases in white matter connectivity in frontal, temporal, and subcortical regions [135]. The definitive Phase II randomized, placebo-controlled, double-blind trial in 180 children, however, was negative on the primary outcome (VABS-3 Socialization Domain) and on both secondary outcomes (PDD Behavior Inventory and expressive vocabulary) in the entire sample; a post hoc subanalysis in children without ID identified that allogeneic-but not autologous-UCB was associated with improvement on the clinician-rated CGI-Improvement scale, communication, attention measures, and EEG alpha and beta power, although the odds ratio for clinician-rated improvement was not statistically significant [21]. An independent placebo-controlled crossover trial in 29 children showed trends toward socialization improvement without statistical significance for any endpoint [136]. The CORDUS study reported a positive subgroup signal—78% improvement in children aged 3–7 years versus 11% in those older than 8 years or weighing more than 35 kg, with age, body weight, and inflammatory phenotype emerging as effect modifiers [131]—and the same cohort’s biomarker analysis identified NSE elevation in 40 of 41 ASD children as a candidate response biomarker [137].

Several methodological limitations constrain interpretation of these signals across the entire evidence base. Sample sizes remain small for most trials (12–37 children for UC-MSC studies; 25–180 for UCB studies), heterogeneous outcome instruments preclude direct cross-trial pooling (VABS-3 and PDD Behavior Inventory in UCB Phase II; CARS, ABC, CGI, Denver II in UC-MSC trials; Indian Scale for Assessment of Autism [ISAA], Functional Independence Measure [FIM]/WeeFIM [Functional Independence Measure for Children] in BM cell therapy trials), and follow-up durations vary from 6 months (UCB Phase II RCT) to 24 weeks-2 years (UC-MSC trials) with very few trials reporting beyond 2 years. The Phase II RCT design that produced negative primary findings for UCB has not been replicated for UC-MSC, leaving the strongest signals in this product class derived from non-randomized or single-arm Phase I designs.

Four confounders bear on the interpretation of such designs in pediatric ASD and are not controlled in most of the trials reviewed here. Placebo response is substantial in pediatric ASD intervention trials, and the negative primary outcome of the only adequately powered placebo-controlled trial in this field, alongside positive open-label signals from the same program, is consistent with a meaningful placebo contribution to the latter [21,130]. Caregiver expectancy compounds this, because the principal outcome instruments are caregiver-completed and caregivers of children enrolled in a cell therapy trial are rarely naive to the intervention; the open-label studies reporting the largest effects, including the 91% improvement rate on ISAA in an uncontrolled bone marrow mononuclear cell trial, are the designs in which this influence is least constrained [48].

Regression to the mean operates wherever children are enrolled on the basis of high baseline severity scores, so that part of the subsequent decrease in those scores is expected on repeat measurement alone; this applies to every single-arm design in the field and is not addressed by within-subject comparison. Spontaneous developmental change is the most specific concern for this indication, since these trials recruit children from two years of age into late childhood, spanning a period of rapid and heterogeneous developmental gain, and improvement on developmental or adaptive-behavior instruments over six to twenty-four months cannot be attributed to intervention without a concurrent control group; the age-dependence of response reported in CORDUS, favorable in children aged three to seven years, is consistent with a treatment effect modified by age but equally consistent with developmental trajectory contributing to the measured change [131]. Only four of the trials reviewed here included a concurrent control group, comprising the placebo-controlled UCB Phase II trial [21], the placebo-controlled UCB crossover study [136], the randomized autologous BM-MSC trial [46], and the rehabilitation-only comparator arm of the Lv trial [20], and it is notable that the two placebo-controlled studies are also the two that did not reach statistical significance on their primary endpoints. The meta-analysis of this literature accordingly qualified its pooled estimate by the small size and methodological heterogeneity of the contributing studies [129]. Mechanistic and dose-finding concerns further complicate cross-trial comparison: the Vinmec correspondence highlighted that the Phase II UCB doses (CD34^+^ 0.3 × 10^5^/kg autologous and 0.7 × 10^5^/kg allogeneic) were substantially below the 1.7 × 10^5^/kg threshold previously established by Wagner et al. (cf. Section 6.2.1) [132], that IV systemic delivery may have limited brain delivery through pulmonary, cardiac, hepatic, and renal trapping, and that 6-month follow-up was likely too short relative to the 12–18 month windows in which prior trials documented progressive improvement [138]. Comparison with non-UC cell therapies adds further heterogeneity: the autologous BM-MSC RCT in 32 children showed safety and feasibility but limited efficacy, with significant improvement on the time-by-group interaction for CGI severity alone [46], and the IT autologous BMMNC open-label trial in 32 children reported 91% improvement on ISAA but in an uncontrolled design susceptible to expectation effects [48]. Parallel challenges concern the emerging cell-free strategies under development for ASD: MSC-derived exosome programs face significant heterogeneity in exosome isolation, characterization, dosing, administration routes, production processes, and quality control that constrain meaningful cross-study comparison [23], with parallel concerns for broader secretome-based approaches—encompassing both EVs and soluble non-vesicle factors such as growth factors, chemokines, and immunomodulatory molecules [145].

The meta-analytic synthesis offered by the only dedicated meta-analysis of cell therapy for childhood ASD reflects this heterogeneous evidence base. Pooling five eligible controlled studies across multiple cell sources (BM-MSCs, mononuclear cells alone or combined with MSCs, cord blood, CBMNC combined with UC-MSCs, and autologous UCB) against rehabilitation-only reference standards identified a statistically significant reduction in CARS scores favoring stem cell intervention (WMD −5.96, 95% CI −8.87 to −3.06, *p* < 0.0001) but no significant difference on CGI (RR 1.01, 95% CI 0.87–1.18) and no significant difference in adverse-event rates—with the authors concluding that current evidence is compromised by small study sizes, lack of standardized injection routes and doses, heterogeneous diagnostic and outcome instruments, and short follow-up windows [129]. The pediatric CP program demonstrates that a dose threshold (≥2 × 10^7^ TNCC/kg in the Sun et al. crossover Phase II) can convert an overall negative primary endpoint into significant subgroup efficacy on motor function and brain connectivity, an analytic framework directly relevant to ASD trial design [139]. Resolution of these methodological gaps requires trial designs addressed in Section 9.4, in particular head-to-head UCB versus UC-MSC comparisons, which have not yet been undertaken in pediatric ASD [17]. The optimal administration route to maximize CNS delivery while preserving safety, examined in the next major section, is closely linked to several of these methodological priorities.

## 7. Administration Routes: A Practical Analysis

### 7.1. IV Administration

IV infusion is the dominant clinical administration route for cell therapy in pediatric ASD, employed in essentially every Phase I and Phase II trial of UC-MSC and UCB conducted to date. The Duke Phase I trial of UC-MSC used IV infusions of 2 × 10^6^ cells/kg at 2-month intervals in 12 children with ASD aged 4–9 years [22], and the broader UCB program—encompassing the Duke Phase I trial of 25 children [130], the Phase II randomized trial of 180 children comparing autologous and allogeneic UCB [21], the Sutter placebo-controlled crossover trial in 29 children [136], the CORDUS study in 56 children [131], and the Phase II crossover trial in 63 children with CP [139]—has uniformly adopted single or repeated IV infusion as the standard delivery modality. The Lv et al. trial combined IV CBMNC infusion with IT UC-MSC delivery in 37 children with ASD over four weekly sessions [20], representing one of the few hybrid-route protocols. Across these clinical experiences the cumulative pediatric IV exposure exceeds 350 dosed participants with consistent acceptable short- to medium-term safety, as detailed in Section 6.3.

The preclinical evidence base supporting IV administration in pediatric ASD models has grown substantially in the past two years. A 2024 study in the VPA-induced rat model of autism demonstrated that IV administration of UC-MSCs significantly alleviated microglial activation in the brain—particularly in the anterior cingulate cortex-and effectively improved sociability in VPA-exposed rats, with parallel in vitro Transwell migration assays demonstrating that UC-MSCs promoted the growth of primary-cultured cortical neurons [113]. A complementary 2024 study in the BTBR mouse model (a commonly used ASD model demonstrating robust behavioral deficits consistent with ASD diagnostic criteria, decreased BDNF signaling, and reduced hippocampal neurogenesis) [112] (cf. Section 7.4) demonstrated that IV transplantation of human BM-MSCs (hBM-MSCs) improved social deficits across the open field test, light–dark box test, novel object recognition, and free social test, reduced stereotypic behaviors, and reversed the altered Firmicutes/Bacteroidetes ratio in fecal microbiota with several specific differential taxa correlating with ASD-relevant behavioral scores [146]. Together these studies establish that IV-delivered MSCs—from both umbilical cord and BM sources—reach therapeutic targets sufficient to modulate two distinct ASD-relevant pathophysiological axes (neuroinflammation/microglial activation centrally and gut-microbiota composition peripherally) without requiring direct CNS access.

The principal advantages of the IV route are non-invasiveness relative to IT or ICV delivery, scalability for outpatient administration in pediatric ages 2–9 years, and applicability to repeated dosing regimens without anesthetic burden. The principal mechanistic limitations were articulated most directly in the Vinmec correspondence on the Dawson Phase II RCT: intravenously delivered cells may be substantially trapped in the lung, heart, liver, and kidney before reaching brain tissue, limiting the fraction of administered dose that achieves CNS exposure and potentially contributing to the failure of the Phase II primary endpoint at 6 months despite uniform safety [138]. The mechanistic rationale for IV efficacy in pediatric ASD-articulated by the Duke group in the same exchange-rests on systemic immunomodulation and paracrine effects mediated by the CD14^+^ monocyte fraction in UCB [133] (cf. Section 6.2.3) or by MSC-secreted factors (cf. Section 3.4 and Section 3.5) rather than on direct cellular CNS engraftment. Whether this paracrine paradigm is sufficient for full clinical efficacy at currently administered IV doses, or whether dose escalation or alternative routes are required, remains the principal unresolved question for IV-administered cell therapy in pediatric ASD.

The mixed Phase I and Phase II efficacy signals reviewed in Section 6.4, together with the mechanistic concerns regarding pulmonary first-pass trapping, motivate examination of routes achieving more direct neural delivery, beginning with the IT route in the next sub-section.

### 7.2. IT Administration

The IT route delivers cells directly into the CSF space, bypassing the blood–brain barrier and pulmonary first-pass entrapment that constrain IV delivery. This route has been employed in several pediatric ASD trials and forms a substantial body of evidence on cell therapy safety in pediatric neurological conditions more broadly. The most comprehensive safety analysis to date is a dedicated systematic review of 39 studies of spinal IT MSC injection across neurological conditions—including autism, multiple sclerosis, traumatic brain injury, and others—which identified no serious adverse events such as infections, clinical rejection, or tumors, with the authors concluding that properly performed spinal IT injection of MSCs is exceedingly safe [143].

In pediatric ASD specifically, IT cell delivery has been examined in four notable trials with diverse cell sources. The Lv et al. Phase I/II trial in 37 children with ASD administered four weekly stem cell infusions of CBMNC and UC-MSC via IV and IT routes at 5–7 day intervals; the Combination group received two CBMNC infusions via combined IV and IT routes followed by two IT UC-MSC injections (UC-MSC dose 1 × 10^6^/kg, administered exclusively via the IT route), while the CBMNC group received one IV CBMNC infusion followed by three IT CBMNC injections. Both treatment arms produced significantly greater CARS, ABC, and CGI improvements than rehabilitation-only control at 24 weeks, with the combination arm producing larger therapeutic effects than CBMNC alone [20]. The Sharifzadeh et al. RCT in 32 children with ASD administered autologous BM-MSCs via the IT route, demonstrating safety and feasibility with limited clinical efficacy (significant improvement on time-by-group interaction for CGI severity alone) [46]. An Indian open-label proof-of-concept trial in 32 children with ASD administered autologous BMMNCs intrathecally and reported 91% improvement on ISAA and 96% global improvement on CGI-II, though in an uncontrolled design susceptible to expectation effects, with seizures in three patients controlled by medications representing the principal safety signal observed [48]. A 2025 Turkish case report described a child receiving combined IT and IV WJ-MSC transplantation with neurorehabilitation, showing significant CARS and Denver II improvement particularly in language and gross motor skills over 2-year safety follow-up [127].

The CNS biodistribution advantage of the IT route, frequently asserted as the conceptual rationale for the approach, was directly demonstrated in a 2026 porcine study employing MRI analysis, CSF profiling, and histology after IT administration of WJ- or AD-MSCs combined with neurotrophin-releasing nanoparticles. CSF was shown to facilitate MSC migration, leading to cellular accumulation around the spinal cord and brain parenchyma without adverse effects or significant systemic alterations, with C-reactive protein levels diminished following administration of MSCs combined with neurotrophin-releasing nanoparticles suggesting an additional systemic anti-inflammatory effect [147]. Pediatric-specific IT MSC experience outside the ASD literature provides additional safety reassurance: a 2024 study reported on 14 children with hypoxic–ischemic encephalopathy or immunological encephalopathy who received single IT and IV administration of BM nucleated cells followed by multiple rounds of intrathecally administered BM-MSCs, with seizure outcomes improving in most cases (fewer seizures, reduced antiepileptic drug doses), neuropsychological and motor improvements observed, manageable toxicity (mainly mild-to-moderate early-onset adverse events), and maintained safety across the 4-year follow-up period [148].

The principal advantages of the IT route are direct CNS biodistribution bypassing pulmonary trapping (cf. Section 7.1), substantially higher local cell concentrations relative to IV delivery, and the systematic-review-level safety profile demonstrated across diverse neurological indications [143]. The principal limitations are the requirement for lumbar puncture under anesthesia in pediatric patients (with associated procedural risk and parental tolerance considerations), the technical expertise required for safe pediatric IT access, the inability to deliver repeated dosing regimens at the frequency achievable with IV infusion, and the seizure signals observed in the Sharma BMMNC trial that contrast with the absence of seizures in UC-MSC and UCB trials reviewed above and warrant continued attention in IT pediatric protocols. The IT route therefore represents a reasonable second-line consideration for pediatric ASD cases where IV administration has not produced response or where direct CNS delivery is mechanistically prioritized, with the non-invasive intranasal route examined in the next sub-section offering an alternative that combines the CNS access advantage of IT delivery with the procedural convenience of IV administration.

### 7.3. Intranasal Administration

The intranasal route represents a noninvasive strategy for direct CNS delivery, bypassing the blood–brain barrier through olfactory and trigeminal pathways that connect the nasal mucosa to the brain parenchyma. The preclinical evidence base supporting intranasal delivery of MSC-derived exosomes in autism-relevant mouse models has grown substantially since 2018, and a first-in-class human clinical trial has now been registered, positioning this route as a high-priority next-generation alternative to invasive IV and IT modalities. The mechanistic foundation has been comprehensively reviewed: MSC-EVs administered intranasally migrate through the nasal route to the CNS, actively target regions of injury or inflammation, and exert immunomodulatory, neurogenic, neuroprotective, and protein-aggregate-clearing effects—with small size and safety profile providing distinct advantages over cell transplantation in neurological disease applications [149]. A 2026 synthesis comparing administration routes for MSC and MSC-EV delivery in stroke therapy further established that intranasal delivery bypasses BBB limitations, supports multidose regimens, and sustains localized repair through angiogenesis, neurogenesis, and immunomodulation, with mechanistic engagement fine-tunable through delivery design [150].

In autism-relevant preclinical models, three independent studies provide convergent evidence for intranasal MSC-exosome efficacy across diverse cell sources and ASD mouse models. The foundational study from the Offen laboratory demonstrated that intranasal administration of MSC-exosomes derived from human BM-MSCs in BTBR mice increased male-to-male social interaction, reduced repetitive behaviors, increased male-to-female USVs, and significantly improved maternal pup retrieval behavior, with no negative symptoms detected in either BTBR or healthy C57BL controls following intranasal treatment [117]. A subsequent 2020 study using UC-MSC-exosomes demonstrated that intranasally administered UC-MSC-exosomes efficiently entered brain tissue, restored social ability, and corrected repetitive stereotyped behaviors in the VPA-induced mouse model of ASD, with therapeutic efficacy attributable at least partly to the anti-inflammatory effect of the exosomes [116]. A 2020 dose–response biodistribution study extended this paradigm to AD-MSC-exosomes across three distinct ASD mouse models-BTBR, Shank3 exon 22 deletion, and Shank3 insG3680 insertion mutation-demonstrating significant dose-dependent improvement in ASD behavioral phenotypes after intranasal administration, with brain accumulation within 72 h and reduced signal in kidneys, heart, and lungs, and with intranasal delivery proving more effective than equivalent IV administration [151].

The cell-free paradigm represented by MSC-exosome intranasal delivery aligns with broader trends in ASD therapeutics. A 2026 synthesis of MSC-derived exosome programs for ASD identified the therapeutic potential of MSC-exosomes for modulating ASD pathophysiology while highlighting persistent challenges in standardizing exosome isolation, characterization, dosing, and administration routes that constrain clinical translation [23]. A 2024 narrative review of stem cell secretomes as therapeutic agents for ASD similarly emphasized the mechanistic appeal of cell-free strategies—encompassing both EVs and soluble non-vesicle factors—for ASD intervention [145]. The most recent comprehensive synthesis of MSC-based ASD therapy identified IV, intranasal, and ICV delivery as the principal administration routes employed in preclinical models, with intranasal and IV delivery considered the most clinically feasible options [17].

Clinical translation has now begun for intranasally administered MSC-exosomes in pediatric ASD. A non-randomized, open-label Phase I/II study of UC-MSC-exosome nasal spray in children with ASD aged 3 to 7 years (NCT07243561) began recruiting in September 2025 with an estimated completion in August 2026, allocating an estimated 60 participants in parallel to active UC-MSC-exosome nasal spray or to a no-intervention control group; each of the 10 administrations delivers 40 billion exosome particles on an alternate-day schedule, with treatment-related adverse events and the Childhood Autism Rating Scale, Second Edition (CARS-2) as primary outcomes assessed to week 24, and ABC scores and serum TNF-α as secondary outcomes. This trial represents the first registered clinical translation of the intranasal MSC-exosome paradigm to pediatric ASD and will yield the first human safety and preliminary efficacy data for this administration route. The principal advantages of the intranasal route in pediatric ASD are noninvasiveness compared with IT and ICV delivery, direct CNS access bypassing the pulmonary first-pass entrapment that constrains IV efficacy (cf. Section 7.1), feasibility of repeated multidose regimens without anesthesia or procedural risk, and the favorable pediatric tolerability profile inherent to nasal drug delivery. The principal limitations are the early stage of human clinical evidence (only one registered trial), the standardization gaps in MSC-exosome manufacturing that constrain reproducibility across studies [23], the limited dose-finding data in pediatric populations, and the predominant reliance on exosomes rather than whole cells with associated implications for paracrine versus cellular mechanism profiles. The intranasal route therefore represents a high-priority next-generation administration modality for pediatric ASD cell therapy, with the most invasive direct-CNS route—ICV delivery—examined in the next sub-section for completeness of the route landscape.

### 7.4. ICV Administration

The ICV route delivers cells or cell-derived products directly into the cerebral lateral ventricles, providing the most concentrated CNS exposure of any administration modality and bypassing both the blood–brain barrier and the systemic distribution constraints of IV and intranasal routes. Although procedural invasiveness has precluded clinical translation for ASD to date—no human ICV trial in pediatric ASD has been registered—the preclinical evidence base spanning a decade of work (2015–2026) has established proof-of-concept across multiple MSC sources, ASD mouse models, and durability windows up to six months, providing foundational mechanistic insights that have informed subsequent IT, IV, and intranasal paradigms.

The foundational ICV ASD experiment in the BTBR mouse model demonstrated that ICV transplantation of human MSCs attenuated stereotypical behaviors, decreased cognitive rigidity, and improved social behavior, with concurrent biochemical changes including elevated BDNF protein levels and enhanced hippocampal neurogenesis—establishing the conceptual link between ICV MSC delivery and the BDNF–neurogenesis axis as a therapeutic mechanism for ASD-relevant behavioral deficits [112]. A subsequent study from the same laboratory directly evaluated the durability of ICV MSC therapy in BTBR mice and tested whether MSCs engineered to secrete higher amounts of neurotrophic factors (NurOwn) would augment behavioral efficacy: a single ICV transplantation of either standard MSCs or NurOwn produced improvements in social behavior that lasted six months, with NurOwn demonstrating superior advantages over standard MSCs for communication skills (one and six months post-treatment), prolonged reduction in stereotypic behavior (six months versus one month for standard MSCs), and unique improvements in cognitive flexibility not observed with MSC alone [152]. These two studies together established that ICV MSC delivery in BTBR mice produces durable, multi-domain behavioral improvement linked to BDNF-mediated neurogenesis, and that engineering of the cellular product can extend efficacy across behavioral domains.

The mechanistic resolution of ICV MSC-exosome therapy has been substantially advanced by two recent studies extending the BTBR ICV paradigm to MSC-derived exosomes and to a distinct VPA-induced model. A 2024 study using exosomes derived from human AD-MSCs in BTBR mice demonstrated that intraventricular human AD-MSC-exosome delivery promoted neurogenesis in human brain organoids, ameliorated social deficits in BTBR mice, and attenuated neuroinflammation and neuronal apoptosis in vivo, with mechanistic dissection identifying the exosomal long non-coding RNA IFNG-AS1 as the active component acting as a molecular sponge for miR-21a-3p to activate the PI3K/Akt signaling pathway and thereby promote neurogenesis-a finding confirmed by fluorescence in situ hybridization showing significantly increased IFNG-AS1 expression in the prefrontal cortex following ICV injection [118]. A 2026 study extended the ICV paradigm to whole-cell delivery in the VPA-induced mouse model, administering 5 × 10^5^ human UC-MSCs into the lateral ventricles at 5 weeks of age. Behavioral abnormalities in social interaction, repetitive behavior and anxiety were mitigated without notable adverse effects on H&E staining of major organs. Mechanistic analysis showed promotion of cortical neuronal dendritic development, restoration of IGF-1R and Akt phosphorylation, upregulation of synaptic plasticity genes (GAP-43, SYP) and anti-inflammatory IL-10, and suppression of pro-apoptotic Bax/Caspase-3 and pro-inflammatory IL-6/IL-1β, identifying the IGF-1/Akt signaling axis as a candidate mechanism for ICV cord blood-derived MSC therapy in VPA-induced ASD [114].

The principal advantages of the ICV route are the maximum CNS exposure achievable by any administration modality, the documented six-month durability of behavioral and biochemical effects [152], the mechanistic accessibility of CNS-specific molecular readouts (lncRNA expression in the prefrontal cortex, IGF-1/Akt phosphorylation, BDNF protein levels, hippocampal neurogenesis), and the demonstrated efficacy across multiple MSC sources (BM, AD, UCB) and ASD mouse models (BTBR, VPA-induced). The principal limitations are procedural invasiveness requiring intracranial access under anesthesia, infection risk inherent to ventricular catheter placement, the absence of any human ASD clinical trial of ICV MSC or MSC-exosome therapy, and the translational utility constrained by procedural burden in pediatric populations-a constraint explicitly acknowledged in the most recent comprehensive synthesis of MSC-based ASD therapy [17]. The ICV route therefore functions primarily as a foundational preclinical proof-of-concept paradigm and a benchmark for maximal therapeutic exposure, against which less invasive routes (IV, IT, intranasal) must be evaluated for comparable efficacy. The comparative analysis of these four administration routes for pediatric ASD cell therapy follows in the next sub-section.

### 7.5. Comparative Considerations for Pediatric ASD

The four administration routes examined in Section 7.1, Section 7.2, Section 7.3 and Section 7.4 represent a continuum of trade-offs between procedural invasiveness, CNS delivery efficiency, clinical maturity, and feasibility in pediatric populations, with no single route demonstrating clear superiority across all parameters (Figure 3, Table 5). Route selection governs biodistribution, mechanistic engagement, and the spectrum of accessible therapeutic effects, with IV and intranasal administration the most clinically feasible, IT delivery offering direct CSF access, and ICV delivery maximizing CNS exposure at the cost of procedural invasiveness [17,150].

The quantitative basis for these trade-offs is set out in Table 5 and in Section 7.1, Section 7.2, Section 7.3 and Section 7.4. Its principal element is the pulmonary first-pass effect, whereby the majority of intravenously infused MSCs localize to the lungs and only a very small fraction reaches the cerebral parenchyma [153], which supports the Vinmec critique that the Phase II UCB doses may have been mechanistically inadequate [138]. The IT, intranasal, and ICV routes each circumvent this constraint to a different degree and at a different procedural cost [112,114,116,117,143,147,151,152].

Pediatric ASD imposes additional constraints that further differentiate the route choices. The developmental timing of intervention is itself a critical therapeutic variable: a Nature Medicine framework on psychiatric disorder treatment emphasizes that sensitive periods in brain development-and therapeutic interventions delivered during these windows, typically before the onset of clear neurological and behavioral symptoms-may prevent or ameliorate development of specific pathologies, making early-window intervention a mechanistic priority for neurodevelopmental disorders including ASD [154]. The CORDUS study is consistent with this age-window principle for UCB cell therapy: 78% of children aged 3–7 years showed evaluation score improvement versus only 11% in children older than 8 years or weighing more than 35 kg, with age, body weight, and inflammatory phenotype emerging as candidate effect modifiers in a post hoc analysis [131]. The procedural requirements of each route interact with this developmental window: IV and intranasal delivery are feasible across the full 2–8 year window without anesthesia, IT delivery requires lumbar puncture under anesthesia (acceptable but not optimal for repeated dosing in younger children), and ICV delivery requires intracranial access that has precluded its use in pediatric ASD entirely.

The IV route is the most extensively validated modality for both UC-MSC and UCB, and its safety profile across the cumulative pediatric exposure described in Section 6.3 supports it as the default first-line route, with mechanistic optimization such as TNCC dose targeting and age stratification prioritized over route change [20,21,22,130,131,136]. The IT route represents a reasonable second-line consideration where direct CNS delivery is mechanistically prioritized or where IV trials have not produced response, with the Lv et al. combination protocol and the Kabatas WJ-MSC case providing pediatric ASD precedent. The intranasal route is under active Phase I/II clinical translation (NCT07243561) and warrants attention as a next-generation administration modality, although no efficacy data in pediatric ASD are yet available [17], and the ICV route remains a preclinical proof-of-concept paradigm whose translational utility in pediatric ASD is precluded by procedural invasiveness.

On the present evidence a stratified approach is reasonable, with IV infusion of UC-MSC or UCB as the first-line option in the 2–8 year window, intranasal MSC-exosome delivery as a non-invasive alternative pending Phase I/II readout, IT delivery reserved for selected indications, and ICV delivery confined to preclinical study. Which route best balances efficacy, safety and developmental timing remains untested (Section 9.3).

## 8. Post-Treatment Considerations

The clinical efficacy of UC-MSC therapy in pediatric ASD cannot be reduced to single-endpoint readouts or to the relatively short observation windows employed in published trials. Sustained therapeutic benefit requires a coherent framework for what to monitor after treatment, how often to monitor it, how to integrate concurrent behavioral and pharmacological interventions, how to detect emerging safety signals over years rather than months, and how to incorporate caregiver observation into the clinical decision loop. This section addresses these post-treatment considerations through five subsections: outcome monitoring tools (Section 8.1), re-treatment timing and frequency (Section 8.2), concurrent behavioral and pharmacological interventions (Section 8.3), long-term safety and follow-up (Section 8.4), and a practical framework for caregiver observation (Section 8.5).

### 8.1. Outcome Monitoring Tools

Effective post-treatment monitoring of UC-MSC therapy in pediatric ASD requires a multi-layered measurement framework that captures changes across three complementary domains: behavioral phenotypes (the visible target of intervention), neurophysiological substrates (the underlying brain function altered by therapy), and biological markers (the mechanistic readouts of cellular activity). No single instrument or biomarker captures the full spectrum of MSC therapeutic effect in ASD, and the heterogeneity of trial designs reviewed in Section 6 has been driven in significant part by inconsistent outcome measure selection across the field. The following three subsections address each measurement layer in turn, with the goal of articulating a coherent monitoring framework suitable for both clinical research protocols and routine post-treatment care (Figure 4).

#### 8.1.1. Behavioral Assessment Instruments

Behavioral assessment instruments constitute the most extensively deployed outcome measurement layer across pediatric ASD MSC trials, with the published trials reviewed in Section 6 employing a heterogeneous battery of instruments that collectively cover diagnostic severity, core ASD domains, adaptive functioning, and clinician-rated global change. The most recent comprehensive synthesis of MSC-based ASD therapy enumerated the principal instruments employed in the field: CARS and CARS-2, ABC, CGI, Autism Treatment Evaluation Checklist (ATEC), VABS-3, Social Responsiveness Scale (SRS), Repetitive Behavior Scale-Revised (RBS-R), and Adaptive Behavior Assessment System (ABAS) [17], with additional instruments including the ISAA employed in individual trials [48].

The diagnostic severity scales most consistently used across MSC ASD trials are CARS and CGI. CARS has been used as the primary outcome measure in the Lv et al. CBMNC + UC-MSC trial, the Sun et al. Phase I UC-MSC trial, and the Sharifzadeh et al. autologous BM-MSC trial [20,22,46], and CARS-2, its updated second-edition form, is a registered primary outcome of the non-randomized Phase I/II study of UC-MSC-exosome nasal spray (NCT07243561). The psychometric properties of CARS-2 have recently been validated, with high internal consistency, inter-rater and test–retest reliability, and effective discrimination between ASD and typical development [155]. CGI scales (CGI-Severity for baseline severity rating and CGI-Improvement for global change scoring) have been used across the Lv, Sharifzadeh, and Sharma trials, and have been observed to capture treatment-responsive change even when more granular behavioral subscales did not-a pattern most clearly illustrated in the Sharifzadeh BM-MSC trial where only the CGI-Severity time-by-group interaction reached statistical significance [20,46,48]. ABC has been used as a secondary outcome in the Lv trial and as the primary outcome in the Sutter UC-MSC crossover trial [20,136], providing complementary measurement of irritability, lethargy/social withdrawal, stereotypic behavior, hyperactivity, and inappropriate speech subscales.

Domain-specific scales targeting adaptive functioning, social communication, and parent-reported behavioral change have been emphasized in the more recent and methodologically advanced trials. The Duke Phase II UCB trial used the VABS-3 Socialization Domain standard score change from baseline to six months as its primary endpoint [21], and the Duke Phase II hCT-MSC trial used the mean of the Socialization and Communication subscale standard scores over the same interval (NCT04089579); neither reached statistical significance on its primary endpoint. The ATEC has been used as a parent-reported outcome measure in multiple ASD clinical contexts, with a 2018 study employing ATEC alongside the newly developed Mental Synthesis Evaluation Checklist (MSEC) in 3715 parents of ASD children—demonstrating ATEC’s utility as a parent-completed instrument and providing its Communication subscale as a validity anchor for novel parent-report tools [156]. ATEC was the primary efficacy endpoint in the CORDUS study assessment battery alongside multiple parent-reported scales [131]. The ISAA was the primary efficacy endpoint in the Sharma BMMNC trial, with 91% of children showing improvement on ISAA total scores [48].

A patient-centered conceptual model developed from interviews with ASD-affected respondents and their parents organizes outcomes into core symptoms, associated symptoms, and impacts on daily living, and identifies social communication deficits as the priority therapeutic target named by respondents themselves, providing a framework for selecting patient-centered outcome measures [157]. A 2023 systematic review of cell therapy for ASD surveyed eleven completed trials and clinical applications and catalogued the outcome instruments each employed, concluding that further research is required to establish the optimal treatment strategy [18].

The instruments reviewed above suggest a possible composition for an outcome battery rather than a required one. CARS or CARS-2 offers a diagnostic severity anchor with broad clinical familiarity, ABC provides subscale-level resolution of treatment-responsive domains, and VABS-3 captures adaptive functioning with particular sensitivity to socialization and communication; a validated parent-reported instrument such as ATEC records caregiver-observable change between visits, CGI-Improvement adds a clinician-rated global measure, and an objective patient-centered outcome aligned with the McDougall framework would align the battery with the outcomes families prioritize. Which of these should be primary rather than supporting depends on the question a given trial is designed to answer. The behavioral assessment layer provides the most clinically interpretable readouts of treatment effect, but is inherently subjective and susceptible to placebo and expectancy effects, a limitation that motivates the complementary measurement layers addressed in Section 8.1.2 (neurophysiological) and Section 8.1.3 (biological) below.

#### 8.1.2. Objective Neurophysiological Measures

The subjectivity of the behavioral instruments reviewed in Section 8.1.1 creates a need for objective measures that quantify symptomatology and treatment-induced change [158]. Three neurophysiological modalities have emerged as candidate objective biomarkers for ASD intervention trials, namely EEG, eye tracking, and neuroimaging, and next-generation MSC trial designs prioritize them as complements to behavioral instruments [17].

EEG biomarkers represent the most extensively validated objective measure layer in pediatric ASD cell therapy trials, with the Duke autologous UCB Phase I trial providing the foundational evidence. A 25-child Phase I open-label trial of autologous UCB in children aged 2–6 with ASD analyzed EEG theta, alpha, and beta power changes from baseline to 12 months post-infusion, with electrophysiological recordings taken during viewing of dynamic social and nonsocial stimuli, and demonstrated significant changes in EEG spectral characteristics by 12 months post-infusion-characterized by increased alpha and beta power and decreased theta power. Critically, higher baseline posterior EEG beta power was associated with a greater degree of improvement in social communication symptoms, highlighting the potential for an EEG biomarker to predict variation in treatment outcome [134]. The subsequent Duke Phase II UCB trial expanded the neurophysiological assessment battery to include EEG alongside eye tracking and other objective measures, with these objective metrics complementing the VABS-3 socialization primary endpoint [21]. These findings together support the use of EEG as both a treatment-responsive biomarker (capturing change after intervention) and a stratification biomarker (predicting responder phenotype from baseline) in cord-derived cell therapy trials, noting that the evidence to date comes from UCB rather than UC-MSC trials.

Eye-tracking biomarkers have advanced toward regulatory qualification for ASD clinical trial endpoints. In a multisite observational study conducted by the Autism Biomarkers Consortium for Clinical Trials (ABC-CT), the Oculomotor Index of Gaze to Human Faces (OMI) showed high acquisition rates, construct validity, six-week stability, group discrimination between ASD and typical development, and associations with symptom severity and verbal ability, on the basis of which it has been accepted into the FDA’s Biomarker Qualification program [159]. A pilot randomized trial in young children further showed that a social preference paradigm differentiated ASD from typical development and that attention during face scanning predicted language skill change at six months [160]. The Duke Phase II UCB trial directly incorporated eye tracking into its neurophysiological assessment battery alongside EEG [21].

Multimodal biosensor platforms integrating EEG, eye tracking, and additional physiological measures represent the next-generation neurophysiological framework. A multisite observational study of one such platform, combining EEG, eye tracking, electrocardiography, electrodermal activity, facial affect analysis, and actigraphy with a caregiver-reported digital phenotyping component, obtained good-quality data in the large majority of recordings, captured measurable differences between ASD and typically developing individuals that correlated with standardized symptom scales, and established the feasibility of deploying such platforms in standard clinical research settings [161].

Several of these measures are candidates for inclusion in future trial designs. EEG spectral measures, particularly posterior beta, have shown sensitivity to UC-derived cell therapy and may have stratification utility; the FDA-qualified OMI provides the eye-tracking endpoint with the clearest regulatory pathway, and multimodal biosensor platforms could integrate these with physiological measures where resources permit. Baseline characterization would allow responder phenotypes to be identified prospectively, and repeat measurement at the primary endpoint would establish whether the measure is treatment-responsive. None of these has yet been used as a primary endpoint in a pediatric ASD cell therapy trial, and their role remains to be established. The objective measurement layer addressed here complements the behavioral instruments of Section 8.1.1 and the biological and inflammatory markers addressed in Section 8.1.3 below, together comprising the three-layer outcome monitoring framework for pediatric UC-MSC ASD trials.

#### 8.1.3. Biological and Inflammatory Markers

The layers addressed in Section 8.1.1 and Section 8.1.2 capture clinically observable change but do not assay the mechanistic substrate of UC-MSC therapy. Biological and inflammatory marker measurement provides the third layer, targeting the pathways set out in Section 2 and Section 3, and thereby enables both mechanistic verification of treatment effect and stratification by baseline inflammatory phenotype.

The pro-inflammatory cytokine elevation characteristic of ASD has been quantitatively established through a comprehensive meta-analysis of 38 studies including 2487 participants (1393 patients with ASD and 1094 control subjects), demonstrating medium-magnitude elevations in plasma IFN-γ (SMD = 0.53) and serum IL-1β (SMD = 0.56), and small but significant elevations in blood IL-1β (SMD = 0.35), serum IL-6 (SMD = 0.30), and serum TNF-α (SMD = 0.31) in ASD patients compared with controls. Meta-regression analyses identified latitude as a negative moderator of IFN-γ and TNF-α effect sizes and age and gender as moderators of IL-1β effects, establishing IFN-γ, IL-1β, IL-6, and TNF-α as the four candidate peripheral biomarkers with the strongest meta-analytic support for ASD biomarker panel selection [162]. A complementary multi-marker biomarker panel study in 30 ASD children and 14 controls expanded this framework to include gut microbiota profiling and additional peripheral markers, demonstrating significantly elevated serum TNF-α, TGF-β, neurotensin, and sortilin-1 in ASD patients alongside altered gut microbiota diversity, with fecal HMGB1 correlating with gastrointestinal sign severity-supporting integration of inflammatory, neuronal, and bacterial molecular biomarkers into the post-treatment monitoring battery [163].

A prospective birth cohort measuring maternal inflammatory markers in mid-gestation found associations between individual maternal cytokines and offspring ASD symptom scores at age 7, in both directions depending on the marker, suggesting that the inflammatory profile of pediatric ASD reflects prenatal programming as well as postnatal immune status [164]. These findings have direct implications for UC-MSC therapy: children with distinct baseline inflammatory phenotypes may represent responder subpopulations for whom MSC immunomodulation produces different magnitudes of clinical benefit-a stratification hypothesis supported by the inflammatory phenotype subgroup analyses in the CORDUS UCB trial reviewed below.

Trial-based biomarker readouts of UC-MSC therapy in pediatric ASD have begun to incorporate inflammatory markers as both primary and exploratory endpoints. The registered non-randomized Phase I/II study of UC-MSC-exosome nasal spray in children with ASD aged 3 to 7 years (NCT07243561) lists serum TNF-α among its secondary outcomes alongside ABC scores, with treatment-related adverse events and CARS-2 as the primary outcomes, making it the first registered pediatric ASD cell therapy trial to include a peripheral cytokine biomarker among its pre-specified endpoints. The CORDUS UCB cell therapy study in pediatric ASD identified inflammatory phenotype-alongside age and body weight-as an effect modifier of treatment response [131]. Preclinical evidence further supports the inflammatory biomarker framework: a 2024 study of IV UC-MSC in the VPA-induced ASD rat model demonstrated mitigation of behavioral abnormalities accompanied by suppression of microglial overactivation and neuroinflammation in the anterior cingulate cortex [113], and a 2024 study of IV human BM-MSC in BTBR mice demonstrated amelioration of autism-like behaviors primarily through restoration of gut microbiota dysbiosis and modulation of the microbiota-gut–brain axis—establishing a candidate microbiota–immune biomarker axis for future trial designs [146].

A pre-treatment panel comprising serum IFN-γ, IL-1β, IL-6 and TNF-α represents the meta-analytically supported core, and TGF-β, IL-10 and MCP-1 would extend it to the anti-inflammatory and chemokine dimensions. Measuring the same panel at baseline and at the primary endpoint would test whether the proposed mechanism of action is engaged, and gut microbiota and fecal markers would be relevant where the gut–brain axis is a mechanistic priority. The CORDUS inflammatory phenotype effect modifier is the only empirical precedent for using such a panel to stratify, and it was identified post hoc, so prospective evaluation is needed before any of these markers can be treated as a selection criterion. The completed monitoring framework provides the foundation for the re-treatment timing decisions addressed in Section 8.2.

### 8.2. Re-Treatment Timing and Frequency

The published clinical trial landscape of pediatric UC-MSC and UCB therapy for ASD reflects substantial heterogeneity in dosing strategy, with protocols ranging from a single infusion at study entry to weekly, monthly, or quarterly multi-dose regimens spanning up to one year. This heterogeneity reflects both pragmatic constraints (single-dose Phase I safety designs versus multi-dose Phase II efficacy designs) and the absence of comparative trial data establishing the optimal frequency of UC-MSC re-treatment in pediatric ASD. The biological rationale for repeated cell administration has been articulated most clearly in the design of the CATO trial, the first randomized double-blind placebo-controlled trial directly comparing single versus repeated UC-MSC dosing in any indication. Its premise is that in chronic heart failure cell therapy almost all studies have used a single dose with limited therapeutic efficacy because transplanted cells disappear rapidly, so that repeated administration is needed to replace them. The IV route reviewed in Section 7.1 makes this feasible, being cheaper, simpler, safer and less invasive than transendocardial or intracoronary delivery and, most importantly, permitting multiple doses [165].

The single-dose paradigm has been employed in three of the most extensively analyzed pediatric ASD trials. The Duke Phase I autologous UCB trial in 25 children aged 2–6 administered a single IV infusion at study entry and followed children for 6 and 12 months [130]. The Duke Phase II hCT-MSC trial in 137 children aged 4–11 employed a two-dose crossover design with one group receiving a single dose of 6 × 10^6^ cells/kg at baseline followed by placebo at 6 months and the other group receiving placebo at baseline followed by 6 × 10^6^ cells/kg at 6 months, providing a within-trial comparison of treatment timing while preserving a single-active-dose architecture (NCT04089579). The Sun et al. UC-MSC Phase I trial in 12 children aged 4–9 assigned participants to one, two, or three IV infusions of 2 × 10^6^ cells/kg at two-month intervals in three sequential cohorts [22]. These early-phase pediatric ASD trials have collectively established the safety profile of UC-derived cell therapy across both autologous UCB and allogeneic UC-MSC sources but have not addressed the dose–response question, since the cohorts receiving one, two, and three doses were not designed for comparison.

The multiple-dose paradigm has been employed in five pediatric ASD trials with widely varying frequencies. The Lv et al. Phase I/II CBMNC + UC-MSC trial administered four weekly infusions over four weeks via combined IV and IT routes [20]. The Sharifzadeh et al. RCT of autologous BM-MSC employed two IT injections over four weeks [46]. The Kabatas case report described repetitive IT and IV WJ-MSC transplantation with neurorehabilitation in a 4-year-old child [127], with the commentary letter by Akhtar and Nashwan specifying that significant improvements were observed after six WJ-MSC sessions over two years of follow-up [166]. The TBS-UCMSC-ASD001 trial (NCT02192749) administered IV UC-MSC infusions every three months over the course of one year (four doses total) in 20 children aged 6 to 16, with adverse events as the registered primary outcome and ATEC, CARS, macrophage-derived chemokine (MDC), and thymus and activation-regulated chemokine (TARC) as secondary outcomes; its published report was later retracted and no results have been posted to the registry. The most recent multi-dose innovation is the non-randomized Phase I/II study of UC-MSC-exosome nasal spray in children with ASD aged 3 to 7 (NCT07243561), which administers 40 billion exosome particles per dose on an alternate-day schedule for a total of 10 administrations, with assessments to week 24—the most frequent dosing schedule registered in pediatric ASD cell therapy to date. These multi-dose protocols span weekly (Lv, Sharifzadeh), variable (Kabatas), quarterly (TBS-UCMSC), and every-other-day (NCT07243561) frequencies without head-to-head comparison.

The response durability evidence informing re-dosing decisions remains preliminary. The Duke autologous UCB Phase I EEG biomarker analysis demonstrated that significant changes in EEG spectral characteristics (increased alpha and beta power, decreased theta power) appeared by 12 months post-infusion in 25 children following a single IV infusion, providing evidence that some neurophysiological effects of a single UC-derived cell infusion are durable over at least 12 months [134]. The CORDUS UCB crossover study likewise reported durability data following the inpatient ACB infusion procedure, with the most favorable response in the younger age band described in Section 6.4 [131]. Preclinical evidence supports more durable effects of ICV MSC delivery in BTBR mice, with a single ICV transplantation of either standard MSCs or NurOwn-engineered MSCs producing behavioral improvements that lasted six months [152]. These observations collectively suggest that single-dose effects can persist for at least 6–12 months, but the central question of whether repeated dosing produces incremental benefit beyond single-dose response remains unanswered in pediatric ASD-the same question now being directly addressed by the CATO trial in adult ischemic cardiomyopathy [165].

A mechanistic basis for this durability lies in the temporal profile of MSC immunomodulation, in which a transient molecular signal can produce a lasting clinical effect. In a controlled trial of allogeneic UC-MSCs given intravenously in active rheumatoid arthritis, the immunological changes were explicitly time-resolved—a transient rise in IFN-γ preceded, within two to three weeks, an increase in IL-10 and the regulatory Treg/Th17 ratio and a fall in IL-6, yet the clinical benefit persisted for 48 weeks after a single course without repeat dosing [167]. A comparable transience of the immunological signature was reported with autologous BM-derived (not UC) MSCs given intrathecally and intravenously in progressive multiple sclerosis, where the upregulation of CD4^+^CD25^+^FoxP3^+^ Tregs and the suppression of lymphocyte proliferation returned toward baseline by six months, with greater clinical benefit in patients receiving more than two injections [168]. This dissociation between transient signaling and durable change is consistent with a “hit-and-run” mechanism in which sustained engraftment is not required: biodistribution of intrathecally injected BM-derived (not UC) MSCs in immunodeficient mice showed that, in the absence of injury, cells remained largely at the subarachnoid injection site rather than distributing to the parenchyma, the authors concluding that MSCs migrate to injured tissue but remain at the injection site when no damage is present [169], while IT UC-MSCs in a subacute incomplete spinal cord injury rodent model distributed in a manner restricted to the spinal cord, with only a fraction migrating toward the lesion [170]. Taken together, these findings indicate that the durability of a single-dose effect reflects a reset of the host immunological set-point rather than long-term cell persistence-supporting single-dose protocols with extended durability follow-up, while leaving open whether scheduled re-dosing adds incremental benefit, the question the comparative designs below are intended to resolve.

The existing pediatric ASD trial base does not establish an optimal dosing frequency, and the single versus multiple dose question has not been addressed by head-to-head comparison. Single-dose protocols with extended durability follow-up therefore remain a reasonable first step for safety characterization, while comparative multi-dose designs of the kind under way in other indications would be required to determine whether repeated dosing adds benefit; biomarker-guided re-dosing using the monitoring framework of Section 8.1 offers an alternative to fixed intervals that has not yet been evaluated prospectively. The optimization of dosing frequency will depend not only on biological response data but also on the integration of concurrent behavioral and pharmacological interventions addressed in Section 8.3 below.

### 8.3. Concurrent Behavioral and Pharmacological Interventions

Pediatric ASD intervention rarely consists of a single treatment modality. The current standard of care integrates pharmacological agents for specific symptom management, behavioral and educational therapies for core developmental targets, and increasingly, complementary and integrative approaches. Despite years of research effort, no pharmacologic agent has been identified that targets the core symptoms of ASD; the FDA-approved agents in this indication—risperidone and aripiprazole—are limited to addressing agitation and irritability rather than core deficits in social communication or restricted/repetitive behaviors, and both carry substantial adverse effect profiles including metabolic syndrome, hepatic enzyme elevation, and extrapyramidal symptoms [171]. This therapeutic landscape positions UC-MSC therapy as a potential adjunct to, rather than replacement for, existing pharmacological and behavioral interventions, making explicit consideration of concurrent treatments essential to trial design, outcome interpretation, and clinical implementation.

The pharmacological co-treatment dimension is most directly illustrated by the Sharifzadeh RCT of autologous BM-MSC in 32 children with ASD aged 5–15, in which both the intervention group (BM-MSC + rehabilitation + risperidone, *n* = 14) and the control group (rehabilitation + risperidone, *n* = 18) received risperidone as background therapy. This design rendered any observed group difference attributable to the addition of IT BM-MSC rather than to monotherapy effects, with the significant time × group interaction on the CGI-Severity of Illness scale (F = 6.719, *p* = 0.002) representing the cell therapy contribution beyond risperidone and rehabilitation alone [46]. The pharmacological framework supporting this design choice is reinforced by a meta-analysis of nine RCTs involving 552 patients (identified from 14 initial candidate trials) evaluating pharmacotherapy for restricted/repetitive behaviors in ASD, which found no significant difference between medications—including fluvoxamine, risperidone, fluoxetine, citalopram, oxytocin, N-acetylcysteine, and buspirone—and placebo in treating restricted/repetitive behaviors, with the selective serotonin reuptake inhibitor (SSRI) subgroup likewise showing no significant effect, supporting little routine use of pharmacotherapy for these core symptoms and reinforcing the rationale for investigating novel mechanisms such as UC-MSC therapy [172].

Behavioral and rehabilitation co-treatment has been more consistently incorporated into pediatric ASD MSC trials. The Lv et al. Phase I/II trial explicitly required that all enrolled subjects-across the Control, CBMNC, and Combination arms-receive the same professional sensory integration and behavioral rehabilitation therapy at the Shandong Rehabilitation Therapy Center, with no additional treatments outside the trial protocol during the study period, establishing rehabilitation therapy as the common background against which cell therapy effects were measured. The rehabilitation-only Control group itself achieved measurable improvement (13.7% reduction in CARS total score and 17.4% reduction in ABC total score over 24 weeks), while the cell-therapy-plus-rehabilitation Combination group achieved substantially greater improvement (37.9% reduction in CARS and 59.9% reduction in ABC over the same period)—quantifying the incremental benefit of cell therapy above background rehabilitation [20]. The Kabatas case report explicitly described WJ-MSC transplantation as supported by concurrent neurorehabilitation, with the commentary letter confirming that significant improvements on the CARS and Denver II developmental screening test were observed after six WJ-MSC sessions in conjunction with the rehabilitation program over two years of follow-up [127,166]. The TBS-UCMSC-ASD001 trial (NCT02192749) administered four quarterly IV UC-MSC infusions over one year in 20 children aged 6 to 16, but no results have been posted to the registry and its published report was subsequently retracted (Section 6.1), so neither its safety findings nor its concurrent intervention details can be drawn on here—a limitation that complicates cross-trial comparison of cell-therapy-specific effects. The therapeutic rationale for combined cell-plus-behavioral protocols is grounded in robust evidence: a meta-analysis of 36 RCTs of behavioral therapy (within a broader review) for ASD demonstrated that cognitive behavioral therapy (CBT) (SMD = 0.42, high-certainty evidence), the Denver model (SMD = 0.61, moderate-certainty evidence), and social skills training (SMD = 0.56, moderate-certainty evidence) significantly improved social functioning in patients with ASD, supporting the integration of behavioral therapy as a high-quality evidence-based component of multimodal ASD intervention [173].

The CORDUS UCB study extended the concurrent intervention paradigm in a different direction by employing a crossover design comparing ACB infusion against individualized combination supplements (ICS) in 56 children with ASD, with both interventions administered sequentially to the same children. This design enabled direct within-subject comparison and demonstrated that ACB yielded greater improvement than ICS in 23 of 28 children, while ICS yielded greater improvement than ACB in only 5 of 28 (*p* < 0.05), establishing ACB superiority over the dietary supplement intervention while also documenting that the integrated supplement approach itself produced measurable benefit in a meaningful subset [131]. This finding has direct implications for trial design and clinical practice: the contribution of cell therapy must be evaluated in the context of, not isolation from, the broader intervention package that pediatric ASD patients typically receive—including pharmacotherapy for irritability and aggression, structured behavioral interventions for social communication, and nutritional and complementary supports.

Concurrent interventions bear directly on the interpretation of any cell therapy signal, and several measures would make that interpretation possible. Explicit pre-specification and reporting of all concurrent pharmacological agents, behavioral interventions, and complementary approaches at baseline and throughout follow-up would allow cell therapy-specific effects to be separated from the background; preserving background regimens during the active treatment phase, as in the Sharifzadeh and Lv designs, avoids the confounding introduced by medication withdrawal while maintaining the standard of care. Factorial or stratified designs comparing cell therapy alone, cell therapy added to behavioral intervention, behavioral intervention alone, and standard care would address the question that matters most to families and clinicians, which is whether UC-MSC therapy adds value to optimized standard care rather than whether it produces effects in isolation. Whether such designs are feasible at the sample sizes currently achievable in this field is an open question. These concurrent intervention considerations directly inform the long-term safety and follow-up framework addressed in Section 8.4 below.

### 8.4. Long-Term Safety and Follow-Up

The therapeutic case for UC-MSC therapy in pediatric ASD ultimately depends on a favorable risk-benefit ratio demonstrated not only at the time of treatment but over extended follow-up sufficient to detect delayed adverse events, durability of clinical effect, and long-term developmental impact. A systematic review and meta-analysis of stem cell therapy for children with ASD identified five eligible studies and compared safety outcomes between stem cell and rehabilitation-only control groups, finding no significant difference in adverse event incidence (RR = 1.55; 95% CI 0.60–3.98; *p* = 0.36); the corresponding efficacy findings are detailed in Section 6.4. The authors explicitly noted that the evidence base is compromised by limited study size, the absence of standardized injection routes and doses, diagnostic tool heterogeneity, and—critically—the shortage of long-period follow-up studies, calling for further investigation to systematically confirm safety and efficacy [129]. This ASD-specific meta-analytic conclusion is consistent with broader MSC safety findings: a meta-analysis of MSC therapy in chronic liver disease across 12 RCTs reported fever as the only adverse event in five studies, with no serious adverse events or side effects observed [174], and a meta-analysis of IV UC-MSC therapy in coronavirus disease 2019 (COVID-19), across eight studies, demonstrated lower mortality risk with no significant association with adverse events or serious adverse events [175]. The safety profile of MSC therapy is therefore consistent across diverse clinical indications, although ASD-specific long-term data remain limited.

Short-term safety in pediatric ASD MSC trials has been uniformly favorable across all reported studies. The Lv et al. Phase I/II trial of combined CBMNC and UC-MSC transplantation in 23 children with ASD documented transient low-grade fever in five subjects (three in the CBMNC group and two in the Combination group), all resolving without medical intervention, with no allergic, immunological, or other serious adverse events observed at the time of injection or throughout the 24-week follow-up period; complete blood count, serum biochemistry, immunology parameters (Ig A/G/M, complement C3/C4, T-cell subsets), and other laboratory measures showed no significant changes from baseline [20]. The Duke Phase I autologous UCB trial in 25 children aged 2–6 documented safety over a 12-month follow-up period [130], and the Sun et al. Phase I trial of allogeneic UC-MSC in 12 children likewise demonstrated favorable short-term tolerability [22]. The Sharifzadeh autologous BM-MSC randomized trial in 32 children with ASD reported safety and feasibility as the primary conclusion across 12 months of follow-up [46].

Medium-term safety and durability data are now emerging from several pediatric ASD MSC and UCB trials. The Duke Phase II hCT-MSC trial followed 137 children aged 4–11 through the two-dose crossover design over a primary endpoint of six months with extended assessments, with 133 of 137 participants receiving a second infusion (NCT04089579). The Murias et al. EEG biomarker analysis of the Duke autologous UCB Phase I trial documented persistence of significant EEG spectral changes by 12 months post-infusion, providing evidence of biological durability following a single IV infusion [134]. The CORDUS UCB crossover study evaluated 56 children with ASD with treatment efficacy assessed at six months post-treatment, demonstrating no serious adverse reactions during or after either cord blood or supplement administration [131]. The Kabatas WJ-MSC case report represents the longest follow-up among the pediatric ASD MSC trials reviewed here, with no adverse events over two years of observation [127,166]. The registered non-randomized Phase I/II study of UC-MSC-exosome nasal spray (NCT07243561) will provide additional medium-term data over the 24-week assessment period.

Long-term safety considerations remain incompletely characterized in pediatric ASD MSC therapy. The existing trial base spans only 6–24 months of formal follow-up for most studies, with the Kabatas case report at two years representing the upper end of published observation. Theoretical long-term concerns relevant to pediatric ASD MSC therapy include the possibility of delayed tumorigenicity (mitigated for the UC-MSC product used in one pediatric ASD trial by soft agar cloning assays and tumorigenicity experiments in mice, which showed no carcinogenicity [20]), ectopic tissue differentiation following systemic infusion, development of anti-HLA or donor-specific antibodies in allogeneic recipients with implications for future allogeneic interventions, and long-term immune modulation effects in developing children. The Lv et al. trial specifically addressed product safety screening, with all cell preparations tested for sterility, mycoplasma, and endotoxin contamination, demonstrating the importance of GMP-grade manufacturing in mitigating immediate safety risks [20]. However, the pediatric ASD literature does not currently include systematic long-term safety registries or pre-specified extended follow-up protocols beyond two years post-treatment, representing a major knowledge gap that future trial designs should address.

The absence of long-term data suggests several directions for future trial design. Pre-specified follow-up extending well beyond the two years currently reported, with developmental, immunological, and oncological monitoring at defined intervals, would address the principal gap, and pediatric ASD MSC therapy registries established as collaborative multicenter efforts would capture the rare and delayed events that single-site trials are underpowered to detect. GMP-grade manufacturing with comprehensive product testing, as in the Lv protocol, remains the foundation of immediate safety. Anti-HLA antibody and immune reconstitution monitoring would be particularly relevant for children who may receive allogeneic interventions later in life, and developmental trajectory monitoring through adolescence would be needed to detect late-emerging effects. The appropriate duration and scope of such follow-up have not been established for this indication and would benefit from consensus discussion. These considerations complement the active monitoring framework reviewed in Section 8.1 and provide the foundation for the caregiver observation framework addressed in Section 8.5 below.

### 8.5. Framework for Caregiver Observation

Caregivers observe their child across natural contexts and over timeframes that clinical assessment cannot capture, and are typically the first to notice change in communication, sensory responsiveness, social engagement, sleep, gastrointestinal symptoms, or irritability after cell therapy. All five published trials reviewed in Section 6 relied substantially on caregiver-completed instruments as primary or secondary outcomes [20,21,22,46,127,130,131], which makes the framing of caregiver observation a component of trial methodology rather than a peripheral concern.

The caregiver-report instruments used in these trials, described in Section 8.1.1, span a wide diagnostic and outcome-monitoring range: Caregiver-interview measures formed the primary outcome architecture of the two Duke UCB trials, with the VABS-3 Socialization Domain the designated primary endpoint of the Phase II trial [21,130], ABC provided the secondary battery in the Lv trial with high concurrent validity against clinician CARS assessment [20], ATEC was one of three efficacy instruments in CORDUS, scored by an independent assessor after interviewing parents and therapists [131,156], GARS-II formed part of the Sharifzadeh battery [46], and Denver II documented developmental progress in the Kabatas case report [127,166]. Their convergence across trials makes caregiver report the methodological backbone of outcome characterization in this field.

The validity of caregiver-completed instruments, however, varies across demographic and cultural contexts in ways that demand explicit consideration during trial design and interpretation. An analysis of Social Communication Questionnaire (SCQ) and SRS performance in the Study to Explore Early Development (SEED) demonstrated that specificity decreased as maternal education and family income decreased, and as developmental functioning declined and behavior problems increased-indicating that false-positive rates of these widely used screeners are systematically associated with child characteristics and family demographic factors, and that clinicians should be mindful of differential instrument performance across socioeconomic and clinical subgroups [176]. A Farsi cross-cultural validation of the Social Responsiveness Scale, Second Edition (SRS-2) in Iranian school-aged children confirmed its validity as a screening instrument for social communication problems in that linguistic context [177]. Outcome instruments should therefore be selected with attention to the cultural and demographic characteristics of the trial population, using locally validated versions rather than presuming cross-cultural equivalence of the original English-language instruments.

A second consideration that has been underappreciated in pediatric ASD cell therapy trial design is the wellbeing of the caregivers themselves, who function not only as observation instruments but as primary caregivers whose mental and physical health directly shapes the child’s daily care environment. Cross-sectional studies in two countries report that caregivers of autistic children experience rejection, isolation, and work-related difficulty attributable to courtesy stigma, with below-average health outcomes concentrated in female caregivers, those aged 45 or older, and financially strained households [178], and that they report fewer social supports, reduced quality of life, and heightened anxiety, depression, and hopelessness, with hope mediating the relationship between perceived social support and resilience [179]. These findings underscore that caregiver wellbeing should be measured at trial baseline and longitudinally throughout intervention follow-up—not solely to characterize trial population characteristics, but because the trajectory of caregiver wellbeing may itself function as a meaningful trial outcome and may moderate the reliability of caregiver-reported child outcomes.

Several measures would strengthen the caregiver observation component of future trials: baseline caregiver training in what to monitor and how to document it, selection of caregiver-report instruments with documented validity in the cultural and demographic context of the trial population, inclusion of caregiver wellbeing measures both as covariates of child outcome reliability and as outcomes in their own right, systematic capture of caregiver-reported adverse events between scheduled assessments, and integration of caregiver observation with the three-layer outcome battery of Section 8.1. This framework completes the post-treatment monitoring architecture reviewed in Section 8 and leads to the challenges and future directions addressed in Section 9.

## 9. Challenges and Future Directions

### 9.1. Product Standardization

The translation of UC-derived cell therapy from preclinical promise to robust clinical efficacy in pediatric ASD has been substantially constrained by the heterogeneity of products labeled as MSCs across studies. A 2018 survey by the European Society for Blood and Marrow Transplantation (EBMT) of 17 affiliated centers manufacturing MSC for clinical use concluded that clinical studies have not yet provided conclusive evidence of efficacy largely because of the lack of patient stratification criteria, and that the heterogeneity of MSC preparations is also a major contributing factor, as manufacturing of therapeutic MSC is performed according to different protocols among different centers. The survey documented large heterogeneity in product specification, particularly in the markers used for phenotypical characterization and their threshold of expression, use of potency assays to test MSC functionality, and karyotyping, explicitly calling for harmonization to reduce variability in clinical trials and improve interpretation of results [180]. The pediatric ASD cell therapy trial landscape reviewed in Section 6 reflects exactly this concern: trials have employed allogeneic UCB mononuclear cells, autologous UCB, allogeneic UC-MSC, autologous BM-MSC, and WJ-MSC across diverse manufacturing protocols, dose ranges, and potency benchmarks, rendering cross-trial comparisons of efficacy and safety methodologically fragile. The cornerstone of MSC standardization remains the ISCT minimal criteria, which require that MSC be plastic-adherent in standard culture conditions; express CD105, CD73, and CD90 while lacking expression of CD45, CD34, CD14 or CD11b, CD79α or CD19, and HLA-DR surface molecules; and differentiate into osteoblasts, adipocytes, and chondroblasts in vitro [33]. Adherence to these criteria within pediatric ASD MSC trials has been variable but inconsistent across the literature. The Lv et al. CBMNC + UC-MSC trial reported that UC-MSCs harvested from passage 2 expressed CD29, CD73, CD90, and CD105 in ≥95% of cells, while expression of CD45, CD34, CD14, CD79, and HLA-DR was 2% or less, additionally documented capacity for adipogenic and osteogenic differentiation, and confirmed absence of carcinogenicity through soft agar cloning assay and tumorigenicity experiments in mouse models [20]-an exemplar of ISCT-compliant product characterization within an ASD trial. Beyond identity verification, the development of standardized potency assays measuring the biological activity that mediates therapeutic effect has become a regulatory priority. A bioprocess design study for clinical-grade WJ-MSC manufacturing under GMP demonstrated that a validated lymphocyte proliferation assay measuring immunomodulatory capacity was approved by the competent regulatory authority for batch release, with all WJ-MSC batches showing high capacity to inhibit the proliferation of activated lymphocytes, the bioprocess yielding batches with sufficient cell numbers for clinical use, and risk management tools enabling identification of weak points in the manufacturing chain [88]. The integration of identity (ISCT criteria), purity (microbiological safety testing), potency (immunomodulation assay), and stability (karyotype, viability) testing constitutes the contemporary framework for MSC product release in clinical trials-one that should be uniformly applied to pediatric ASD UC-MSC trial designs.

Standardization concerns extend from the cell product to the preclinical model used to justify clinical translation. The BTBR mouse, the idiopathic ASD model most commonly used to evaluate MSC therapeutic efficacy (as reviewed in Section 5), has been extensively characterized for autism-relevant phenotypes—including social affiliation deficits, communication deficits, repetitive behaviors, and anxiety-like comorbidity that broadens the model’s relevance to ASD with psychiatric comorbidity [181]. However, the reproducibility of the canonical social deficit phenotype has been demonstrated to vary substantially with the ecological validity of the testing paradigm: most studies of BTBR social behavior have been based on short dyadic interactions of often non-familiar partners placed in a novel environment, and in such stressful and variable conditions, the reproducibility of the phenotype was low. Long-lasting automated monitoring of group-housed mice in the Eco-HAB system distinguishes BTBR social repertoire from normosocial C57BL/6J controls and supports the validity of the model while emphasizing the need for more ecological testing of social behavior across all constructs of the Systems for Social Processes domain as defined by the Research Domain Criteria (RDoC) framework [182]. The implications for MSC preclinical evaluation in pediatric ASD are direct: preclinical studies establishing BTBR as a responsive model for cell therapy intervention—including the intracerebroventricular transplantation of neurotrophic factor-secreting MSC reviewed in Section 5 [152]—must increasingly incorporate ecologically valid behavioral testing paradigms, longer monitoring periods, and explicit consideration of phenotype reproducibility across testing environments to support reliable translation to clinical trial design.

Several measures would reduce the product heterogeneity described above, and we offer them as suggestions for discussion rather than as a specification. Systematic application of the ISCT minimal criteria with documented marker thresholds and trilineage verification, complete reporting of these characterizations, and inclusion of a standardized immunomodulation potency assay as a release criterion would establish comparability at the level of product identity and activity; full reporting of cell source, passage number, expansion medium, and cryopreservation status would allow cross-trial comparison at the level of manufacturing. Adoption of ecologically valid behavioral testing with extended monitoring in preclinical BTBR evaluation, and coordinated multicenter harmonization of the kind the EBMT survey called for in adult indications, would address the preclinical and consensus dimensions, respectively. Which of these should be mandatory rather than recommended is a question for the field rather than one this review can settle. The product standardization framework addressed here provides the technical foundation for the patient stratification considerations addressed in Section 9.2 below.

### 9.2. Patient Stratification

The clinical implementation of UC-MSC therapy in pediatric ASD has been hindered not only by product heterogeneity addressed in Section 9.1 but by the fundamental heterogeneity of ASD itself as a diagnostic category—a constellation of neurodevelopmental disorders characterized by core symptoms whose underlying genetic, immunological, and neurodevelopmental substrates differ substantially across affected children. A transcriptomic subtyping study of autistic probands in simplex families demonstrated that the genetic and phenotypic heterogeneity of cases complicates omics analyses but that multivariate cluster analysis of clinical Autism Diagnostic Interview-Revised (ADI-R) scores enables subtyping that greatly enhances the ability to identify differentially expressed genes and canonical pathways within phenotypic subgroups, with Weighted Gene Correlation Network Analyses identifying gene modules correlating with specific ASD traits-directly supporting a precision medicine approach to ASD [183]. The broader case for biomarker-informed patient stratification in psychiatric and neurodevelopmental clinical trials has been articulated in a 2018 perspective from neuropsychiatry, which observed that there is a timely opportunity to improve clinical trial design informed by patient biological and psychological phenotype, with the potential to reinvigorate drug development and clinical trials in psychiatry through biomarker panels evaluated in real-life cohorts, ultimately leading to new interventions and personalized medicines [184]. The pediatric ASD UC-MSC trial landscape has begun to surface specific stratification dimensions empirically through effect modifier analyses, but the field has not yet implemented a priori biomarker-guided enrollment criteria, and the dimensions discussed below are therefore candidates for prospective evaluation rather than validated selection criteria.

Age and developmental stage constitute the most well-established stratification dimension for pediatric ASD cell therapy. The CORDUS UCB crossover study identified age—alongside body weight, inflammation, and ferritin—as a candidate effect modifier of treatment response, with ACB infusion improving evaluation scores in 78% of children aged 3–7 years (*n* = 28) but only 11% of children older than 8 years or weighing more than 35 kg [131]. This finding is biologically consistent with the established framework of neurodevelopmental critical periods, where early intervention exploits postnatal neuroplasticity that progressively declines with age. However, a second critical period in adolescence has emerged as a promising target for experience-dependent remediation in ASD, providing a biological rationale for evaluating cell-derived therapies in older pediatric and adolescent populations rather than restricting trials to early childhood [185]. The implication for pediatric ASD UC-MSC trial design is bifurcated: efficacy trials prioritizing maximum response magnitude should preferentially enroll children in the 3–7 year window where CORDUS demonstrated the highest response rate, while trials investigating extended developmental windows or mechanistic durability should explicitly stratify enrollment across age bands (e.g., 3–7, 8–12, and 13–18 years) with pre-specified subgroup analyses rather than presuming uniform efficacy across pediatric ages.

Inflammatory phenotype provides a second empirically supported stratification dimension that aligns directly with the immunomodulatory mechanism of action of UC-MSC therapy reviewed in Section 2 and Section 4. The CORDUS study reported that high initial levels of inflammation and ferritin were associated with absence of improvement, and identified inflammation, ferritin, age, and body weight together as exploratory effect modifiers of the response observed [131], and the meta-analytic evidence for pro-inflammatory cytokine elevation in ASD provides quantitative benchmarks for pre-treatment inflammatory phenotyping: medium-magnitude elevations in plasma IFN-γ (SMD = 0.53) and serum IL-1β (SMD = 0.56), and small but significant elevations in serum IL-6 (SMD = 0.30) and TNF-α (SMD = 0.31) across 38 studies of 2487 participants [162]. Complementary multi-marker peripheral biomarker panels integrating serum TNF-α, TGF-β, neurotensin, sortilin-1, and gut microbiota profiling have been demonstrated to distinguish ASD children from controls and to correlate with gastrointestinal symptom severity [163], and maternal gestational inflammatory markers (MCP-1, IL-4, IL-10) have been linked to offspring ASD phenotypes, suggesting that the inflammatory profile of pediatric ASD reflects both prenatal programming and postnatal immune dysregulation amenable to stratification [164]. The operational implication is that future pediatric ASD UC-MSC trials should incorporate pre-treatment inflammatory biomarker phenotyping (minimum panel: IFN-γ, IL-1β, IL-6, TNF-α; expanded panel: TGF-β, IL-10, MCP-1) with explicit subgroup analyses comparing therapeutic response across inflammatory phenotype strata.

Two additional stratification dimensions warrant integration into future pediatric ASD UC-MSC trial designs. The neurophysiological dimension is supported by the Duke autologous UCB Phase I biomarker analysis demonstrating that higher baseline posterior EEG beta power was associated with a greater degree of improvement in social communication symptoms following IV cord blood infusion, highlighting the potential for an EEG biomarker to predict variation in treatment outcome [134]. The clinical severity dimension is supported by the CARS-2 validation study in Iran, in which separate forms were validated for standard and high-functioning presentations, with a one-factor structure for the Standard Form and a three-factor structure for the High Functioning Form, indicating that the instrument’s measurement properties themselves differ by functioning level and that severity should be accounted for in stratification of cell therapy trial cohorts [155]. The stratification dimensions above suggest, rather than establish, a design for future trials. Age-stratified enrollment with pre-specified subgroup analysis across developmental bands would test the CORDUS age signal instead of assuming it; pre-treatment inflammatory phenotyping with a core cytokine panel, baseline EEG characterization, and severity stratification using validated instruments would each generate the data needed to determine whether that dimension predicts response, building on the transcriptomic subtyping framework demonstrated in simplex family analyses [183]. None of these dimensions has yet been shown to predict response prospectively, and the value of specifying them in advance lies in replacing post hoc effect modifier discovery with a testable hypothesis rather than in any established predictive validity.

The stratification dimensions above carry an implication beyond trial design. If therapeutic response is conditioned on age band, inflammatory phenotype, and baseline neurophysiology, UC-MSC therapy is unlikely to become a uniform intervention offered to all children with an ASD diagnosis, and the diagnostic infrastructure needed to identify candidate subgroups, comprising cytokine phenotyping, quantitative EEG, and standardized severity assessment, is itself unevenly distributed. Stratified efficacy and stratified access are therefore linked. Allogeneic UC-MSC products manufactured under the GMP conditions outlined in Section 4 carry costs that are not offset by reimbursement in most health systems while the therapy remains investigational, and families seeking treatment outside clinical trials encounter direct-pay providers operating under heterogeneous oversight, where product characterization and adverse-event reporting may not meet the standards described in Section 4 and Section 6.3.

Two consequences follow for the research agenda. Recruitment strategies should be examined for whether they reproduce existing disparities in ASD diagnosis, since children who receive later diagnoses are systematically less likely to fall within the 3–7 year window identified in CORDUS as the highest-response band. The biomarker panels proposed above should be assessed not only for predictive validity but for deployability, since a stratification scheme requiring infrastructure available only in specialized centers will restrict both the evidence base and the eventual treated population to those centers. Reporting the socioeconomic and geographic composition of enrolled cohorts, which current pediatric ASD cell therapy trials generally do not, would allow this to be examined rather than assumed. These patient stratification considerations provide the framework for the optimal dosing, route, and timing analyses addressed in Section 9.3 below.

### 9.3. Optimal Dosing, Route, and Timing

Dose magnitude, administration route, and treatment timing are interrelated rather than independent decisions, and no head-to-head data establish the optimal combination for any UC-MSC product. The dose range, routes, and schedules employed in pediatric ASD are described in Section 7 and Section 8.2; the heterogeneity across them reflects the absence of comparative trial data, and Phase II designs testing alternative doses, routes, and frequencies within a single protocol would be required to resolve it.

The optimal dose question in UC-MSC therapy has been most directly addressed in indications outside ASD, with two recent Phase I trials providing critical empirical observations about dose–response relationships that should inform pediatric ASD trial design. A Phase I dose-escalation trial of UC-MSC (Cellistem) in knee osteoarthritis (OA) evaluated three sequential cohorts (low-dose 2 × 10^6^ cells, medium-dose 20 × 10^6^ cells, high-dose 80 × 10^6^ cells, intra-articularly administered) preceded by a murine OA model showing dose-efficacy response with decreased inflammatory and degenerative scores. In the clinical cohort, all doses were safe and no serious adverse events were reported, although 100% of patients in the high-dose group experienced injection-related swelling in the knee joint. Western Ontario and McMaster Universities Osteoarthritis Index (WOMAC) total outcomes demonstrated significant improvements in pain and function at 3 and 6 months in patients treated with all doses compared with baseline; however, improvements were higher in patients treated with both medium and low doses as compared with high dose, so that within the tested clinical range the largest benefit was not obtained at the highest dose, even though the accompanying murine experiments in the same report showed a dose-dependent efficacy effect [186]. A Phase I two-dose study of allogeneic WJ-MSCs (MSCTC-0010) for de novo high-risk or steroid-refractory acute graft-versus-host disease (GVHD) tested IV infusions of low-dose (2 × 10^6^/kg) versus high-dose (10 × 10^6^/kg) WJ-MSC delivered on days 0 and 7 in 10 patients, demonstrating safety at both doses with no infusion-related toxicity, no treatment-related adverse events, and no ectopic tissue formation, an overall response rate of 70%, and decreased serum REG3α biomarker that correlated with clinical response—particularly in the high-dose cohort, supporting recommendation of the 10 × 10^6^/kg dose for expanded Phase 2 randomized controlled testing [187]. The implication for pediatric ASD UC-MSC trial design is that dose–response relationships cannot be assumed monotonic, and that Phase I dose-escalation followed by Phase II head-to-head dose comparison should precede definitive efficacy trials. This priority is reinforced by a recent systematic review of 29 MSC clinical trials spanning intravenous, intrathecal, intramuscular, and intranasal routes, which concluded that route-specific dosing strategies remain undefined and that clinical variability is driven substantially by heterogeneity in cell source, dose, and delivery route [188]; that analysis, however, encompassed diverse disorders in predominantly adult populations rather than the pediatric ASD-specific, source-defined question addressed here.

Route selection interacts with dose and frequency. Of the four routes compared in Section 7, IV administration remains the most pragmatic for pediatric trials because it permits repeated dosing without anesthesia, the rationale articulated in the CATO design [165]; IT delivery provides direct CSF access but limits dosing frequency in young children [20], ICV delivery produces the most durable preclinical effects but is impractical clinically [152], and the intranasal exosome paradigm offers non-invasive repeatable access whose efficacy is not yet established [17]. Comparing routes within head-to-head designs, rather than fixing route by protocol convention, would establish the route-specific risk-benefit profile.

Timing encompasses both dose intervals and developmental timing. The single-dose and multi-dose paradigms and the durability evidence bearing on them are set out in Section 8.2; neither has been compared against the other in pediatric ASD, and the CATO trial will provide the methodological framework for that comparison in an adult indication [165]. A parallel single-versus-multi-dose Phase II trial in pediatric ASD is an essential next step. Several study designs would address these questions. Phase I dose-escalation across at least three doses would characterize dose–response relationships with attention to the non-monotonic pattern observed in the knee OA trial [186], and Phase II head-to-head comparisons within a single protocol would establish route-specific and frequency-specific risk-benefit profiles, the latter modeled on the CATO framework adapted to pediatric ASD. Adaptive designs with Bayesian dose-finding and pre-specified interim analyses would identify dose-route-timing combinations within a feasible enrollment burden, and age-stratified dosing would take account of developmental differences in pediatric pharmacokinetics and of the age-related effect modifier identified in CORDUS. Which of these should come first has not been established. These considerations inform the source-specific RCT designs addressed in Section 9.4 below.

### 9.4. Toward Source-Specific RCTs

The preceding analyses of product standardization (Section 9.1), patient stratification (Section 9.2), and optimal dosing/route/timing (Section 9.3) converge on a single methodological priority that has emerged as the central argument of this review: the field of pediatric ASD cell therapy cannot advance beyond its current investigational status without direct head-to-head RCTs that compare biologically distinct cell sources within the same protocol, using the same outcome battery, in the same patient population. The most fundamental and unresolved source-specific question in the field is whether the therapeutic effect of cord-derived cell therapy in pediatric ASD is mediated by the heterogeneous mononuclear cell fraction of UCB-containing CD34^+^ hematopoietic progenitors, CD14^+^ monocytes, lymphocytes, and other cell populations in their native proportions- or by purified MSC preparations derived from cord tissue, including WJ-MSC and expanded cord tissue cell products. The answer to this question has direct implications for product development, manufacturing strategy, regulatory pathway selection, and clinical trial design across the entire pediatric ASD cell therapy field.

Two lines of evidence reviewed earlier bear directly on this question. The Duke Letter exchange of 2021, examined in Section 6.2.3, produced the most explicit published statement that the therapeutic ingredient in cord-derived therapy is the CD14^+^ monocyte rather than the CD34^+^ hematopoietic progenitor, supported by the DUOC-01 remyelination work from the same group [38,138,140]. The Lv Phase I/II trial, described in Section 6.1, remains the only pediatric ASD trial in which a purified MSC population was added to a mononuclear cell preparation within the same protocol, and the larger reductions in CARS (37.9% versus 20.0%) and ABC (59.9% versus 38.0%) scores when UC-MSC was added to CBMNC suggest that the therapeutic effect is not adequately captured by the heterogeneous mononuclear fraction alone [20]. Neither line of evidence, however, was generated by a trial designed to compare cell sources. The Duke exchange is an argument from mechanism rather than from a source-comparison endpoint, and the Lv combination arm cannot separate the contribution of the added MSC from that of the composite preparation. The source question therefore remains open, and a purpose-built comparison is required to close it.

Beyond these two empirical signals, the pediatric ASD UC-MSC literature does not currently include any head-to-head randomized trial comparing purified UC-MSC against heterogeneous UCB in the same protocol. The clinical trials of cord-derived and comparator cell therapy in pediatric ASD reviewed in Section 6 have employed allogeneic CBMNC [20], autologous and allogeneic UCB [21,130,131], allogeneic UC-MSC [22] including the completed randomized Phase II trial reported in the registry (NCT04089579), autologous BM-MSC [46], and WJ-MSC [127,166], together with a completed single-arm trial of quarterly IV UC-MSC whose report was retracted (TBS-UCMSC-ASD001) and an ongoing study of every-other-day intranasal UC-MSC-exosomes (NCT07243561). The systematic review of cell therapies for ASD by Akat and Karaöz, which compared the quantitative results of eleven completed cell therapy applications, concluded that the optimum delivery method, cell source, processing, dose, and administration interval all remain to be determined, and that whether any of these elements influences the final treatment result is itself unresolved [18]. The absence of head-to-head source-specific RCTs—combined with the heterogeneity of outcome measures, dosing protocols, routes, and patient populations across existing trials—renders source-specific efficacy comparisons currently impossible without dedicated comparative trial designs.

The proposed source-specific RCT framework for pediatric ASD UC-MSC therapy integrates the standardization (Section 9.1), stratification (Section 9.2), and dose-route-timing optimization (Section 9.3) considerations into a coherent trial design. The minimum design requirement is a three-arm RCT comparing purified UC-MSC (manufactured to ISCT minimal criteria with validated immunomodulation potency assay [33,88]) against heterogeneous allogeneic UCB (CBMNC, TNCC-standardized as in Dawson Phase II) against placebo, in pediatric ASD patients stratified by age (3–7 vs. 8–12 years), inflammatory phenotype, and clinical severity, with a pre-specified outcome battery integrating behavioral instruments (CARS-2, VABS-3, ATEC), neurophysiological measures (EEG spectral analysis, eye-tracking OMI), and biological markers (serum IFN-γ, IL-1β, IL-6, TNF-α), administered intravenously at a Phase I-established dose with multi-dose protocols modeled on the CATO design framework [165]. The proposed source-specific three-arm RCT framework is summarized in Table 6. An expanded design would incorporate additional arms comparing alternative purified MSC products and cell-free products, enabled by adaptive platform methodology with Bayesian dose-finding and pre-specified interim analyses. Such a trial would require ISCT-compliant product standardization and validated potency assays in every comparator arm, baseline biomarker stratification using the battery of Section 8.1, and multi-center implementation to reach the sample size and outcome standardization that source-specific efficacy determination demands. The recommendations above are proposals for discussion rather than established protocols, and the central biological argument of this review, that the proposed active component of cord-derived cell therapy is the purified MSC population rather than the heterogeneous mononuclear cell fraction, remains a hypothesis that only such trials can validate or refute.

### 9.5. Limitations of the Evidence Reviewed

The limitations bearing on this literature have been noted at the points where they arise, and are drawn together here so that the boundaries of what the evidence supports are visible in one place.

The clinical evidence base comprises five published trials in pediatric ASD, with sample sizes from 12 to 180 children, together with two single-patient case reports, across cord blood mononuclear cells, cord blood, cord tissue MSC, Wharton’s jelly MSC, and bone marrow MSC. Only one of the published trials was placebo-controlled, and it was negative on its primary endpoint; the remaining trials used open-label, single-arm, or active-comparator designs in which placebo response, caregiver expectancy, regression to the mean, and spontaneous developmental change are not separated from treatment effect (Section 6.4). A second placebo-controlled trial, the randomized Phase II trial of a cord tissue MSC product in 137 children, has been completed and likewise did not meet its primary endpoint, but its results are available only as a registry posting (NCT04089579) and have not undergone peer review. Outcome instruments differ across trials to a degree that precludes quantitative pooling, follow-up rarely extends beyond two years, and no trial has compared two cord-derived products against each other. The meta-analysis of this literature qualified its own pooled estimate on these grounds [129].

The mechanistic evidence is stronger than the clinical evidence, and this asymmetry is itself a limitation. The immunomodulatory and neurotrophic properties of UC-MSC are documented largely in vitro and in animal models, and the source comparisons that distinguish UC-MSC from bone marrow- and adipose-derived MSC are laboratory comparisons of proliferation, secretome composition, and migration rather than clinical comparisons of outcome (Section 3.1). The preclinical behavioral evidence rests on rodent models that reproduce operational proxies of the human diagnostic domains rather than the disorder itself, often with routes and doses not achievable in children (Section 5.1). The neuroinflammation findings that motivate the whole approach are associations rather than established causes (Section 2).

The central proposition of this review, that the proposed active component of cord-derived therapy is the purified MSC population rather than the heterogeneous mononuclear fraction, is supported by a mechanistic argument about which fraction of cord blood is active and by one trial in which purified UC-MSC was added to a mononuclear preparation. Neither was generated by a trial designed to compare cell sources, and the proposition remains a hypothesis (Section 6.2.3 and Section 9.4).

Product heterogeneity limits what can be inferred across trials even where designs are comparable, since donor variation, isolation method, passage number, culture medium, and cryopreservation each alter the final product and are inconsistently reported (Section 3.6). Long-term safety data are correspondingly thin: short- and medium-term safety is the most consistent finding in this literature, but follow-up beyond two years exists for only a minority of participants, and the developmental horizon relevant to a treated three-year-old extends far beyond any reported observation window (Section 6.3).

Finally, the recommendations in Section 9.1, Section 9.2, Section 9.3 and Section 9.4 are proposals rather than validated procedures. No stratification dimension discussed there has been shown prospectively to predict response, no potency assay has been qualified as a surrogate for clinical effect in this indication, and the trial design proposed in Section 9.4 has not been conducted. What the present evidence supports is that cord-derived cell therapy is safe enough in the short term to justify controlled comparison, and that the source question is worth designing a trial around. It does not yet support a conclusion about efficacy.

## 10. Conclusions

UC-MSC therapy is a biologically rational candidate intervention for pediatric ASD, supported by the alignment between the immunomodulatory, anti-inflammatory, and neurotrophic properties of UC-MSC and the chronic neuroinflammation, peripheral immune dysregulation, and microglial overactivation associated with ASD pathophysiology. The source-specific case advanced in this review rests on three lines of evidence: the identification of CD14^+^ monocytes as the proposed active component of cord blood in the 2021 Duke Letter exchange [38], the preclinical demonstration that DUOC-01 accelerates brain remyelination [140], and the only pediatric trial in which a purified MSC population was added to a mononuclear preparation within the same protocol, where the addition of UC-MSC to CBMNC was accompanied by larger reductions in CARS and ABC scores than CBMNC alone [20]. None of these were generated by a trial designed to compare cell sources, and the proposition that the purified MSC population is the active component therefore remains a hypothesis rather than a demonstrated conclusion.

The published clinical evidence, comprising five trials spanning allogeneic CBMNC, autologous and allogeneic UCB, UC-MSC, autologous BM-MSC, and WJ-MSC across heterogeneous protocols, has established short-term safety as its most consistent finding while leaving efficacy unresolved, constrained by small samples, single-arm designs, absent placebo controls in all but one study, and the lack of head-to-head source comparison [129]. The candidate effect modifiers reported post hoc in CORDUS, namely high baseline inflammation, ferritin, age, and body weight, suggest that any future efficacy signal may prove conditional rather than uniform [131]. These constraints, set out in Section 9.5, define what the present evidence base can and cannot support.

The methodological framework developed across Section 7, Section 8 and Section 9 is intended as a basis for discussion rather than a set of established procedures. Administration route selection, the three-layer outcome monitoring framework, product standardization anchored by ISCT minimal criteria and validated potency assays [33,88], and biomarker-guided patient stratification together define what a source-specific comparison would require. The proposed three-arm design comparing purified UC-MSC against heterogeneous allogeneic UCB against placebo, stratified by age and inflammatory phenotype and adapted from precedents such as the CATO dosing framework [165], is offered as one route to that comparison. Whether UC-MSC therapy has disease-modifying value in pediatric ASD cannot be determined from the evidence now available, and the question will be settled by trials of this kind rather than by further mechanistic argument.

## Figures and Tables

**Figure 1:**
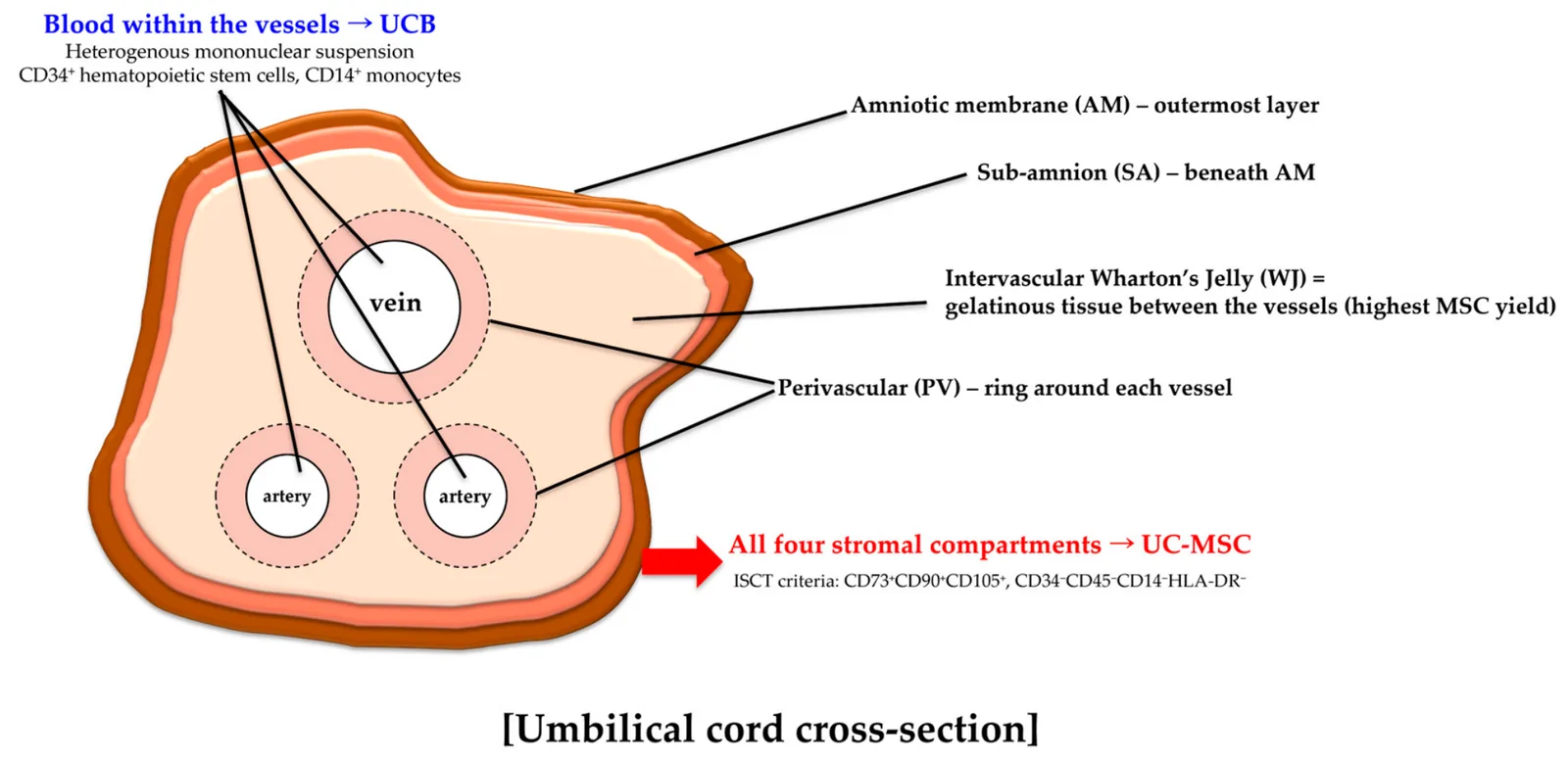
Anatomical organization of the umbilical cord and its two distinct cellular sources. Cross-section of the term umbilical cord (mean length approximately 55 cm). Mesenchymal stromal cells occupy four anatomically defined compartments, from outer to inner: the amniotic membrane (AM), the sub-amnion (SA), the intervascular Wharton’s jelly (WJ), and the perivascular (PV) region surrounding the vessels. The intervascular WJ, the gelatinous tissue between the vessels, occupies the largest volume and yields the highest number of MSCs without prior culture, whereas the AM, SA, and PV compartments require enzymatic digestion or explant culture for adequate recovery. The cord contains two arteries and one larger vein. It yields two biologically distinct cellular sources: the blood within the vessels (UCB), a heterogeneous CD34^+^/CD14^+^ mononuclear suspension, and the four stromal compartments, which provide UC-MSCs meeting the International Society for Cellular Therapy (ISCT) criteria (CD73^+^CD90^+^CD105^+^, CD34^−^CD45^−^CD14^−^HLA-DR^−^). This anatomical separation underlies the compartment biology developed in Section 3.3 and the divergent composition, banking, and mechanism developed in Section 6.2. AM, amniotic membrane; CD, cluster of differentiation; HLA-DR, human leukocyte antigen–DR isotype; ISCT, International Society for Cellular Therapy; MSC, mesenchymal stromal cell; PV, perivascular; SA, sub-amnion; UCB, umbilical cord blood; UC-MSC, umbilical cord-derived mesenchymal stem cell.

**Figure 2:**
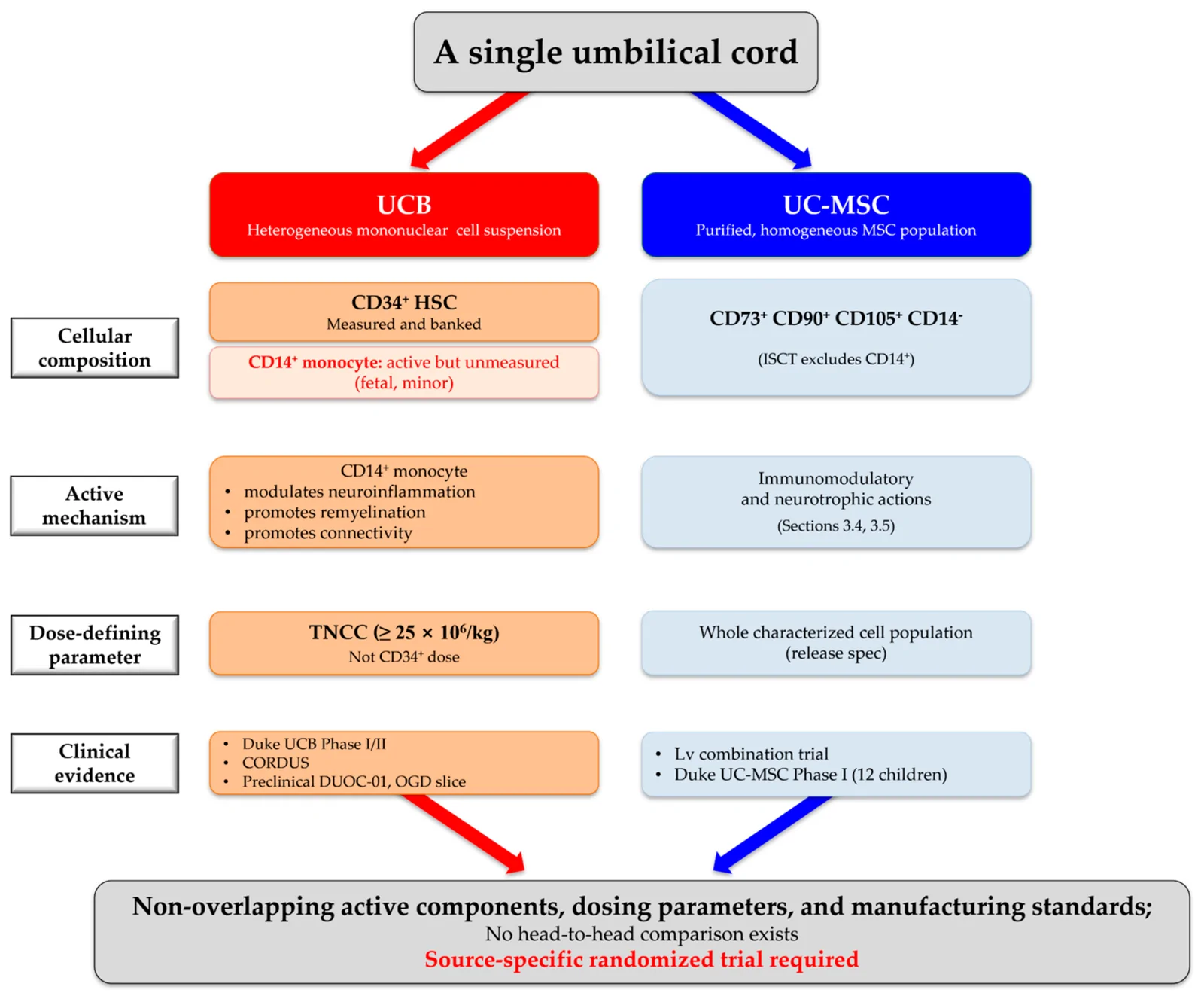
Two distinct cellular therapies from a single umbilical cord. Umbilical cord blood (UCB) is a heterogeneous mononuclear suspension whose proposed active component in neurological indications is the minor, unmeasured CD14^+^ monocyte fraction rather than the dominant CD34^+^ hematopoietic stem cell measured by cord blood banks. UCB units are therefore dosed by total nucleated cell count (TNCC). UC-MSCs are a purified population meeting International Society for Cellular Therapy (ISCT) criteria (CD73^+^CD90^+^CD105^+^) that excludes CD14^+^ cells, and act through distinct immunomodulatory and neurotrophic mechanisms. The two products thus have non-overlapping candidate active components, dosing parameters, and manufacturing standards. CD, cluster of differentiation; HSC, hematopoietic stem cell; ISCT, International Society for Cellular Therapy; TNCC, total nucleated cell count; UCB, umbilical cord blood; UC-MSC, umbilical cord-derived mesenchymal stem cell.

**Figure 3:**
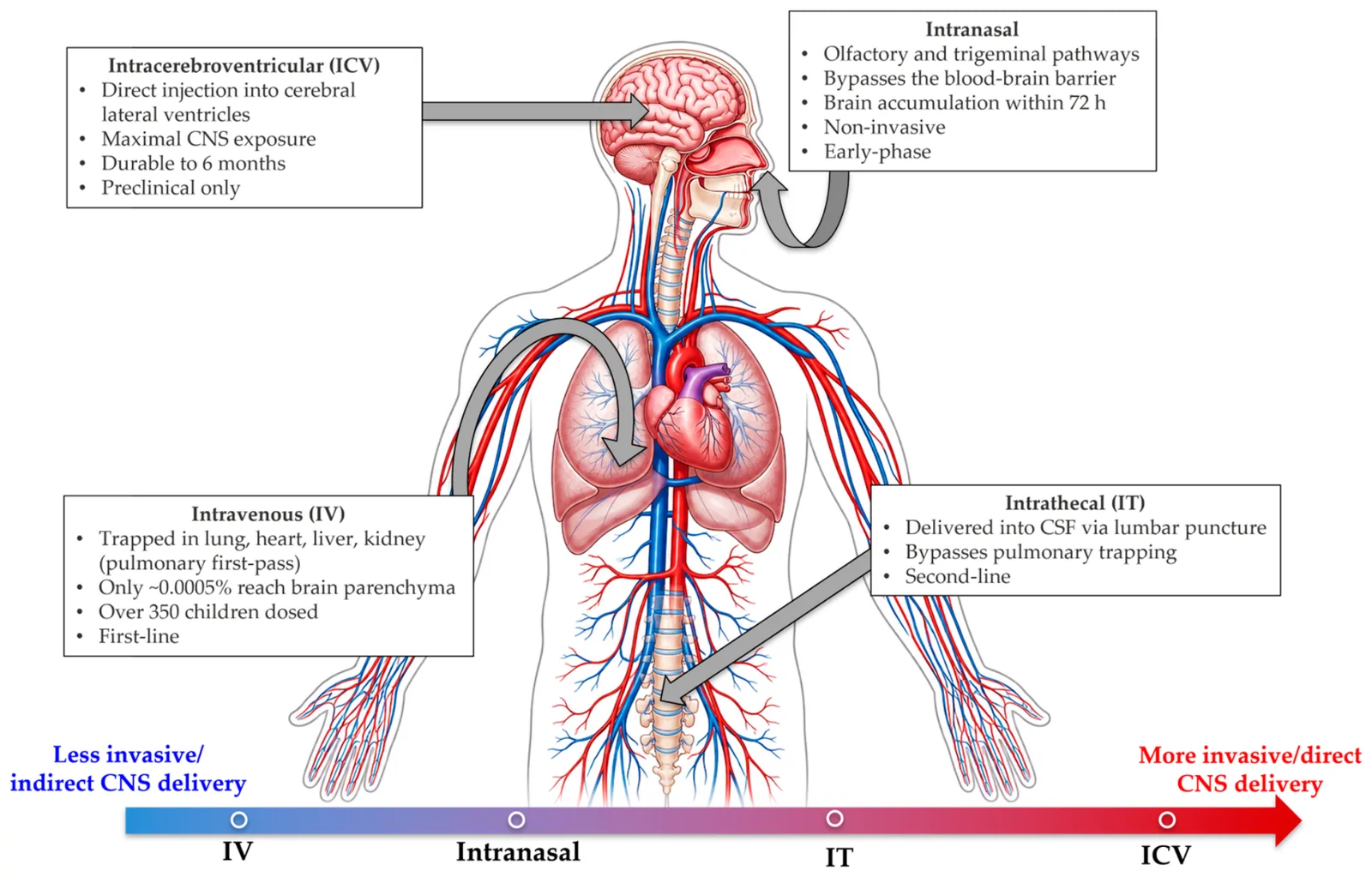
Administration routes for cell therapy in pediatric autism spectrum disorder, ordered by invasiveness and directness of central nervous system (CNS) delivery. Intravenous (IV) infusion is the most validated route but is limited by pulmonary first-pass trapping, with only a small fraction of infused cells reaching the brain. Intranasal delivery reaches the brain through olfactory and trigeminal pathways without crossing the blood–brain barrier. Intrathecal (IT) delivery places cells into the cerebrospinal fluid, and intracerebroventricular (ICV) delivery injects directly into the cerebral ventricles for maximal CNS exposure but remains preclinical. No single route is superior across all parameters. The anatomical illustration of the body and internal organs was generated with GPAI: AI STEM Copilot (Pro plan; web-based version, Turing Co., Ltd., San Francisco, CA, USA; https://gpai.app, accessed on 10 July 2026); all arrows, boxes, and text labels were produced by the authors. BBB, blood–brain barrier; CNS, central nervous system; CSF, cerebrospinal fluid; ICV, intracerebroventricular; IT, intrathecal; IV, intravenous; MSC, mesenchymal stem cell.

**Figure 4:**
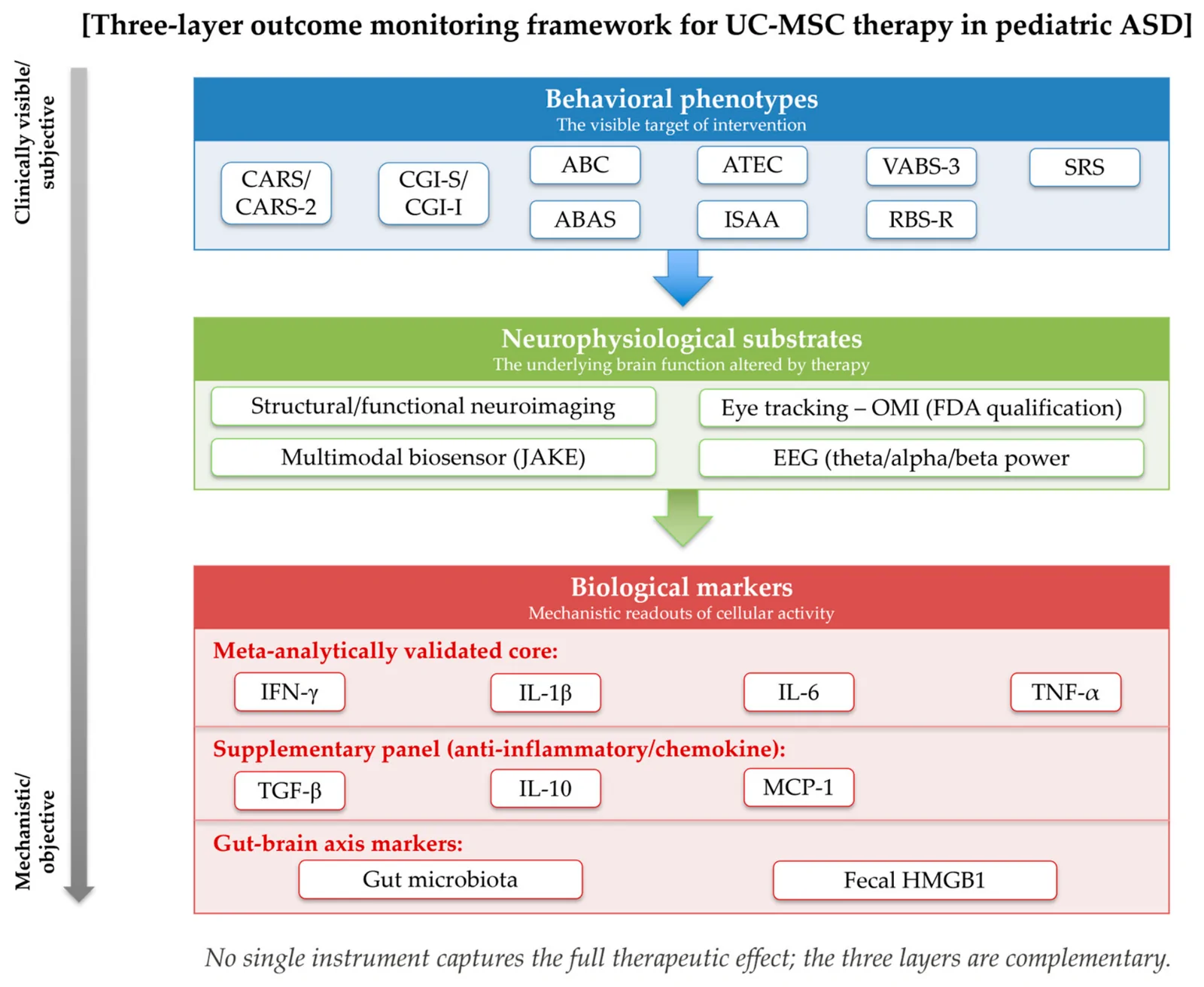
Three-layer framework for monitoring outcomes of umbilical cord-derived mesenchymal stem cell (UC-MSC) therapy in pediatric autism spectrum disorder (ASD). Behavioral phenotypes provide the visible target of intervention, neurophysiological substrates capture the underlying brain function altered by therapy, and biological markers give mechanistic readouts of cellular activity. Within the biological layer, IFN-γ, IL-1β, IL-6, and TNF-α form the meta-analytically validated core panel, supplemented by TGF-β, IL-10, and MCP-1 to capture anti-inflammatory and chemokine dimensions, with gut microbiota and fecal markers included where the gut–brain axis is a mechanistic priority. No single instrument captures the full therapeutic effect; the three layers are complementary. ABAS, Adaptive Behavior Assessment System; ABC, Aberrant Behavior Checklist; ASD, autism spectrum disorder; ATEC, Autism Treatment Evaluation Checklist; CARS, Childhood Autism Rating Scale; CGI-I, Clinical Global Impression-Improvement; CGI-S, Clinical Global Impression-Severity; EEG, electroencephalography; HMGB1, high mobility group box 1; IFN-γ, interferon gamma; IL, interleukin; ISAA, Indian Scale for Assessment of Autism; JAKE, Janssen Autism Knowledge Engine; MCP-1, monocyte chemoattractant protein-1; OMI, Oculomotor Index of Gaze to Human Faces; RBS-R, Repetitive Behavior Scale-Revised; SRS, Social Responsiveness Scale; TGF-β, transforming growth factor beta; TNF-α, tumor necrosis factor alpha; UC-MSC, umbilical cord-derived mesenchymal stem cell; VABS-3, Vineland Adaptive Behavior Scales Third Edition.

**Table 1:** Comparison of mesenchymal stem cell sources and umbilical cord blood as candidate products for pediatric ASD cell therapy. AD-MSC, adipose tissue-derived mesenchymal stem cell; ASD, autism spectrum disorder; BDNF, brain-derived neurotrophic factor; BM-MSC, bone marrow-derived mesenchymal stem cell; BMMNC, bone marrow mononuclear cell; CD, cluster of differentiation; FGF, fibroblast growth factor; HGF, hepatocyte growth factor; HLA, human leukocyte antigen; HSC, hematopoietic stem cell; ISCT, International Society for Cellular Therapy; NGF, nerve growth factor; TNCC, total nucleated cell count; UCB, umbilical cord blood; UC-MSC, umbilical cord-derived mesenchymal stem cell; WJ, Wharton’s jelly.

Property	BM-MSC	AD-MSC	UC-MSC (WJ)	UCB	Refs.
Cell identity	ISCT-defined MSC	ISCT-defined MSC	ISCT-defined MSC	Heterogeneous mononuclear fraction; CD34^+^ HSC, CD14^+^ monocytes, lymphocytes; MSC content minimal	[33,38]
Procurement	Posterior iliac crest aspiration; general anesthesia or deep sedation in children	Lipoaspiration; in children feasible essentially only during a concurrent surgical procedure	Collected from the umbilical cord discarded at delivery; no donor procedure	Cord blood collected from the umbilical vein at delivery	[39,40]
Procurement risk to donor	Severe adverse event 1.7%, respiratory complication 1.1% in 915 pediatric bone marrow aspiration and lumbar puncture procedures; rare infectious complications	Surgical and anesthetic risk of the primary procedure	None to donor	None to donor	[41,42]
Donor-age dependence	Proliferative and differentiation capacity decline with donor age	Not characterized in the studies reviewed here	Neonatal tissue; not applicable	Neonatal tissue; not applicable	[43]
Proliferative capacity	Cumulative population doubling 6 at passage 3; doubling time approximately 99 h	Between BM-MSC and WJ-MSC	Cumulative population doubling 12.3 at passage 3; doubling time approximately 21 h	Not expanded; administered as collected	[36]
Neurotrophic secretome	Lowest neurotrophic factor content of the three sources	Intermediate between BM-MSC and WJ-MSC	Highest HGF, FGF-2, NGF and BDNF	Not characterized as a secretome product	[36]
Immunomodulatory profile	Highest of the three sources under clinically relevant culture conditions	Lower immunomodulatory activity than BM-MSC	Lower than BM-MSC in functional immunosuppression under clinically relevant culture conditions; superior migration toward activated lymphocytes	Activity attributed to the CD14^+^ monocyte fraction	[36,37,38]
Dose-defining parameter	Cell number per kg of characterized population	Cell number per kg of characterized population	Cell number per kg of characterized population	TNCC and CD34^+^ content; CD14^+^ content not measured	[38]
Autologous use in ASD	Requires invasive harvest from the affected child	Patient-derived cells may carry the immune dysregulation of ASD; no comparative data	Not applicable; used allogeneically	Requires a privately banked unit	[44]
Allogeneic use	Established	Established	Established; class I anti-HLA antibodies in 5 of 12 children, clinically silent	Established	[22]
Clinical evidence in pediatric ASD	One randomized controlled trial, negative on between-group comparison; one open-label BMMNC trial	One completed trial (NCT05602116, *n* = 10); one withdrawn (NCT01502488)	One Phase I open-label trial (*n* = 12); one completed randomized placebo-controlled Phase II trial (*n* = 137), negative on its primary endpoint, reported in the registry (NCT04089579); one combination arm within a controlled trial	Phase I and Phase II trials including a placebo-controlled trial negative on its primary endpoint	[20,21,22,45,46,47,48]

**Table 2:** ASD animal models used in MSC research: model features, MSC/cell source, delivery route, and reported effects. Akt, protein kinase B; Bax, Bcl-2-associated X protein; BDNF, brain-derived neurotrophic factor; BM-MSC, bone marrow-derived mesenchymal stem cell; Caspase-3, cysteine-aspartic protease-3; EV, extracellular vesicle; GABA, gamma-aminobutyric acid; GAP-43, growth-associated protein 43; ICV, intracerebroventricular; IGF-1R, insulin-like growth factor-1 receptor; IL, interleukin; IV, intravenous; UC-MSC, umbilical cord-derived mesenchymal stem cell; VPA, valproic acid.

Model	Model Basis/ASD Relevance	MSC Source & Route	Key Reported Effects	Refs.
BTBR T + Itpr3tf/J	Inbred strain; spontaneous social communication deficits and repetitive behaviors consistent with idiopathic ASD	BM-MSCs; ICV	Reduced stereotypy, decreased cognitive rigidity, improved social behavior; elevated hippocampal BDNF and increased neurogenesis	[112]
VPA-induced	Prenatal exposure to the antiepileptic teratogen; reproduces social and repetitive deficits with neuroinflammatory and synaptic changes	UC-MSCs (IV); cord blood-derived MSCs (ICV); UC-MSC + EVs (systemic); UC-MSC exosomes (intranasal)	IV UC-MSCs improved sociability, attenuated anterior cingulate microglial activation; ICV rescue via IGF-1R/Akt, GAP-43/synaptophysin, IL-10 up, Bax/Caspase-3 and IL-6/IL-1β down; UC-MSC + EV normalized cytokines, oxidative stress, synaptic/mitochondrial ultrastructure, gut microbiota; intranasal exosomes recapitulated benefits	[113,114,115,116]
Shank3B knockout	Monogenic synaptopathy underlying Phelan–McDermid syndrome (≈75% meet ASD criteria)	BM-MSC-derived exosomes; intranasal	Improved social behavior, vocalization, repetitive behaviors; elevated prefrontal GABA receptor subunit mRNA	[111]

**Table 3:** Clinical studies of umbilical cord-derived and comparator MSC/cell therapies in pediatric ASD. ABC, aberrant Behavior Checklist; ASD, autism spectrum disorder; BM-MSC, bone marrow-derived mesenchymal stem cell; CARS, Childhood Autism Rating Scale; CBMNC, cord blood mononuclear cell; CGI, Clinical Global Impression; EEG, electroencephalography; GARS-II, Gilliam Autism Rating Scale, Second Edition; HLA, human leukocyte antigen; IT, intrathecal; IV, intravenous; RR, relative risk; UCB, umbilical cord blood; UC-MSC, umbilical cord-derived mesenchymal stem cell; WJ-MSC, Wharton’s jelly-derived mesenchymal stem cell. A 2019 Phase I/II UC-MSC study was subsequently retracted and is therefore excluded from this table.

Study	Design/*n*/Age	Cell Product & Route	Dose/Schedule	Key Outcomes & Safety	Refs.
Lv et al. 2013 (NCT01343511)	Non-randomized, open-label, single-center Phase I/II (China); 37 children, 3 arms	CBMNC ± UC-MSC + rehabilitation; IV and IT	Four infusions at weekly intervals	Both treatment arms improved CARS, ABC, CGI at 24 wk vs. rehabilitation-only; combination > CBMNC alone; no severe adverse events (37 participants)	[20]
Sun et al. 2020 (Duke)	Phase I open-label single-arm; 12 children, 4–9 yr	Allogeneic third-party-donor UC-MSCs; IV	1, 2, or 3 infusions of 2 × 10^6^ cells/kg at 2-month intervals	Well tolerated overall; agitation during IV placement in some participants; two product-related infusion events (hypersensitivity with mild hypotension after omitted premedication; moderate hypotension resolving with IV fluids); 66 non-serious events not attributed to product in 11/12; 5/12 developed clinically silent class I anti-HLA antibodies; 6/12 improved on ≥2 ASD measures	[22]
Kabatas et al. 2025	Case report; 1 child	WJ-MSCs; IT and IV, with neurorehabilitation	Not specified	Significant improvement on CARS and Denver II (language, gross motor); no serious adverse events over 2-yr follow-up	[127]
Gesundheit et al. 2025	Case report; 1 boy with severe ASD, epilepsy, abnormal EEG, ages 5.75–9 yr	Allogeneic unrelated-donor placenta- and umbilical cord-tissue MSCs	Five courses	Seizure disappearance, EEG normalization after first course, continued improvement in ASD symptoms, social skills, emotional expression	[128]
Sharifzadeh et al. 2021	Randomized controlled trial (Iran); 32 children	Autologous BM-MSCs (non-UC comparator); IT	Two IT injections over four weeks	No significant between-group difference in CARS, GARS-II, CGI over 12 mo; significant time-by-group interaction favoring intervention on CGI severity	[46]
Qu et al. 2022 (meta-analysis)	Systematic review and meta-analysis; 5 eligible studies (BM, UCB, UC-derived)	Pooled vs. rehabilitation-therapy reference	-	No significant difference in adverse-event rate (RR 1.55; 95% CI 0.60–3.98); evidence limited by small size, heterogeneous routes/doses, short follow-up	[129]
Duke Phase II (NCT04089579), results posted 2024; not yet published	Randomized, quadruple-masked, placebo-controlled two-period crossover (USA); 137 children, 4–11 yr	Allogeneic third-party-donor hCT-MSC; IV	Single dose 6 × 10^6^ cells/kg at baseline or at 6 mo, with placebo at the alternate time point	Primary endpoint (mean VABS-3 Socialization and Communication subscale change to 6 mo) not met (net difference 1.32; *p* = 0.35); Socialization *p* = 0.12, Communication *p* = 0.83; post hoc Socialization responder rate 55% vs. 35% (RR 1.57; *p* = 0.02); 1 serious adverse event in 68 treated in the first period	NCT04089579
Dawson et al. 2017 (Duke)	Phase I open-label single-arm; 25 children, median 4.6 yr (range 2.26–5.97)	Autologous UCB; IV	Single infusion	Safe and well tolerated over 12 months; improvements on parent-report social communication measures, clinician ratings, expressive vocabulary, and eye-tracking attention to social stimuli, identified as candidate endpoints	[130]
Dawson et al. 2020 (Duke)	Phase II randomized, placebo-controlled, double-blind; 180 children, 2–7 yr	Autologous (*n* = 56) or allogeneic (*n* = 63) UCB vs. placebo (*n* = 61); IV	Single infusion	Safe and well tolerated; no improvement on the primary endpoint (VABS-3 Socialization Domain) or secondary endpoints in the whole sample; post hoc subanalysis in children without intellectual disability favored allogeneic UCB on clinician-rated improvement, communication, attention, and EEG alpha and beta power	[21]
Stancioiu et al. 2025 (CORDUS)	Crossover, active-comparator (individualized supplements, stated as non-placebo); 56 children	Autologous cord blood; IV, inpatient	Single infusion per period	No serious adverse reactions; improvement in 78% of children aged 3–7 yr (*n* = 28) versus 11% of those older than 8 yr or over 35 kg (*n* = 28); high baseline inflammation and ferritin, age, and body weight associated with lesser improvement in a post hoc analysis	[131]

**Table 4:** Biological and cellular distinctions between UCB and UC-MSCs. HLA, human leukocyte antigen; HLA-DR, human leukocyte antigen-DR isotype; MSC, mesenchymal stem cell; TNCC, total nucleated cell count; UC-MSC, umbilical cord-derived mesenchymal stem cell; UCB, umbilical cord blood. The CD14^+^ monocyte mechanism and the CD34^+^/CD14^+^ therapeutic-ingredient debate are developed in Figure 2 and Section 6.2.3.

Attribute	UCB	UC-MSCs
Product class	Heterogeneous mononuclear cell suspension [133]	Purified, characterized single-population MSC product [33,51]
Anatomical source	Blood contained within the umbilical vessels [132]	Connective tissue of the umbilical cord (mean length ≈ 55 cm, diameter 14.42 mm); four stromal compartments—amniotic membrane, sub-amnion, Wharton’s jelly, and perivascular region [51,52]
Cellular composition	CD34^+^ hematopoietic stem cells, CD14^+^ monocytes, lymphocytes, granulocyte precursors, and a very small proportion of MSCs [132,133]	Wharton’s jelly yields the highest MSC number without culture (≈4.61 × 10^6^ cells/cm) [51]; CD24, CD108, CD40 discriminate genuine MSCs from contaminants [53]
Defining markers	No single defining phenotype (composite population) [133]	CD105^+^, CD73^+^, CD90^+^; negative for CD45, CD34, CD14 (or CD11b), CD79α (or CD19), and HLA-DR [33,52]
Dose-defining parameter	TNCC and CD34^+^ content; transplant standard ≥ 1.7 × 10^5^ CD34^+^ cells/kg [132]	Characterized MSC number per kilogram; release governed at whole-population level [33]
Standard release testing	TNCC and CD34^+^ quantified; CD14^+^ monocyte content not routinely measured [38]	Identity, purity, and potency of the entire characterized population [33]
Proposed active component	CD14^+^ monocytes (fetal-origin; adult peripheral-blood CD14^+^ monocytes lack this activity) [38,133]	Purified mesenchymal population acting through immunomodulatory and neurotrophic mechanisms [33]
Nomenclature caveat	Standardized cord blood banking terminology (TNCC/CD34-based) [132]	Lack of consensus on cord anatomical nomenclature; the same nominal source may denote different populations across studies [47]

**Table 5:** Comparative features of the four administration routes for MSC/cell therapy in pediatric ASD. ASD, autism spectrum disorder; BTBR, BTBR T + Itpr3tf/J inbred mouse strain; CBMNC, cord blood mononuclear cell; CNS, central nervous system; CSF, cerebrospinal fluid; EV, extracellular vesicle; ICV, intracerebroventricular; IT, intrathecal; IV, intravenous; MSC, mesenchymal stem cell; TBI, traumatic brain injury; UCB, umbilical cord blood; UC-MSC, umbilical cord-derived mesenchymal stem cell; VPA, valproic acid.

Route	CNS Delivery Mechanism	Biodistribution /key Evidence	Clinical Validation in ASD	Pediatric Feasibility (2–8 yr)	Ref.
IV	Systemic circulation; indirect CNS access after pulmonary passage	Majority of infused MSCs trapped in lungs by 48 h; only 1.5–3.7% reach arterial circulation (rat TBI model)	Most extensively validated; 339 dosed children in ASD trials (Duke Phase I/II, Sun UC-MSC, Lv CBMNC + UC-MSC, Sutter, CORDUS), rising above 350 when the Duke cerebral palsy cohort is included	Feasible across full age window; least invasive parenteral option	[20,21,22,130,131,136,138,153]
IT	Direct delivery into CSF, bypassing pulmonary trapping	Porcine data confirm CSF-facilitated MSC migration to spinal cord and brain parenchyma without systemic alteration; systematic review reports no serious adverse events	Second-line; used in Sharifzadeh BM-MSC RCT and combined-route trials	Requires lumbar puncture/sedation; more invasive in young children	[143,147]
Intranasal	Noninvasive direct CNS access via olfactory and trigeminal pathways	Brain accumulation within 72 h; supportive efficacy across multiple ASD mouse models	Early-phase; active Phase I/II translation underway (NCT07243561, UC-MSC-exosomes)	Feasible across full age window; noninvasive, repeatable	[17,116,117,150,151]
ICV	Direct injection into cerebral ventricles; maximum CNS concentration	Most durable behavioral effects (six-month duration in BTBR studies); foundational ICV paradigm; cord blood-derived MSC extension to VPA model	Preclinical only; no pediatric ASD clinical use	Neurosurgically invasive; not suitable for routine pediatric ASD	[112,114,152]

**Table 6:** Proposed source-specific three-arm randomized controlled trial framework for pediatric ASD cord-derived cell therapy. Product specifications are given for the two active arms; the placebo arm receives an excipient-matched vehicle infusion identical in appearance and volume. ASD, autism spectrum disorder; ATEC, Autism Treatment Evaluation Checklist; BM-MSC, bone marrow-derived mesenchymal stem cell; CARS-2, Childhood Autism Rating Scale, Second Edition; CBMNC, cord blood mononuclear cell; EEG, electroencephalography; IFN-γ, interferon-gamma; IL, interleukin; ISCT, International Society for Cellular Therapy; OMI, Oculomotor Index; RCT, randomized controlled trial; TNCC, total nucleated cell count; TNF-α, tumor necrosis factor-alpha; UCB, umbilical cord blood; UC-MSC, umbilical cord-derived mesenchymal stem cell; VABS-3, Vineland Adaptive Behavior Scales, Third Edition; WJ-MSC, Wharton’s jelly-derived mesenchymal stem cell.

Design Element	Specification	Rationale/Evidence	Refs.
Trial arms (minimum)	Three-arm RCT: (1) purified UC-MSC vs. (2) heterogeneous allogeneic UCB (CBMNC, TNCC-standardized) vs. (3) placebo	Directly resolves whether efficacy resides in the purified MSC or in the composite UCB population; the one pediatric ASD trial to add a purified MSC population within the same protocol (Lv et al.) showed a larger CARS reduction with CBMNC + UC-MSC than with CBMNC alone, but as an add-on design it cannot isolate the contribution of UC-MSC	[20]
Arm 1 product specification (purified UC-MSC)	Manufactured to ISCT minimal criteria with a validated immunomodulation potency assay	Defines a characterized, reproducible single-population product; potency-linked release testing	[33,88]
Arm 2 product specification (heterogeneous UCB)	CBMNC, TNCC-standardized as in the Dawson Phase II protocol	Represents the composite-population comparator; CD14^+^ monocyte proposed as active ingredient (DUOC-01 mechanism)	[130,140]
Mechanistic basis	CD14^+^ monocyte therapeutic-ingredient hypothesis; Duke group rejected CD34^+^ dosing on mechanistic grounds	DUOC-01 (cord blood monocyte-derived) accelerated remyelination and upregulated PDGF-AA; Duke reply and Vinmec critique frame the source question	[38,138,140]
Patient stratification	Age (3–7 vs. 8–12 yr), inflammatory phenotype, and clinical severity	Aligns with the developmental age-window principle and inflammatory-subtype heterogeneity	[131,155]
Outcome battery	Behavioral (CARS-2, VABS-3, ATEC), neurophysiological (EEG spectral analysis, eye-tracking OMI), and biological markers (serum IFN-γ, IL-1β, IL-6, TNF-α)	Pre-specified multi-domain battery addresses the divergent instrument sets used across existing trials, which limit cross-trial inference (Section 8.1)	[18]
Dose & schedule	Intravenous at a Phase I-established dose, with multi-dose protocols	Modeled on the CATO adaptive multi-dose design framework	[165]
Source diversity of prior trials	Existing UC-derived ASD trials span allogeneic CBMNC, autologous UCB, allogeneic UC-MSC, autologous BM-MSC, and WJ-MSC	Heterogeneous sources across the published trials preclude source-specific efficacy inference without a head-to-head design	[20,21,22,46,127,130,131,166]

## Data Availability

No new data were created or analyzed in this study. Data sharing is not applicable to this article.

## Abbreviations

The following abbreviations are used in this manuscript:

3D	Three-dimensional
AAV	Adeno-associated virus
ABA	Applied behavior analysis
ABAS	Adaptive Behavior Assessment System
ABC	Aberrant Behavior Checklist
ABC-CT	Autism Biomarkers Consortium for Clinical Trials
ACB	Autologous cord blood
AD	Adipose tissue
ADDM	Autism and Developmental Disabilities Monitoring
ADI-R	Autism Diagnostic Interview-Revised
AD-MSC	Adipose tissue-derived mesenchymal stromal cell
Akt	Protein kinase B
AM	Amniotic membrane
Anti-HBc	Hepatitis B core antibody
Anti-HBs	Hepatitis B surface antibody
APRICOT	Anaesthesia PRactice In Children Observational Trial
ARDS	Acute respiratory distress syndrome
ASD	Autism spectrum disorder
ATEC	Autism Treatment Evaluation Checklist
ATMP	Advanced therapy medicinal product
B7-H1	B7 homolog 1
Bax	Bcl-2-associated X protein
BBB	Blood–brain barrier
BDNF	Brain-derived neurotrophic factor
BEST	Biomedical Excellence for Safer Transfusion
BM	Bone marrow
BMMNC	Bone marrow mononuclear cells
BM-MSC	Bone marrow-derived mesenchymal stromal cell
CARS	Childhood Autism Rating Scale
CARS-2	Childhood Autism Rating Scale, Second Edition
Caspase	Cysteine-aspartic protease
CBMNC	Cord blood mononuclear cell
CBT	Cognitive behavioral therapy
CD	Cluster of differentiation
CDC	Centers for Disease Control and Prevention
CFU-F	Colony-forming unit-fibroblast
CGI	Clinical Global Impression
cGMP	Current Good Manufacturing Practice
CI	Confidence interval
CNS	Central nervous system
COVID-19	Coronavirus disease 2019
CP	Cerebral palsy
CSF	Cerebrospinal fluid
CUMS	Chronic unpredictable mild stress
Denver II	Denver Developmental Screening Test, Second edition
DMSO	Dimethyl sulfoxide
EBMT	European Society for Blood and Marrow Transplantation
EEG	Electroencephalography
EGF	Epidermal growth factor
EOWPVT	Expressive One Word Picture Vocabulary Test, Fourth edition
EP2	Prostaglandin E2 receptor subtype 2
EP4	Prostaglandin E2 receptor subtype 4
EV	Extracellular vesicles
FBS	Fetal bovine serum
FDA	Food and Drug Administration
FGF	Fibroblast growth factor
FIM	Functional Independence Measure
FLT1	Fms-related tyrosine kinase 1
FMEA	Failure mode and effects analysis
FoxP3	Forkhead box P3
GABA	Gamma-aminobutyric acid
GAG	Glycosaminoglycan
GAP-43	Growth-associated protein 43
GARS-II	Gilliam Autism Rating Scale, Second Edition
GDNF	Glial cell line-derived neurotrophic factor
GM-CSF	Granulocyte-macrophage colony-stimulating factor
GMFM-66	Gross Motor Function Measure-66
GMP	Good Manufacturing Practice
GVHD	Graft-versus-host disease
H&E	Hematoxylin and eosin
HBV	Hepatitis B virus
HCV	Hepatitis C virus
HGF	Hepatocyte growth factor
HIV	Human immunodeficiency virus
HLA	Human leukocyte antigen
HLA-ABC	Human leukocyte antigen class I (A, B, and C)
HLA-DR	Human leukocyte antigen-DR isotype
HLA-G	Human leukocyte antigen-G
HPL	Human platelet lysate
HTLV	Human T-cell lymphotropic virus
HUCPV	Human umbilical cord perivascular
ICS	Individualized combination supplements
ICV	Intracerebroventricular
ID	Intellectual disability
IDO	Indoleamine 2,3-dioxygenase
IFNG-AS1	Interferon gamma antisense RNA 1
IFN-γ	Interferon-gamma
IGF-1	Insulin-like growth factor-1
IGF-1R	Insulin-like growth factor-1 receptor
IL	Interleukin
IL-1Ra	Interleukin-1 receptor antagonist
iPSC	Induced pluripotent stem cell
IQ	Intelligence Quotient
ISAA	Indian Scale for Assessment of Autism
ISCT	International Society for Cellular Therapy
IT	Intrathecal
IV	Intravenous
JAKE	Janssen Autism Knowledge Engine
LPS	Lipopolysaccharide
MAPC	Multipotent adult progenitor cell
MCB	Master cell bank
MCP-1	Monocyte chemoattractant protein-1
MDC	Macrophage-derived chemokine
MHC	Major histocompatibility complex
MIA	Maternal immune activation
miR-21a-3p	MicroRNA-21a-3p
MMP	Matrix metalloproteinase
mRNA	Messenger RNA
MSC	Mesenchymal stromal cell
MSEC	Mental Synthesis Evaluation Checklist
mTOR	Mechanistic target of rapamycin
NAT	Nucleic acid testing
NF-κB	Nuclear factor kappa B
NGF	Nerve growth factor
NK	Natural killer
NLRP3	Nucleotide oligomerization domain-like receptor protein 3
NOD	Non-obese diabetic
NSE	Neuron-specific enolase
NT-3	Neurotrophin-3
OA	Osteoarthritis
OCT	Octamer-binding transcription factor
OMI	Oculomotor Index of Gaze to Human Faces
PACT	Production Assistance for Cellular Therapies
PDD	Pervasive Developmental Disorder
PDGF	Platelet-derived growth factor
PDMS-2	Peabody Developmental Motor Scales-2 Gross Motor Quotient
PET	Positron emission tomography
PGE2	Prostaglandin E2
pHPL	Pooled human platelet lysate
PI3K	Phosphoinositide 3-kinase
Poly I:C	Polyinosinic-polycytidylic acid
PTEN	Phosphatase and tensin homolog
PV	Perivascular
RBS-R	Repetitive Behavior Scale-Revised
RCT	Randomized controlled trial
RDoC	Research Domain Criteria
REX1	Reduced expression protein 1
RORγt	Retinoic acid receptor-related orphan nuclear receptor gamma t
RR	Relative risk
SA	Sub-amnion
SARS-CoV-2	Severe acute respiratory syndrome coronavirus 2
SCID	Severe combined immunodeficiency
SCQ	Social Communication Questionnaire
SEED	Study to Explore Early Development
SMD	Standardized mean difference
SOX	SRY-box transcription factor
SRS	Social Responsiveness Scale
SSEA	Stage-specific embryonic antigen
SSRI	Selective serotonin reuptake inhibitor
STR	Stirred-tank bioreactor
SYP	Synaptophysin
TARC	Thymus and activation-regulated chemokine
TGF-β1	Transforming growth factor-beta 1
Th	T-helper
TLR	Toll-like receptor
TNCC	Total nucleated cell count
TNF-α	Tumor necrosis factor-alpha
Treg	Regulatory T cell
TREM-2	Triggering receptor expressed on myeloid cells-2
TRM	Treatment-related mortality
TSPO	Translocator protein
UCB	Umbilical cord blood
UC-MSC	Umbilical cord-derived mesenchymal stromal cell
US	United States
USV	Ultrasonic vocalization
VABS-3	Vineland Adaptive Behavior Scales, Third Edition
VABS-II	Vineland Adaptive Behavior Scales, Second Edition
VEGF	Vascular endothelial growth factor
VPA	Valproic acid
WeeFIM	Functional Independence Measure for Children
WHO	World Health Organization
WJ	Wharton’s jelly
WJ-MSC	Wharton’s jelly-derived mesenchymal stromal cell
WMD	Weighted mean difference
WOMAC	Western Ontario and McMaster Universities Osteoarthritis Index total outcomes
